# A cryo-electron microscopy structure of yeast Pex5 in complex with a cargo uncovers a novel binding interface

**DOI:** 10.1242/jcs.263890

**Published:** 2025-06-26

**Authors:** Lior Peer, Orly Dym, Nadav Elad, Asa Tirosh, Jossef Jacobovitch, Ehud Sivan, Mor Angel, Shira Albeck, Maya Schuldiner, Yoav Peleg, Einat Zalckvar

**Affiliations:** ^1^ Department of Molecular Genetics, The Weizmann Institute of Science, Rehovot 7610001, Israel; ^2^ Life Sciences Core Facilities (LSCF), The Weizmann Institute of Science, Rehovot 7610001, Israel; ^3^ Department of Chemical Research Support, The Weizmann Institute of Science, Rehovot 7610001, Israel; ^4^ The Mina and Everard Goodman Faculty of Life Sciences, Bar-Ilan University, Ramat-Gan 5290002, Israel

**Keywords:** Peroxisome, Protein targeting, Pex5, Eci1, Dci1, Piggybacking, PTS1, *Saccharomyces cerevisiae*, Cryo-EM, Protein–protein interaction

## Abstract

Proper protein targeting to organelles is crucial for maintaining eukaryotic cellular function and homeostasis. This necessity has driven the evolution of specific targeting signals on proteins and the targeting factors that recognize them. A prominent example is peroxisomal matrix proteins, most of which depend on the targeting factor Pex5 to localize and function correctly. Although most Pex5 cargoes contain a peroxisomal targeting signal type 1 (PTS1), they are not all targeted similarly. Some undergo priority targeting, facilitated either by stronger binding to specific subsets of PTS1 signals or by additional interaction interfaces. These observations highlight the extensive complexity of Pex5-mediated targeting. In this study, we reveal that the *Saccharomyces cerevisiae* (yeast) matrix protein Eci1 can reach peroxisomes and bind Pex5 in the absence of PTS1. By solving the structure of the yeast Pex5–Eci1 complex using cryo-electron microscopy, we identified additional binding interfaces. Our findings provide new insights into the versatile interactions between Pex5 and its cargo, Eci1. More broadly, this work highlights the intricate, dynamic nature of the interactions between cargo factors and their cargoes to meet the complex environment within eukaryotic cells.

## INTRODUCTION

Cells produce a plethora of proteins, many of which require precise localization to specific cellular compartments to maintain proper function and cellular homeostasis (Aviram and Schuldiner, 2017; Bykov et al., 2020). Mislocalized proteins can have severe cellular implications. First, the absence of the protein from its target compartment leads to loss of function. Second, the protein might be mislocalized to another compartment, causing a toxic gain of function. Finally, the protein might aggregate, resulting in cellular stress and cytotoxicity. To avoid these deleterious effects, cells have evolved intricate pathways to ensure correct spatial and temporal protein targeting.

One cellular compartment that depends heavily on proper protein localization is the peroxisome. Peroxisomes are vital organelles that perform key metabolic functions and processes, such as fatty acid catabolism, detoxification of reactive oxygen species (ROS) and plasmalogen synthesis (Wanders et al., 2023). Aberrant peroxisomal function or the absence of peroxisomes results in severe diseases, including Zellweger spectrum disorders (Argyriou et al., 2016; Waterham et al., 2016). Moreover, recent studies have linked peroxisomal dysfunction to widespread pathological conditions like diabetes, neurodegeneration, cancer and more (Zalckvar and Schuldiner, 2022; Wanders et al., 2023).

Given that peroxisomes lack their own genome, all peroxisomal matrix (lumen) proteins are encoded by nuclear genes, synthesized on cytosolic ribosomes, and must be imported into the organelle. This process is mediated by canonical peroxisomal targeting signals (PTS1 or PTS2), recognized by the cargo factors Pex5 and Pex7, respectively (Walter and Erdmann, 2019).

Whereas most Pex5 cargo possess a PTS1, it is evident that many Pex5 cargo proteins do not have a PTS1 (Klein et al., 2002; Kempiński et al., 2020; Yifrach et al., 2022). Moreover, some PTS1 proteins can still be targeted even when their PTS1 is masked, implying that it is not exposed in the cytosol and/or unavailable for Pex5 recognition (Kempiński et al., 2020; Yifrach et al., 2022). These findings suggest that Pex5 can mediate cargo targeting through PTS1-independent mechanisms.

One such example is the yeast Eci1 (Δ3, Δ2-enoyl-CoA isomerase 1), an enzyme that is essential for the β-oxidation of unsaturated fatty acids in peroxisomes. Although Eci1 contains a PTS1 (Geisbrecht et al., 1998; Gurvitz et al., 1998), its targeting to peroxisomes can occur independently of this signal. The PTS1-independent targeting mechanism of Eci1 has been a subject of debate (Geisbrecht et al., 1999; Karpichev and Small, 2000; Yang et al., 2001). Two hypotheses for the enigmatic targeting mechanism of Eci1 have been suggested – that Eci1 has both a PTS1 and a PTS2 (Karpichev and Small, 2000; Yang et al., 2001) or that it might ‘piggyback’ on its paralog Dci1 (i.e. being co-imported to peroxisomes by utilizing the PTS1 of Dci1) (Geisbrecht et al., 1999; Karpichev and Small, 2000; Yang et al., 2001). However, neither hypothesis has been conclusively proven, leaving the mechanisms of Eci1 targeting to peroxisomes in the absence of its own PTS1 a mystery.

Here, we demonstrate that Eci1 fused to monomeric NeonGreen (mNG) is localized to peroxisomes, even though its PTS1 signal is masked by the mNG fluorophore. We show that under these circumstances, Eci1 still relies on Pex5 and that its targeting cannot be fully accounted for by piggybacking on Dci1. In addition, we show that Eci1 lacking its PTS1 can still bind to Pex5 *in vitro*, suggesting an additional non-canonical binding site between Pex5 and Eci1. To explore this further, we co-expressed the full-length yeast Pex5 with Eci1 and purified the complex. Using cryo-electron microscopy (cryo-EM), we resolved the structure of the Pex5–Eci1 complex, revealing a novel binding interface in addition to the PTS1-binding interface.

The identification of an additional binding interface between Pex5 and its cargo is crucial for understanding how cargo proteins interact with Pex5. This discovery highlights the inherent complexity of protein targeting within this highly regulated and multifaceted metabolic organelle, demonstrating that Pex5–cargo protein interactions are even more multifaceted than previously conceived, even in the case of PTS1–cargo proteins.

## RESULTS

### Eci1 is targeted to peroxisomes when its PTS1 is masked by a fluorophore

In recent years, it has become apparent that Pex5-mediated targeting of proteins to the peroxisome matrix is more complex than previously thought (Effelsberg et al., 2016; Yifrach et al., 2016; Rymer et al., 2018; Gabay-Maskit et al., 2020). One example of this complexity is Eci1, which has been shown to localize to peroxisomes even when its canonical PTS1 targeting signal is absent (Karpichev and Small, 2000; Yang et al., 2001). This PTS1-independent targeting led to the suggestions that either Eci1 has a PTS2 and can use Pex7 in addition to Pex5, or that Eci1 is piggybacking on its paralog Dci1 (Geisbrecht et al., 1999; Karpichev and Small, 2000; Yang et al., 2001). Nevertheless, how Eci1 is targeted to peroxisomes in the absence of a PTS1 has remained a mystery.

**Fig. 1.**
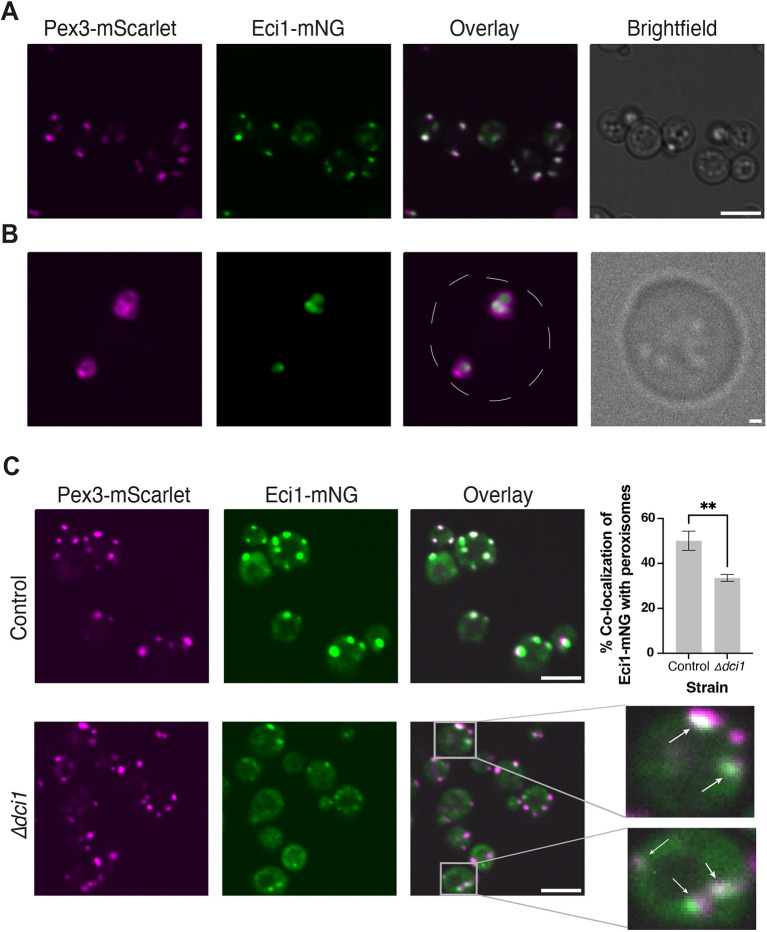
**Eci1 is localized to peroxisomes when its PTS1 is masked by mNeonGreen and when its paralog is absent.** (A) Fluorescence microscopy images of Eci1–mNeonGreen (mNG), showing colocalization with peroxisomes (Pex3–mScarlet) when tagged at the C-terminus. Scale bar: 5 μm. (B) High-resolution (SORA) fluorescence microscopy images showing the sub-peroxisomal localization of Eci1–mNG in a Δ*pex11* background, following growth in oleate as a sole carbon source. Eci1 is in the peroxisomal matrix. Dashed line highlights the cell border. Scale bar: 500 nm. Images in A and B are representative of three technical experimental repeats. (C) Fluorescence microscopy images demonstrating that in the Δ*dci1* background, the signal of Eci1–mNG is greatly reduced (as shown in Fig. S1B), yet peroxisomal localization of Eci1 is still observed (indicated by white arrows). Scale bars: 5 μm. The graph represents mean±s.d. for one experiment with three technical repeats (*n*=3) in each strain. **P<0.01 (unpaired two-tailed *t*-test).

Intrigued by this unresolved question, we first masked the PTS1 of Eci1 by genomically integrating an mNG-encoding gene downstream of *ECI1*. The synthesized protein is C′ tagged with mNG, which prevents Pex5 from binding to the PTS1, as that must be at the extreme C-terminus of a protein for functional recognition (Gould et al., 1989). Consistent with previous observations, we found that Eci1–mNG still localizes to peroxisomes (Fig. 1A). This is in stark difference to other PTS1-dependent peroxisomal proteins, which lose their peroxisomal localization when their PTS1 is masked with mNG (Fig. S1A). Notably, this behavior is more similar to that of Pox1, a well-studied Pex5 cargo that utilizes PTS1-independent targeting (Klein et al., 2002; Kempiński et al., 2020).

The diameter of yeast peroxisomes is ∼150 nm, which is smaller than the diffraction limit of light (∼250 nm). As a result, our fluorescence microscopy images showing colocalization of Eci1-mNG and peroxisomes might hypothetically reflect mislocalization of Eci1–mNG to the peroxisomal surface or nearby organelles. To confirm that Eci1–mNG is indeed targeted to the peroxisome matrix, we increased the size of peroxisomes by deleting the *PEX11* gene and growing the cells on oleate as a sole carbon source (Erdmann and Blobel, 1995; Yifrach et al., 2022), alongside imaging with a high-resolution microscopy system (SORA). Indeed, we were able to affirm that Eci1–mNG was correctly localized to the peroxisomal matrix (Fig. 1B). These findings demonstrate that masking the PTS1 of Eci1 does not prevent its proper targeting to the peroxisome matrix.

As previously proposed (Yang et al., 2001), we hypothesized that Eci1 might be ‘piggybacking’ on its paralog, Dci1. To test this, we deleted *DCI1* and examined the effect on the peroxisomal localization of Eci1–mNG. The deletion of *DCI1* resulted in a significant decrease in the total Eci1–mNG signal (quantification shown in Fig. S1B). Strikingly, we observed an impairment, but not complete abolishment, of the peroxisomal localization of Eci1–mNG (Fig. 1C; Fig. S1C). This observation supports the existence of an alternative mechanism by which Eci1 can still be targeted to peroxisomes when its PTS1 is masked and in the absence of its paralog.

### Pex5 is the only cargo factor targeting Eci1

One possible mechanism by which Eci1–mNG is targeted to peroxisomes in the absence of Dci1 could involve a cargo factor other than Pex5. To explore whether an alternative cargo factor facilitates Eci1 targeting when its PTS1 is masked and Dci1 is absent, we tested several hypotheses.

**Fig. 2.**
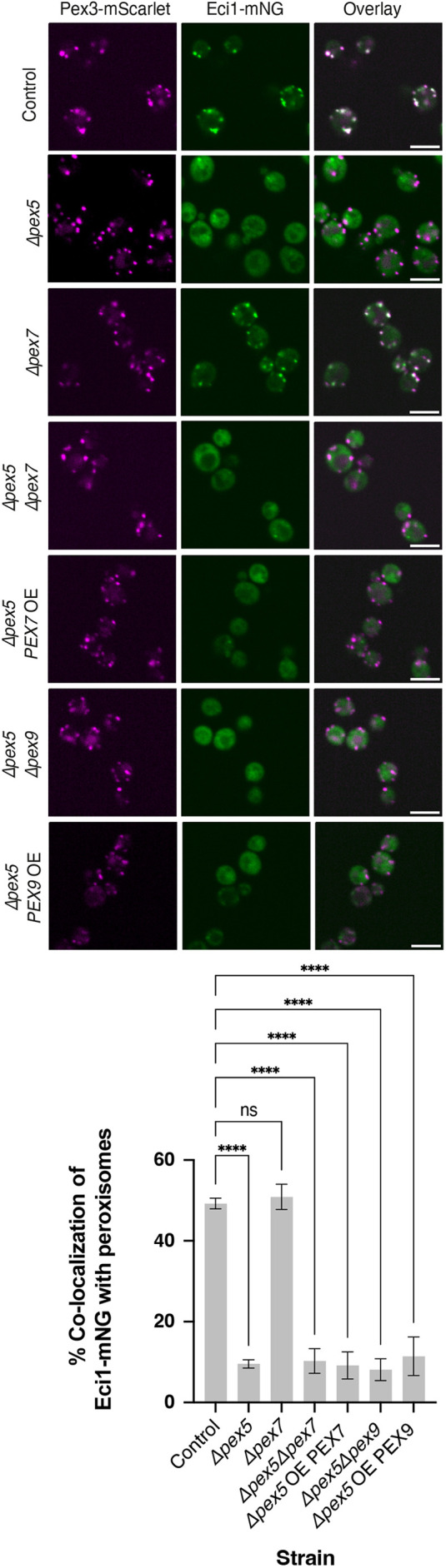
**Eci1 is targeted solely by Pex5.** Fluorescence microscopy images of different genetic backgrounds were examined to determine how Eci1 is targeted to peroxisomes and demonstrate a targeting mechanism that is dependent on Pex5 but independent of Pex7 and Pex9. The control and Δ*pex7 strains* show the same extent of colocalization with the peroxisomal marker. In contrast, the rest of the strains show a significant reduction in colocalization compared to the control. Scale bars: 5 μm. The graph represents mean±s.d. for one experiment with three technical repeats (*n*=3) in each strain. ****P<0.0001 (one-way ANOVA with Dunnett's correction for multiple comparisons).

First, it has been suggested that Eci1 contains a PTS2 (Karpichev and Small, 2000). To examine this, we deleted *PEX7*, the cargo factor responsible for targeting PTS2-containing proteins, both alone and in the Δ*pex5* genetic background*.* As expected, the PTS2 protein Pot1 failed to localize to peroxisomes in the absence of Pex7 (Fig. S1E). However, Eci1–mNG remained localized to peroxisomes (Fig. 2; Fig. S1D), implying that Eci1 does not rely on the Pex7–PTS2 targeting pathway. Although Pex7 was found to be unnecessary, we wondered whether it might still be sufficient for Eci1 targeting. To test this, we overexpressed Pex7 in the absence of Pex5 but observed no impact on Eci1–mNG localization (Fig. 2; Fig. S1D). These results indicate that, under the conditions of our assay, Pex7 is neither necessary nor sufficient for the targeting of Eci1–mNG to peroxisomes.

Next, we explored whether Pex9, which targets a specific set of PTS1 proteins to the peroxisomal matrix (Effelsberg et al., 2016; Yifrach et al., 2016, 2023), might play a role in Eci1–mNG targeting. To test this, we deleted or overexpressed *PEX9* in the Δ*pex5* background. Consistent with previous findings (Yifrach et al., 2016), GFP–Mls1 colocalized with peroxisome when Pex9 was over-expressed in the absence of Pex5 (Fig. S1E). We did not observe an effect of manipulating Pex9 on Eci1-mNG peroxisomal localization (Fig. 2). These findings indicate that Pex9 is neither necessary nor sufficient for the targeting of Eci1-mNG. Taken together, we conclude that Pex5 is the sole cargo factor mediating Eci1 targeting to peroxisomes under the conditions tested.

### Eci1 directly binds Pex5 in the absence of its PTS1

**Fig. 3.**
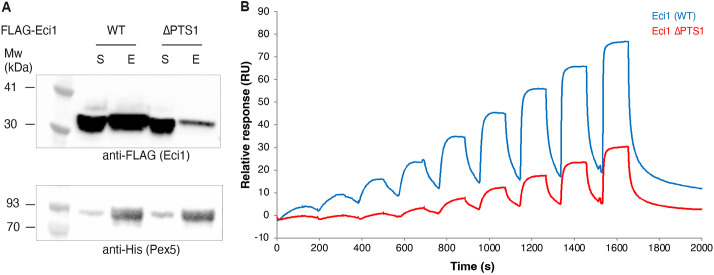
**Eci1 can bind Pex5 in the absence of its PTS1.** (A) Western blot analysis of *in vitro* His-tag pulldown of Pex5 WT with either Eci1 (WT) or Eci1 lacking its peroxisomal targeting sequence 1 (ΔPTS1). Blots were incubated with either anti-FLAG (upper blot), to detect FLAG–Eci1, or anti-His, to detect His–SUMO–Pex5 (lower blot). When the PTS1 is abolished, Eci1 shows a reduced but still clear ability to bind Pex5 *in vitro*. S, soluble fraction; E, elution fraction. Blot representative of three experimental repeats. (B) Overlay of two Biacore sensorgrams comparing the respective binding of Eci1 WT (blue) and Eci1ΔPTS1 (red) to immobilized Pex5 (on two separate channels). Binding was assessed in single-cycle kinetics mode without dissociation of the bound proteins using growing concentrations of each of the analytes (1.95 nM, 3.91 nM, 7.81 nM, 15.63 nM, 31.25 nM, 62.5 nM, 125 nM, 250 nM and 500 nM). RU, response units, which are proportional to the mass of analyte bound to the surface-bound protein.

To examine whether Eci1 directly binds to Pex5 in a PTS1-independent manner, and in the absence of Dci1 or any other yeast proteins, we employed a co*-*expression system in *Escherichia coli*. In this system, Pex5 was co-expressed with either wild-type Eci1 (WT) or Eci1 lacking the three most C-terminal amino acids (ΔPTS1). We performed a pull-down assay, capturing Pex5 and analyzing the presence of Eci1. Whereas removal of the PTS1 motif reduced the interaction between Eci1 and Pex5, direct binding was still detected (Fig. 3A). To further evaluate this interaction, we examined the binding between the purified proteins *in vitro*. Pex5, Eci1 WT and Eci1ΔPTS1 were individually expressed and purified from *E. coli* (Fig. S2A,B). Each purified protein was analyzed by dynamic light scattering (DLS) to ensure the absence of aggregation (Fig. S2B).

We employed surface plasmon resonance (SPR), a technique for measuring protein–protein interactions in real time, using Biacore to investigate the direct binding of Pex5 to either Eci1 WT or Eci1ΔPTS1. Pex5 was immobilized on two channels of a sensor chip via amine coupling, which covalently links exposed primary amines on the protein to the activated surface. To assess binding, we tested two forms of Eci1 – WT and the PTS1-deleted variant (ΔPTS1). In a typical SPR experiment, the analyte flows over the immobilized protein, allowing real-time monitoring of binding kinetics. Following this, a dissociation phase occurs as the analyte naturally washes away. However, in this case, the Pex5–Eci1 complex was highly stable and required a strong chemical wash (NaOH) for removal. Unfortunately, NaOH damaged Pex5, limiting sensor chip reusability. To circumvent this issue, we used single-cycle kinetics (SCK), allowing multiple injections without a strong wash step in between. Increasing concentrations of Eci1 (WT or ΔPTS1, each tested separately on different channels) ranging from 1.95 nM to 500 nM were injected over the immobilized Pex5. Remarkably, both Eci1 WT and Eci1ΔPTS1 exhibited direct binding to Pex5 (Fig. 3B), indicating that Eci1 can still interact with Pex5 in the absence of its PTS1. Notably, whereas the Eci1 WT–Pex5 interaction was detectable even at the lowest tested concentration (1.9 nM), Eci1ΔPTS1 required concentrations above 15 nM to show binding. This suggests that although Eci1 can interact with Pex5 without PTS1, the interaction is weaker compared to that of the WT form.

When analyzing the binding kinetics, the Pex5–Eci1 WT interaction did not fit a simple 1:1 binding model. Instead, using a bivalent interaction model, we identified an initial high-affinity binding event (*K*_D_≈0.15 µM) followed by a weaker secondary interaction (Fig. S2C, upper graph). In contrast, the Pex5–Eci1ΔPTS1 interaction did not conform to either a 1:1 or bivalent model, indicating a more complex mechanism of binding (Fig. S2C, bottom graph).

Taken together, the SPR results demonstrate that Eci1ΔPTS1 can still bind to Pex5 in the absence of its canonical PTS1, strongly supporting the existence of an additional, non-PTS1 interaction interface between Pex5 and Eci1.

### Identification of the non-canonical interface between Eci1 and Pex5

**Fig. 4.**
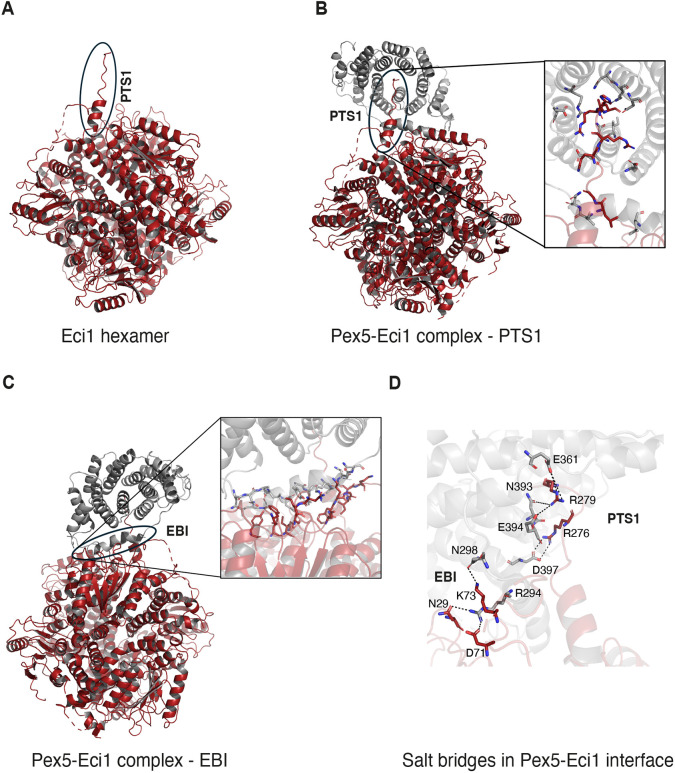
**The cryo-EM structure of Pex5 in complex with an Eci1 hexamer highlights a PTS1 binding interface and a novel EBI.** (A) The Eci1 hexamer consists of six subunits depicted in red ribbon. A black circle indicates the additional 13 residues at the C-terminal region (^268^FRQLGSKQRKHRL^280^), which include the tripeptide ^278^HRL^280^ that defines the PTS1 peroxisomal targeting signal. This C-terminal region is observed in only one Eci1 subunit that interacts with Pex5. (B) The C-terminal domain of Pex5 and the Eci1 hexamer complex are shown as ribbons. The C-terminal domain of Pex5 comprises the TPR domain represented in a gray ribbon and the Eci1 hexamer in a red ribbon. The additional C-terminal region in the Eci1 subunit is indicated by a black circle. A closer view of the interaction between the C-terminal segment of Eci1 and the TPR domain of Pex5 is depicted in the black box, with residues shown as sticks (with nitrogen in blue, carbon in gray for Pex5 and red for Eci1, and oxygen in red for Pex5 and orange for Eci1). (C) An additional 21 residues at the C-terminal domain of Pex5 (^276^LVNDDLNLGEDYLKYLGGRVN^296^) representing the EBI that had not been observed previously are highlighted by a black circle. A closer view of the novel EBI involving residues from the C-terminal segment of Eci1 interacting with residues from the TPR3 domain of Pex5 is shown as sticks (color scheme as in B). (D) Electrostatic complementarity relies on multiple salt-bridge interactions between specific amino acid residues from the PTS1 and the EBI interfaces. Residues are labeled and shown as sticks (colorings as in B).

To characterize the binding interfaces between Pex5 and Eci1, we co-expressed full-length yeast Pex5 and Eci1 in *E. coli*, co-purified the complex by size exclusion chromatography (SEC) (Fig. S3A), and visualized the complex using single-particle cryo-EM (Fig. S3B–E). The cryo-EM reconstruction of the Pex5–Eci1 complex dataset revealed that Eci1 forms a hexamer (Fig. 4A), with a single subunit bound to a Pex5 monomer (Fig. 4B). Although the Eci1 hexamer was readily resolved to high resolution (Fig. S3B), the electron density of the bound Pex5 was poorly resolved and showed conformational heterogeneity relative to Eci1. To further investigate, we performed two 3D variability analyses aimed at understanding the stoichiometry and conformational landscape of the Eci1–Pex5 complex (Fig. S3B,C, summarized in Fig. S3D). Initial variability analysis, using a wide spherical mask around the complex, suggested that the dataset predominantly contained singly bound Pex5 complexes, with a few subsets showing two or more Pex5 molecules bound to Eci1 subunits. Notably, all Pex5 densities adopted a similar orientation relative to the Eci1 hexamer (Fig. S3B). Further variability analysis, using a mask that included both the Eci1 hexamer and a single Pex5 molecule, revealed that Pex5 was bound in multiple conformations (Fig. S3C). Refined 3D reconstructions of the separated subsets showed that Pex5 formed a variable interface with one Eci1 subunit. In some subsets, the interface consisted of only two attachment points with a gap in between them, whereas in others, a more extensive, continuous interface was formed. As a result, the orientations that Pex5 adopted relative to Eci1 were limited, leading to poor resolution of Pex5 when bound at only two points, but a better resolution when the more extensive interface was formed. Intermediate conformations were also observed, where partial density is formed in the gap.

To gain molecular insight, we focused on the subset where Pex5 and the Eci1–Pex5 interface was most stable, containing only a single Eci1 subunit bound to Pex5. The maps from this subset were refined to 2.7 Å (1 Å=0.1 nm) globally, whereas Pex5 and the interface were resolved to 2.9 Å after local refinement of this region (Figs S3D,E, S4A,B).

At the atomic level, Eci1 consists of an N-terminal core domain (M1–N200) with a spiral fold topology and a C-terminal region (M201–L280) forming an α-helical trimerization domain. In the trimerization domain, three Eci1 subunits formed a characteristic trimeric disk with strong interactions between the three subunits. Two of these trimeric disks combined to create a hexamer (Fig. 4A). Given that the 3D refinement was performed without applying symmetry, the six Eci1 subunits exhibited variations in the resolved residues. Specifically, five subunits contained residues I5–Q270, E4–Q270, I5–Q270, E4–Q270 and I5–Q270. The Pex5-bound Eci1 subunit contains a distinct segment at its C-terminal end (^268^FRQLGSKQRKHRL^280^), highlighted with a black circle and labeled as PTS1 in Fig. 4A. Electron density for this C-terminal segment, including the PTS1, was absent in the other five Eci1 subunits that did not bind Pex5. Moreover, this unique C-terminal segment has not been observed in any previously known apo-Eci1 structures. Within this segment, the tripeptide ^278^HRL^280^ defines the PTS1 peroxisomal targeting signal, and this region adopted a specific conformation upon interacting with Pex5. Interestingly, although the bound Pex5 electron density was poorly resolved and appeared conformationally heterogeneous in relation to Eci1, the PTS1-binding interface was clearly identified in all ten clusters, underscoring its importance (Fig. S3C).

The Pex5 structure (L276–F612) comprises the tetratricopeptide repeat (TPR) domain (N314–S553) with seven TPR repeats, each with 34 residues (α1–α2, α3–α4, α5–α6, α7–α8, α9–α10, α11–α12 and α13–α14) (Fig. S4C, and represented as a gray cartoon in Fig. 4B,C). This structure also includes the PTS1–cargo binding region, a known feature of the Pex5 receptor TPR domain. Notably, cryo-EM maps showing the most stable Eci1–Pex5 interface contained only a single Eci1 subunit bound to Pex5. Interestingly, there was electron density for an additional 21 residues at the Pex5 N-terminal region (^276^LVNDDLNLGEDYLKYLGGRVN^296^), which we name the Eci1-binding interface (EBI). This interface had not been observed previously in any other known Pex5 structures. The C-terminal region of Eci1 was present in only one of the six subunits and interacted with specific residues from the TPR3, TPR6 and TPR7 repeats of Pex5. Cryo-EM maps of the Pex5–Eci1 complex, containing the full-length Pex5, did not show electron density for the N-terminal domain (NTD) of Pex5 (up to residue E275). This suggests that the NTD of Pex5 is flexible within the complex, making it difficult to visualize using cryo-EM. Importantly, the Pex5–Eci1 structure (illustrated in Fig. 4B,C) revealed three interfaces. Two of these served as anchoring points at the two extremes of the Pex5–Eci1 interface, which was observed in all ten clusters (Fig. S3C). An extensive protein–protein interface is facilitated by two binding surfaces. The first involves the C-terminal segment (^268^FRQLGSKQRKHRL^280^) of one out of the six Eci1 subunits, which includes the PTS1 (^278^HRL^280^) and interacted with the TPR3, TPR6 and TPR7 domains of Pex5 (Fig. 4B). This interaction was observed in all ten clusters (Fig. S3C). The PTS1-containing segment was located within a deep central cavity of the TPR domain and was reinforced by a network of hydrogen bonds formed between ^278^HRL^280^ and residues in TPR3, TPR6 and TPR7 of Pex5. Among these interactions, a salt bridge was formed between R279 of Eci1 and E361, N393 and E394 of Pex5 (Fig. 4D). Additional interactions involved the C-terminal segment of Eci1, specifically ^268^FRQLGSKQRKHRL^280^, with residues in the TPR3 domain of Pex5, along with a few residues (L288, L291, G292, V295, and N296) from the additional N-terminal region of Pex5 (Fig. 4B).

Our structural analysis revealed a previously unidentified binding interface – the EBI, consisting of an elongated stretch of 21 mostly variable residues in the NTD of Pex5 (^276^LVNDDLNLGEDYLKYLGGRVN^296^; Fig. 4C,D; Fig. S4C). This stretch interacted with a distinct interface on Eci1, as well as with a few residues from the C-segment of Eci1 (F268, L271, and G272), suggesting that the EBI and the PTS1-binding regions work together to facilitate the interaction between Pex5 and Eci1. Another interaction point between Pex5 and Eci1, present in all ten clusters, was located on the opposite side of the complex from the PTS1-binding site, involving residues ^419^IKQQDKFQKEK^430^ of Pex5 (Fig. S3C). Interestingly, both this region and the EBI are the least conserved areas in the otherwise highly conserved C-terminal domain (CTD) of Pex5, as shown by ConSurf (Fig. S5A).

Focusing on the newly identified EBI, we observed that the three segments of Eci1 played crucial roles in forming core interactions with the NTD of Pex5, namely, ^29^NL^30^, ^65^FFSSGADFKGIAK^77^ and ^143^KVYLLYPFANL^153^. These latter two segments form a concave structure that accommodated the NTD of Pex5. The interactions within the EBI involved hydrogen bonds and salt bridges, which enhanced the stability and specificity of the Pex5–Eci1 interaction (Fig. 4C,D).

### Characteristics of the newly revealed EBI

To investigate the evolutionary conservation of amino acids in Eci1 and related proteins, we used the ConSurf server (Ashkenazy et al., 2016), which performs multiple sequence alignments of 150 homologous proteins and predicts amino acid conservation based on evolutionary history. This analysis revealed a high level of conservation among the amino acids that interacted with Pex5 (highlighted in maroon in Fig. S5A), emphasizing their importance in mediating the Pex5–Eci1 interaction. Additionally, ConSurf analysis of Pex5 showed that the CTD was highly conserved, whereas the NTD was more variable (Fig. S5A). Within the Pex5 CTD, the least conserved regions included the EBI and the anchoring point at the complex interface, encompassing residues ^419^IKQDDKFQKEK^430^.

However, these segments were conserved within *Saccharomyces* species, which is noteworthy given the unique role of yeast peroxisomes in the β-oxidation of fatty acids. Although peroxisomes are generally highly conserved, yeast peroxisomes are unique in that they are solely responsible for the β-oxidation of fatty acids. In most other organisms, like humans, β-oxidation occurs either in the mitochondria or peroxisomes depending on specific characteristics, such as fatty acid chain length, highlighting potential functional divergence in yeast enzymes involved in this pathway.

**Fig. 5.**
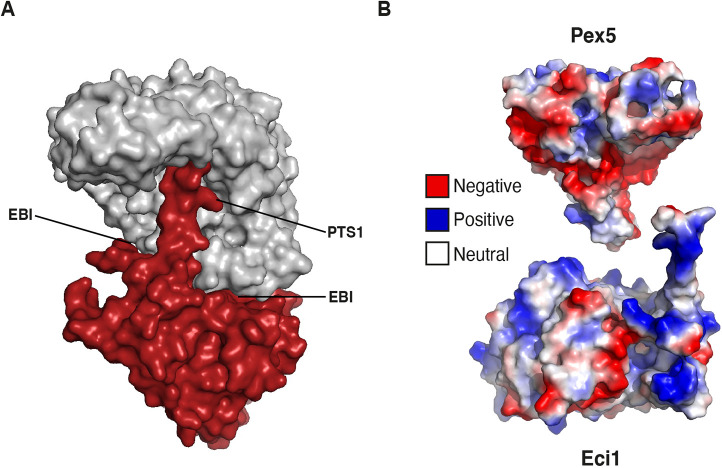
**The Pex5–Eci1 complex structure interfaces exhibit shape and charge compatibility.** (A) Surface representation of the TPR domain of Pex5 (gray) interacting with one subunit of the Eci1 hexamer (red). Two binding interfaces were identified: one involving the C-terminal PTS1 of Eci1 interacting with the TPR domain of Pex5, labeled as PTS1, and the newly identified EBI labeled as EBI. Both interfaces exhibit shape complementarity. (B) Electrostatic representation of the electropositive surface of the Eci1 subunit (bottom), which is complementary to the electronegative surface of the TPR domain of Pex5 (top). Electronegative surfaces are depicted in red, electropositive in blue, and neutral in white. The view in B is 180° rotation compared to A.

Structural analysis further revealed that the binding interfaces for PTS1 and EBI exhibited remarkable shape complementarity, with the concave surface of Eci1 closely aligning with the convex surface of the TPR domain of Pex5 (Fig. 5A).

In addition to shape complementarity, the Pex5–Eci1 interaction exhibited significant electrostatic complementarity. Specifically, the surface of Pex5 was predominantly electronegative (red), whereas that of Eci1 was largely electropositive (blue), as depicted in Fig. 5B. This electrostatic complementarity was mediated by a network of salt-bridge interactions between key amino acids in both proteins, which are therefore crucial for stabilizing the complex.

Negatively charged residues on Pex5, including N298, E361, N393, E394 and D397, form salt bridges with positively charged residues on Eci1, such as K73, R279 and R276. Conversely, negatively charged residues on Eci1, including N29 and D71, interact with the positively charged R294 on Pex5. Of particular importance, R276 on Eci1 forms multiple salt bridges with D397 on Pex5, while R279 on Eci1 interacts with E361, N393, and E394 on Pex5 (Fig. 4D). These salt bridges spanned both the PTS1 and EBI motifs, with the cooperative interactions of oppositely charged residues playing a crucial role in the overall stability of the Pex5–Eci1 complex.

The combination of shape complementarity and electrostatic complementarity, exemplified by the salt bridge interactions, significantly enhances the binding strength between Pex5 and Eci1, ensuring a stable interaction between the two proteins.

### The N-terminal segment of Eci1 has a dual role

Eci1 plays a crucial role in fatty acid metabolism by facilitating the oxidation of unsaturated fatty acids. Specifically, it catalyzes the conversion of 3E- and 3Z-enoyl-CoA thioesters to 2E-enoyl-CoA thioesters intermediates during the four-step oxidation pathway, a process that requires the binding of co-enzyme A (CoA). The structure of Eci1 bound to acetoacetyl-CoA (CAA) has been previously determined (PDB entry 4ZDB; Onwukwe et al., 2015) (Fig. S5B, in purple). CAA interacted with Eci1 through a network of hydrogen bonds involving residues S68, A70, F72, K73, N152 and N181, as well as hydrophobic interactions with residues D28, N29, L30, A32, F65, D71, I124 and P149. Structural alignment between the Eci1–Pex5 complex and the Eci1–CAA complex revealed that the newly identified NTD segment of Pex5, the EBI (^276^LVNDDLNLGEDYLKYLGGRVN^296^), occupied the same binding pocket as CAA in the Eci1–CAA complex (Fig. S5B). Furthermore, the interaction of Eci1 with both CAA and Pex5 involves many common residues, including N29, L30, F65, S68, D71, F72, K73, I124, P149 and N152 (Fig. S5B). This overlap suggests a potential competition or coordination between CoA and Pex5 for binding to Eci1.

### Pex5 interacts with different cargoes via distinct binding interfaces

Our discovery of an additional binding interface between Pex5 and a cargo protein containing a PTS1 motif is not unprecedented. This has previously been reported for human PEX5 binding to alanine-glyoxylate aminotransferase (AGXT) (Fodor et al., 2012) and the interaction between *Trypanosoma cruzi* (Tc)Pex5 and malate dehydrogenase (MDH) (Sonani et al., 2024 preprint). AGXT, a peroxisomal enzyme with a PTS1, forms an extensive interface with PEX5 through additional interfaces beyond the canonical PTS1-binding region. Similarly, Tc-derived Pex5 interacts with MDH via the canonical PTS1 peptide within the central cavity of its TPR domain, as well as through a second, additional interface.

**Fig. 6.**
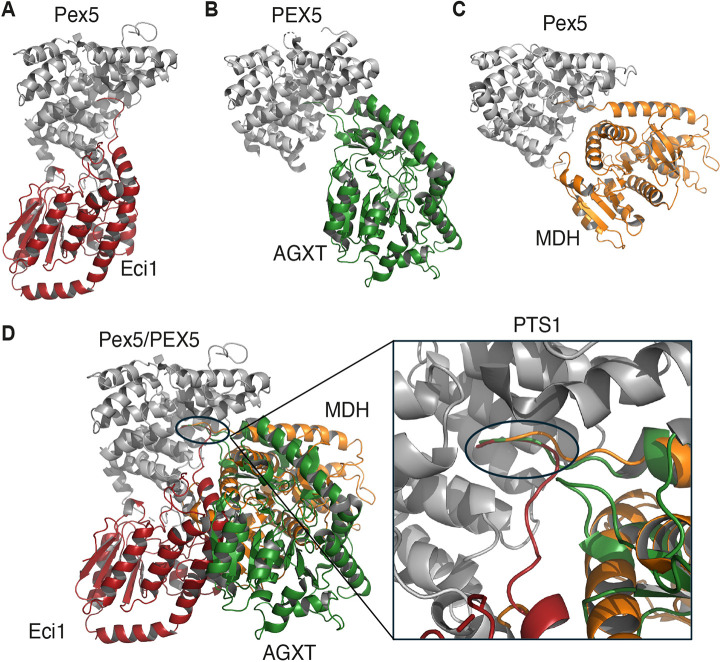
**The newly defined Pex5–Eci1 EBI differs from the binding interface of PEX5 with AGXT and Pex5 with MDH.** (A) The yeast Pex5–Eci1 complex is shown with gray and red ribbons, respectively. (B) The human PEX5–AGXT complex is shown with grey and green ribbons, respectively (PDB entry 3R9A). (C) The Pex5-MDH is shown with grey and orange ribbons, respectively (PDB entry 8GGH). (D) Alignment of the yeast Pex5–Eci1 complex with the PEX5–AGXT and Pex5–MDH structures reveals two binding interfaces. The PTS1, common to all three complexes, is highlighted by a black circle, while the second binding interface for Eci1, AGXT and MDH occupies different regions in the Pex5/PEX5 structures and involves distinct residues. A closer view of the PTS1 binding interface for Eci1, AGXT and MDH is provided in the black box. The resemblance of the PTS1 binding interface among these proteins is emphasized by the black circle.

To investigate whether the binding of Pex5 to Eci1 shares similarities with the PEX5–AGXT and Pex5–MDH interactions, we performed a structural alignment and interaction comparison between the TPR domain of yeast Pex5 bound to Eci1, human PEX5 bound to AGXT (PDB entry 3R9A; Fodor et al., 2012), and the TcPex5 bound to MDH (PDB entry 8GGH; Sonani et al., 2024 preprint) (Fig. 6). Although the yeast TPR domain shared relatively low sequence identity with its human and Tc counterparts (35% and 26%, respectively), the structural similarity was notable, with root mean square deviations (RMSDs) of 0.858 Å and 1.4 Å, respectively. Eci1 (in red, Fig. 6A), AGXT (in green, Fig. 6B), and MDH (in orange, Fig. 6C) each formed two binding interfaces with Pex5 (gray). Eci1 formed 97 hydrogen bond contacts with Pex5, compared to 29 for AGXT and 44 for MDH. In all three cases, the PTS1 region penetrated the TPR domain, with the tripeptides ^278^HRL^280^ (Eci1), ^390^KKL^392^ (AGXT), and ^321^SKL^323^ (MDH) aligning perfectly and adopting the same conformation (highlighted by the black circle and enlargement in Fig. 6D). These PTS1 motifs interacted with conserved residues in Pex5 – N393, A502, N503 and N530 in yeast Pex5, N415, A533, N534, and N568 in human PEX5, and N439, A556, N557 and N584 in TcPex5. These conserved interactions highlight the fundamental role of the PTS1 motif in PEX5-mediated protein recognition across species.

In contrast, the second binding interface differed significantly among the three complexes (Fig. 6D). In the PEX5–AGXT and Pex5–MDH interactions, this second interface was relatively localized and weak, involving only three residues, which varied between the two complexes. However, in the Pex5–Eci1 interaction, the second binding interface, the EBI, was considerably more extensive, encompassing 15 residues in Pex5 and 20 residues in Eci1 (Fig. 4C). On the Pex5 side, these residues included N278, D279, D280, L281, L283, G284, Y287, L288, Y290, L291, R294, V295, N296, G297 and N298, whereas in human PEX5, the interaction was mediated by R608, L609 and S612. This variability in binding interfaces highlights the adaptability of Pex5, which tailors its interactions to different binding partners, potentially offering flexibility to enable cargo-specific targeting.

The presence of distinct binding interfaces for different cargo proteins raises the possibility of additional proteins binding Pex5 and potentially being targeted to peroxisomes via a non-canonical mechanism. To explore this, we used AlphaFold2 (Jumper et al., 2021) to model the structure of Dci1, which was then aligned with the established Pex5–Eci1 complex structure (Fig. S5C). Interestingly, the distinct segment at the C-terminal of Eci1 (red), encompassing the PTS1 tripeptide (^268^FRQLGSKQRKHRL^280^), was oriented toward the TPR domain of Pex5. In contrast, the equivalent segment of the Dci1 model (green), when not bound to Pex5, was directed differently (highlighted in a black dashed circle).

Notably, several residues within the novel EBI interface of Eci1 that interacted with Pex5 are highly conserved in the paralog Dci1 (Fig. S5C). Specifically, residues L30, F65, S68, F72, P122, I124, Y145, P149, N152, P183, F268 and L271 in Eci1 correspond to L24, Y59, S62, F66, P116, I118, F139, P143, N146, P177, F259 and L262 in Dci1. These conserved residues, depicted as spheres within the dashed black circle and as sticks in the enlargement (black box), suggest that Dci1 might exploit a similar non-canonical binding to Pex5, as per Eci1.

## DISCUSSION

The role of PTS1 in Eci1 binding to Pex5 and facilitating its peroxisomal targeting is well established. However, the mechanism by which Eci1 is targeted to peroxisomes in the absence of its PTS1 has remained elusive (Geisbrecht et al., 1998, 1999; Gurvitz et al., 1998; Karpichev and Small, 2000; Yang et al., 2001). In this study, we demonstrate that Eci1 targeting to the peroxisomal matrix is exclusively dependent on Pex5 and is not mediated by either Pex7 or Pex9. Furthermore, we provide evidence that when the PTS1 of Eci1 is masked, its targeting cannot be solely attributed to piggybacking on its paralog Dci1. Our *in vivo* findings demonstrate that Eci1 is targeted to peroxisomes in a Pex5-dependent manner, even when its PTS1 is obscured by a fluorophore and in the absence of Dci1. These findings suggested the presence of an additional Pex5 binding interface between Pex5 and Eci1, which we resolved using cryo-EM.

The structures of the TPR domains of human PEX5 interacting separately with AGXT and SCP2 (thiolase) have been previously solved (Williams et al., 2011; Fodor et al., 2012). Recently, the structure of full-length *Trypanosoma cruzi* Pex5 and MDH has also been solved (Sonani et al., 2024 preprint). In this study, we co-expressed the full-length yeast Pex5 with Eci1, which enabled us to resolve and visualize previously uncharacterized residues in both proteins. This led to the discovery of a novel binding interface between Pex5 and Eci1, which aligns with our *in vivo* findings.

Why has this newly identified EBI remained undiscovered until now? Previous studies in *Saccharomyces cerevisiae* mutated residue N495 in Pex5 to argue that all Eci1 binding is through the PTS1-binding site. This point mutation was previously considered to abolish only the PTS1-binding domain. However, structural data, including our findings, suggest that mutating this crucial residue destabilizes the entire protein, leading to a misinterpretation of its role in Eci1 binding.

Our findings raise the intriguing possibility that additional peroxisomal matrix proteins bind and mediate their interaction with Pex5 through non-canonical binding mechanisms (such as in Fig. S5C). Recent studies have shown that other PTS1 proteins have more binding interfaces with Pex5. For instance, oxalyl-CoA synthetase (Pcs60) in yeast was found to still bind to Pex5 even in the absence of its PTS1 (Bürgi et al., 2023). Another example is the acyl-CoA oxidase Pox1, which has been suggested to harbor a non-canonical PTS, functioning as a signal patch within the fully folded protein (Klein et al., 2002; Kempiński et al., 2020). This phenomenon extends across different organisms. In humans, AGXT has been shown to interact with Pex5 independently of PTS1 (Fodor et al., 2012). Similarly, in *Arabidopsis thaliana*, catalase (CAT2) is targeted to peroxisomes in the absence of a PTS1 (Al-Hajaya et al., 2022). Recently, in *Trypasonoma cruzi*, MDH was shown to engage in a non-canonical interaction with Pex5 when complexed with Pex14 (Sonani et al., 2024 preprint). This non-canonical binding involves the intrinsically disordered region (IDR) in the N-terminal part of Pex5, similar to what has been observed in this study (data not shown). These examples highlight a broader relevance of PTS1-independent peroxisomal targeting across species.

Furthermore, these findings highlight the importance of studying full-length Pex5 in targeting studies, as its NTD might become structured upon cargo binding. This binding stabilizes the IDR and allows for a clearer resolution of the structure. Interestingly, in the case of two of the mentioned cargoes, the Pcs60 hexamer and the MDH tetramer, Pex5 only partially occupies the available binding interfaces on the cargo (Bürgi et al., 2023; Sonani et al., 2024 preprint). This is in line with our data, where only one Eci1 subunit of the hexamer is observed bound to Pex5 in most complexes (Fig. S3B). Further studies are required to understand the nature of this phenomenon, focusing particularly on whether it also occurs *in vivo* and its possible functional importance for peroxisomal targeting. Moreover, systematic analysis in yeast has uncovered many Pex5 cargo proteins that are Pex5 dependent but PTS1 independent (Yifrach et al., 2022).

What is the function of the newly identified interface between Pex5 and Eci1? One possibility is that while the PTS1 serves as an anchor binding domain, the additional cargo-specific binding interfaces enable dynamic interaction with Pex5 that enables additional levels of regulation in accordance with the needs of the cell.

Notably, we demonstrate that both Pex5 and CoA bind Eci1 using overlapping residues in the EBI. Cofactors, such as CoA, are essential for enzymatic reactions inside peroxisomes, yet the mechanism of their import remains unclear. It has been previously suggested that the import of protein–cofactor complexes might enable the coupling of protein transport with cofactor import (Plett et al., 2020). Interestingly, we observed that in most structures, only one Eci1 subunit mediated the interaction with Pex5 whereas the other five subunits did not bind Pex5. This asymmetrical binding of Pex5 to Eci1 could facilitate CoA binding to the remaining subunits, potentially enabling CoA piggybacking into peroxisomes during Eci1 targeting.

Although it is reasonable to assume that a single Pex5 molecule binding to the Eci1 hexamer is sufficient for peroxisomal targeting, we cannot exclude the possibility that, *in vivo*, all six Eci1 subunits interact with Pex5. Such an interaction could prevent CoA binding and Eci1 activity until the hexamer is properly targeted to peroxisomes.

Interestingly, the EBI comprises one of four highly conserved amphipathic α-helices (AH) previously identified in Pex5 (Gaussmann et al., 2021; Skowyra and Rapoport, 2022). Although this helix is evolutionarily conserved, its contribution to cargo targeting has not yet been demonstrated. Here, we demonstrate that the AH3 and AH4 (human numbering AH4 and AH5) has a crucial part in mediating a non-canonical binding of Pex5 with its cargo protein Eci1.

Our findings, together with prior Pex5–cargo structures, suggest the need to reconsider existing paradigms of Pex5-mediated targeting. Further studies are required to elucidate the biological importance of non-canonical Pex5-binding interfaces.

Moving beyond the ‘canonical’ framework that has traditionally guided peroxisomal targeting research – particularly with respect to PTS1-containing proteins – might reveal new peroxisomal cargo proteins that utilize alternative interaction mechanisms. Additionally, this perspective will provide insights into the dynamic nature of Pex5 function, highlighting how it facilitates the targeting of diverse proteins to peroxisomes. The existence of multiple binding interfaces might enable different levels of regulation, including the selective prioritization of certain cargo proteins over others (Rosenthal et al., 2020), aligning with the physiological needs of the cell and organism.

## MATERIALS AND METHODS

### Yeast strains and strain construction

All strains used in this study were derived from the strain yMS4097 or yMS3021. Genetic manipulations were performed on haploid strains using a homologous recombination-based transformation of a suitable PCR product using the lithium-acetate method (Wei Xiao, 2006). For KO strains, the PCR product included the selection cassette of the respective strain, replacing the open reading frame (ORF) of the deleted gene. For over-expression strains, a selection cassette followed by a synthetic promoter was integrated immediately upstream of the ORF of the gene of interest. The correct insertion of the PCR product was verified in all strains.

The primers in this study used for yeast genetic manipulation were designed using the web tool Primers-4-Yeast (Yofe and Schuldiner, 2014). Detailed information on all strains, primers and plasmids used in this study can be found in the Tables S1–S3, respectively.

### Yeast growth media

The synthetic media used in this study contains 6.7 g/l yeast nitrogen base with ammonium sulfate (Conda Pronadisa #1545) and either 2% glucose (SD) or 0.2% oleic acid (Sigma-Aldrich, St Louis, MO, USA, and 0.1% Tween 80 (Sigma-Aldrich) (S-oleate), both supplemented with a complete amino acid mix (oMM composition; Hanscho et al., 2012). When geneticin antibiotic was required, the media contained 0.17 g/l yeast nitrogen base without ammonium sulfate (#1553 Conda Pronadisa, Madrid, Spain) and 1 g/l of monosodium glutamic acid (Sigma-Aldrich, #G1626). Strain selection was performed using a dropout mix (same composition as ‘SD’, but without the specific amino acid for selection) or with antibiotics using the following concentrations: 500 mg/l geneticin (G418; Formedium), or 200 mg/l Nourseothricin (NAT; WERNER BioAgents ‘ClonNat’).

### Microscopy

#### Standard resolution microscopy

Images in Figs 1A,C, 2, and Fig. S1 were obtained using fluorescent microscopy as follows. Yeast strains were grown overnight in an SD-based medium with the appropriate selection in 96-well polystyrene plates and were then transferred to S-oleate for 20 h. The cultures were transferred to glass-bottom 384-well microscope plates (Matrical Bioscience) coated with concanavalin A (Sigma-Aldrich). After 20 min, the wells were washed twice with double-distilled water (DDW) to remove non-adherent cells, leaving a monolayer of cells for imaging. The imaging was performed in DDW. For Figs 1A,C and 2, the images were acquired using the VisiScope Confocal Cell Explorer system, which consists of a Zeiss Yokogawa spinning disc scanning unit (CSU-W1) coupled with an inverted Olympus microscope (IX83; ×60 oil objective; excitation wavelengths of 488 nm for mNeonGreen and 561 nm for mScarlet). Images were captured by a connected PCO-Edge sCMOS camera controlled by VisiView software. For Fig. S1, the images were taken using the Olympus IXplore SpinSR system, which consists of an Olympus IX83 inverted microscope scanning unit (SCU-W1). Cells were imaged using an ×60 air objective and were recorded in two channels – mNeonGreen (excitation wavelength 488 nm) and mCherry (excitation wavelength 561 nm).

All images were manually analyzed using Fiji software, an open-source platform for biological images analysis (Schindelin et al., 2012). For all micrographs, a single, representative focal plane is shown. In each figure, the same confocal settings were used for the imaging of all strains. Similar brightness and contrast settings were used for all panels in each figure (separately), except for Fig. S1 (in which the settings were adjusted independently for each strain for optimal visualization of the tagged protein).

For the colocalization quantification of Eci1 with peroxisomes, a custom macro was implemented in Fiji (Schindelin et al., 2012). Stardist, a plugin for star-convex shape-based object detection, was employed for the segmentation of peroxisomes (Frangi et al., 2018). Cellpose, a deep learning-based tool, was used for segmenting yeast cells (Stringer et al., 2021). Intensity thresholds were applied to isolate relevant regions, and regions of interest (ROIs) were filtered and processed to ensure accurate spatial containment of peroxisomes within yeast cells (Fig. S6). The macro quantifies the Eci1 protein signal intensity both inside and outside peroxisomes, calculating total and mean intensities for each region. Results, including intensity ratios and area measurements, are exported as CSV files alongside corresponding ROI sets for further analysis. The source code for the macro, along with usage instructions, is available in a dedicated GitHub repository (https://github.com/WIS-MICC-CellObservatory/Peroxisome-in-Yeast-cells/). Statistical analysis of the imaging quantification data was done by using GraphPad Prism version 10. In each graph, one experiment with three technical repeats (three different imaging fields) is depicted. Each technical repeat is comprised of >100 cells; all cells in each repetition were used for the quantification.

#### SORA high-resolution imaging

A strain containing a *PEX11* deletion (Fig. 1B) was grown in S-oleate and prepared for imaging following the ‘Standard resolution microscopy’ method described earlier. The images were taken using the Olympus IXplore SpinSR system, which consists of an Olympus IX83 inverted microscope scanning unit (SCU-W1) and a high-resolution spinning disc module (Yokogawa CSU-W1 SORA confocal scanner with double microlenses and 50 µm pinholes), operated by ScanR software. Cells were imaged using an ×60 oil lens (NA 1.42) and a Hamamatsu ORCA-Flash 4.0 camera. Images were recorded in two channels, mNeonGreen (excitation wavelength 488 nm) and mScarlet (excitation wavelength 561 nm). Cells were imaged using *Z*-stacks (19 stacks, 0.2 µm distance between the stacks); for all micrographs, a single, representative focal plane is shown. Images were manually analyzed using FIJI software (Schindelin et al., 2012), and the brightness and contrast settings used were for optimal visualization of the phenotype.

### Eci1 and Pex5 plasmid construction

All cloning reactions were performed by the restriction-free (RF) method (Unger et al., 2010). Full-length *PEX5* (1–612) from *Saccharomyces cerevisiae* was cloned into the expression vector pET28-bdSumo (Zahradník et al., 2019), using RF cloning (Unger et al., 2010) yielding Pex5 with a cleavable N-terminal His-Sumo fusion. *S. cerevisiae ECI1* was cloned into the first open-reading frame of the expression vector pACYCDuet-1 (Novagen), which includes an N-terminal Flag-tag followed by a TEV cleavage site. The Eci1ΔPTS1 construct was generated by deleting the last three amino acids (^278^HRL^280^) of the protein. This reaction was carried out using transfer-PCR (TPCR) (Erijman et al., 2011). The primers used for cloning *ECI1* WT and *PEX5*, and for generating *ECI1*ΔPTS1 are listed in Table S2.

### Eci1 and Pex5 protein co-expression

Eci1 (in *pACYCDut-FLAG-ECI1*), Eci1ΔPTS1 (in *pACYCDut-FLAG-*Δ*ECI1*) and Pex5 (in *pET28-bdSumo-PEX5*) were expressed individually or co-expressed (Pex5 and Eci1, and Pex5 and Eci1ΔPTS1) in *E. coli* BL21(DE3). Expression was performed in LB medium (LB Broth; Condalab, cat. no 1231.00) supplemented with the appropriate antibiotics (kanamycin and/or chloramphenicol; Duchefa Biochemie, cat. no. K0126, and Sigma, cat. no. C0378, respectively). For small-scale and large-scale expression, 10 ml and 5 l cultures were used, respectively. Expression was induced with 200 μM IPTG (Inalco Pharmaceuticals, cat. no. 1758-1400), followed by shaking at 15°C for ∼18 h. Cell pellets were prepared by centrifugation at 5000 ***g*** for 10 min were stored at −20°C before further processing.

### Small-scale Eci1 and Pex5 protein pulldown

Cell pellets (derived from 10 ml culture) were lysed by sonication in Tris-buffered saline (TBS) buffer supplemented with 1 mM phenylmethylsulfonyl fluoride (PMSF) and 1 μl/ml of protease inhibitor cocktail (Set IV, EMD Chemicals, Inc). Protein pulldown experiments were performed using Ni-resin (Adar Biotech), following the manufacturers' recommendations. Western blot analysis was performed using THE DYKDDDDK Tag Antibody (HRP-conjugated; A01428, GenScript, 1:1000 dilution) and monoclonal HRP-conjugated anti-polyhistidine-peroxidase (1:3000 dilution; A7058, Sigma). The proteins were analyzed on 4-20% SurePAGE precast gels (M00657, GeneScript). Images of the complete membranes of the immunoblots are shown in Fig. S2A.

### Purification of Pex5

A cell pellet from a 5 l culture was resuspended in lysis buffer (50 mM Tris-HCl pH 8, 0.5 M NaCl) supplemented with 200 KU/100 ml lysozyme (Roche lysozyme from hen egg white; cat. no. 10837059001), 20 μg/ml DNaseI (from bovine pancreas, Type IV, cat. no. D5025-750KU), 1 mM MgCl_2_, 1 mM PMSF and protease inhibitor cocktail (set III, EDTA-free, Calbiochem, cat. no. 539134). The cells were lysed using a cooled cell disrupter (Constant Systems). The lysate was clarified by centrifugation (50,000 rpm for 30 min; Beckman Coulter Rotor Type 70 Ti ) and loaded onto a Ni-affinity column (Cytiva). The column was washed with lysis buffer containing 50 mM imidazole, and His–Sumo–Pex5 was eluted using 500 mM imidazole. The eluate was dialyzed overnight at 4°C against PBS buffer containing 1 mM DTT and 250 mM sucrose, supplemented with His-tagged *bdSumo* protease for cleavage. The cleavage mixture was subjected to reverse IMAC, where the His-tagged protease and His–Sumo fusion were retained on the Ni column, and Pex5 eluted in the flow-through (FT). The FT containing Pex5 was applied to a size exclusion column (HiLoad_16/60 Superdex 200, Cytiva) equilibrated with PBS. Pex5 migrated as a single peak, which was pooled, aliquoted, frozen in liquid nitrogen and stored at −80°C.

### Purification of Eci1 and Eci1ΔPTS1

The cell pellet from a 5 l culture was resuspended in lysis buffer (50 mM HEPES, pH 7.5, 10% glycerol, 0.3 M NaCl, 1 mM BME) supplemented with 200 KU/100 ml lysozyme, 20 μg/ml DNase, 1 mM MgCl_2_, 1 mM PMSF and a protease inhibitor cocktail. The cells were lysed using a cooled cell disruptor (Constant Systems). The lysate was clarified by centrifugation (50,000 rpm for 30 min; Beckman Coulter Rotor Type 70 Ti ) and incubated with 1 ml of FLAG beads (GenScrip Biotech) for 1 h at 4°C. After removing unbound material and washing the beads with lysis buffer, Eci1 was eluted using 5 ml of FLAG peptide at 100 μg/ml (GenScrip Biotech). The eluted Eci1 was injected into a size exclusion column (HiLoad 16/60 Superdex 200, Cytiva) equilibrated with PBS. Eci1 or Eci1ΔPTS1 eluted as a single peak, which was pooled, aliquoted, frozen in liquid nitrogen and stored at −80°C.

### Co-purification of Pex5–Eci1 complex

A cell pellet from a 5 l culture was resuspended in lysis buffer (50 mM Tris-HCl pH 8, 0.5 M NaCl) supplemented with 200 KU/100 ml lysozyme, 20 µg/ml DNase, 1 mM MgCl_2_, 1 mM PMSF and protease inhibitor cocktail. The resuspended cells were lysed by a cooled cell disrupter (Constant Systems). The clarified lysate was incubated with 5 ml washed Ni beads (Adar Biotech) for 1 h at 4°C. After removing the unbound supernatant, the beads were washed four times with 50 ml lysis buffer. The Pex5–Eci1 complex was eluted from the washed beads by incubation with 5 ml Sumo-cleavage buffer (40 mM Tris-HCl pH 7.5, 250 mM NaCl, 250 mM Sucrose, 2 mM MgCl_2_ supplemented with 0.2 mg *bdSumo* protease) for 2 h at room temperature (RT). The supernatant fraction, containing the cleaved Pex5–Eci1 complex, was removed and applied to a size exclusion column (Superdex 200 10/300 GL, Cytiva) equilibrated with PBS. The peak corresponding to the Pex5–Eci1 complex, migrating at 11 ml, was pooled and concentrated (Fig. S3A). The presence of both Pex5 and Eci1 in the complex peak was verified by SDS-PAGE stained with Coomassie Brilliant Blue (Fig. S3B). The pure complex was flash-frozen in aliquots using liquid nitrogen and stored at −80°C.

### Surface plasmon resonance analysis of protein interactions

Pex5, Eci1 and Eci1ΔPTS1 were separately expressed and purified as described above (Fig. S2B). The purified proteins were subjected to dynamic light scattering (DLS) analysis using a DynoPro NanoStar II instrument (Wyatt). The DLS data confirmed that all three proteins were monodisperse and free of aggregates (Fig. S2B). To investigate protein–protein interactions, Pex5 was immobilized onto two separate channels of a CM5 Series S Biacore sensor chip (Cytiva) using amine coupling, with final immobilization levels of 450 and 480 RU, respectively. Eci1 WT and Eci1ΔPTS1 concentrations were accurately determined using a spectrophotometer, accounting for buffer and baseline contributions. Serial dilutions of both proteins (1.95 nM to 500 nM) were prepared, and each was injected separately as analytes over the respective immobilized Pex5 (two separate channels). Notably, the dissociation of the Pex5–Eci1 complex required NaOH treatment. However, even mild NaOH exposure resulted in the inactivation of immobilized Pex5, limiting chip reusability. To minimize these issues, the binding of Eci1 WT and Eci1ΔPTS1 to immobilized Pex5 was assessed in single-cycle kinetics mode without dissociation of the bound proteins. Eci1 WT and Eci1ΔPTS1 were injected for 120 s, followed by a 900-second dissociation phase at a flow rate of 30 µl/min at 25°C. Sensorgrams were analyzed using the Biacore Insight Evaluation Software (Cytiva, version 4.0.8.19879). The data for Pex5-Eci1 WT were best fitted to a bivalent analyte model, yielding dissociation constants of *K*_D1_=150 nM and *K*_D2_=18 µM. In contrast, the sensorgrams for Eci1ΔPTS1 could not be fitted to either a bivalent or 1:1 binding model, indicating weak and complex binding dynamics.

### Sample preparation for EM

A total of 2.5 µl of Pex5–Eci1 complex, at a concentration of 2 mg/ml was transferred to glow-discharged Au-Flat 1.2/1.3 300 mesh grids (Protochips). The sample was blotted for 3 s at 4°C and 100% humidity, then plunge-frozen in liquid ethane, and cooled by liquid nitrogen using a Vitrobot plunger (Thermo Fisher Scientific).

### Cryo-EM image acquisition

Cryo-EM data were collected using a Titan Krios G3i transmission electron microscope (Thermo Fisher Scientific) operated at 300 kV. Movies were recorded on a K3 direct detector (Gatan) installed behind a BioQuantum energy filter (Gatan), using a slit width of 15 eV. The movies were recorded in counting mode at a nominal magnification of 105,000x, corresponding to a physical pixel size of 0.842 Å. The dose rate was set to 19.4 e-/pixel/sec, with a total exposure time of 1.6 s, resulting in an accumulated dose of 45.5 e^−^/Å^2^. Each movie was split into 47 frames with an exposure time of 0.034 s per frame. The nominal defocus range was set between −1.0 and −1.5 μm, though the actual defocus range was larger. Imaging was undertaken using an automated low-dose procedure implemented in SerialEM v3.9-beta7 (Mastronarde, 2005). A single image was collected from the center of each hole, using image shift to navigate within hole arrays containing up to 5×5 holes, while stage shift was used to move between arrays. The beam tilt was adjusted to achieve coma-free alignment when applying image shift.

### Cryo-EM image processing

Image processing was performed using CryoSPARC software v3.0.1 (Punjani et al., 2017). A total of 5577 acquired movies were subjected to patch motion correction, followed by patch CTF estimation (Fig. S3D). Of these, 4727 micrographs, with a CTF fit resolution better than 5 Å and a relative ice thickness lower than 1.2 were selected for further processing. Initial particle picking was done using the ‘Blob Picker’ job on a subset of micrographs. Extracted particles were classified in 2D, and selected class averages showing the Eci1 hexamer features were used as templates for automated particle picking from all selected micrographs, resulting in 2,170,575 picked particles. The particles were extracted, binned 4×4 (64-pixel box size, 3.37 Å/pixel), and cleaned by several rounds of 2D classification, resulting in 1,083,831 particles. These particles were re-extracted and binned 2×2 (200-pixel box size, 1.68 Å/pixel) for *ab initio­* 3D reconstruction using a single class, followed by non-uniform refinement. Both *ab initio* and non-uniform refinements revealed a single Pex5 monomer bound to the Eci1 hexamer. Using 3D variability analysis (Punjani and Fleet, 2021) (eight modes, 10 Å low-pass filter) with a spherical mask of 160 Å diameter imposed, followed by classification into 20 3D classes, a minor population of Eci1 hexamers bound to multiple Pex5 molecules was resolved (Fig. S3B). In these subsets, bound Pex5 molecules were refined to a limited resolution due to conformational variability. Blurred density from multiply bound Pex5 molecules can also be observed in 2D class averages (Fig. S3B). To better resolve Pex5 conformational variability and its interface with Eci1, we focused on the complexes of 6 Eci1:1 Pex5, which represented the vast majority of the particles. 3D variability analysis was performed with an imposed real-space solvent mask generated by the non-uniform refinement above (3 modes, 10 Å low-pass filter), followed by classification into 10 3D classes. Particles from all classes were re-extracted without binning into separate datasets and subjected to non-uniform refinement. Subsequently, local refinement was performed using a soft-edged ellipsoid mask around Pex5 (including the Pex5–Eci1 interface) in initial iterations and a ‘dynamic’ solvent mask in final iterations once a resolution better than 5 Å was reached. This analysis clearly shows conformational variability in Pex5 binding (Fig. S3C). The best-resolved class (Fig. S3D, 117,945 particles) reached 2.7 Å and 2.9 Å resolution through non-uniform and local refinement, respectively. This class was used for downstream atomic coordinate modeling. The rest of the classes showed lower rigidity at the Pex5 area and Eci1 interface.

Particle images of smaller complexes were identified and processed; however, their reconstructions were limited to low resolution, which made interpretation unreliable. Based on size and shape, these particles can be attributed to Pex5 or Eci1 subunits. 3D visualization was performed using UCSF Chimera (Pettersen et al., 2004).

### Model building of Pex5–Eci1 complex

Model building of the yeast Pex5–Eci1 complex involved docking the known Eci1 hexamer crystal structure and a predicted model of full-length Pex5, obtained using the AlphaFold2 software (Senior et al., 2020; Jumper et al., 2021), onto the cryo-EM maps. The N-terminal segment of Pex5 (up to Y301) was predicted to be unstructured, with low accuracy, as indicated by correspondingly low model prediction on the local distance different test (pLDDT) which scored below 50. Owing to this uncertainty, the N-terminal segment was truncated in the model. The predicted model of Pex5 (A302–F612) obtained from AlphaFold2 and the known structure of the Eci1 hexamer from *S. cerevisiae* (PDB entry 1PJH; Mursula et al., 2004) were used as structural templates for docking into the cryo-EM maps. Docking was performed using the Dock-in-Map program within PHENIX (Adams et al., 2010). Specifically, the Eci1 hexamer was docked into the Pex5–Eci1 complex map, and the Eci1–Pex5 complex into the Pex5 local map to obtain the final structural model.

This integration allows for the reconciliation of computational predictions with experimental data, providing a comprehensive and accurate depiction of the Pex5–Eci1 complex structure. All steps of atomic refinements were performed using the Real-space refinement in PHENIX (Klaholz, 2019). The Eci1 hexamer model was built into the Pex5–Eci1 complex map, and the Eci1–Pex5 model into the Pex5 local map using the COOT program (Emsley and Cowtan, 2004). Both models were evaluated with the MolProbity program (Chen et al., 2010).

The cryo-EM map of the Eci1 hexamer reveals electron density for I5–Q270, E4–Q270, I5–Q270, E4–Q270, and I5–Q270 in five subunits, and L4–L280 for the Eci1 subunit that binds Pex5. This subunit contains a unique segment at its C-terminal end (^271^LGSKQRKHRL^280^), which is not observed in the other five subunits.

The Pex5 local refinement map of the Pex5–Eci1 complex containing the full-length Pex5 does not show electron density for the NTD of Pex5 (up to E275) (Fig. S4C). This suggests that the NTD of Pex5 is likely flexible or dynamic within the complex, making it challenging to visualize using cryo-EM. The Pex5 structure (L276–F612) includes the TPR domain (N314-S553), which is consisted of seven TPR repeats, each composed of 34 residues (α1–α2, α3–α4, α5–α6, α7–α8, α9–α10, α11–α12, and α13–α14) (Fig. S4C). This structure also features a PTS1–cargo binding region, which is a well-characterized component of the Pex5 receptor TPR domain.

Details of the refinement statistics of the Eci1–Pex5 structure are described in Table S4. Three-dimensional visualization and analyses were conducted using UCSF Chimera (Pettersen et al., 2004) and PyMol (‘The PyMOL Molecular Graphics System’, Version 2.0 Schrödinger; http://www.pymol.org/pymol). The coordinates for the Eci1 hexamer and the Eci1 (subunit)–Pex5 complex have been deposited in the Protein Data Bank under the PDB 9FGZ and PDB 9FH0, respectively. Their corresponding maps are available in the EMDataResource as EMD-50434 and EMD-50435.

## Supplementary Material



10.1242/joces.263890_sup1 Supplementary information

Table S1. S. cerevisiae and E. coli strains used in this study. A table summarizing all strains of S. cerevisiae and E. coli used in the experiments of this study.

Table S2. Primers used in this study. A table summarizing all primers used in the experiments of this study. All primers up to 9824 (including) were designed using the online tool “Primers-4-Yeast” (Yofe and Schuldiner, 2014).

Table S3. Plasmids used in this study. A table summarizing all plasmids used in the experiments of this study. Plasmids based on the backbones pFA6, pYM, pKL, and SWAT were used in S. cerevisiae; Plasmids based on the backbones pET and pACYDuet were used for expression in E. coli.

Table S4. Cryo-EM data collection and refinement statistics of Pex5-Eci1. This table summarized the Cryo-EM data for determining the structure of the Pex5-Eci1 complex.
