# Supplementary material for: A cryo-electron microscopy structure of yeast Pex5 in complex with a cargo uncovers a novel binding interface
Source: J Cell Sci. 2025 Jun 26;138(12):jcs263890. doi: 10.1242/jcs.263890 (PMC12273641; doi:10.1242/jcs.263890)
Supplement: Supplementary information [file joces-138-263890-s1.pdf]

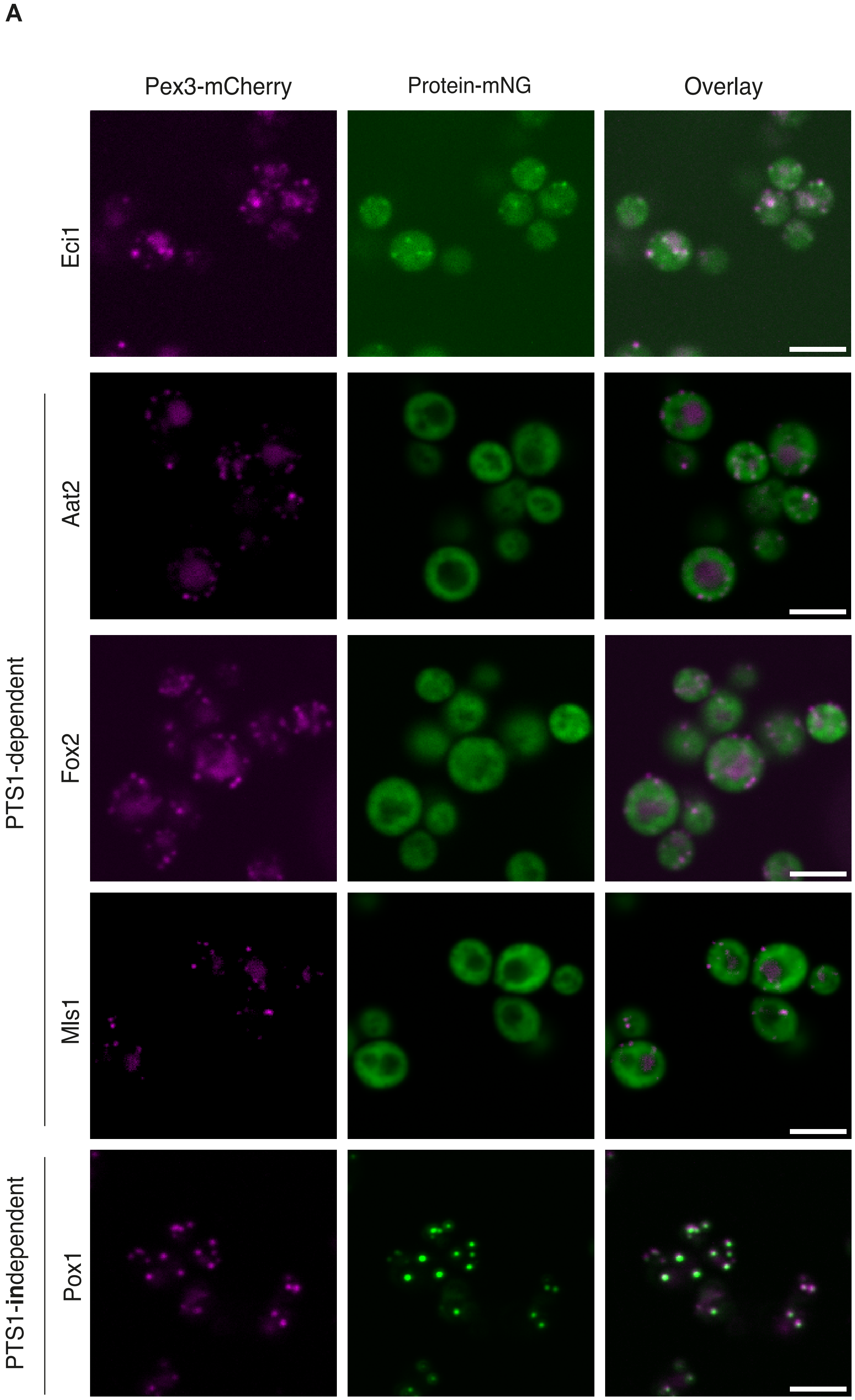
A
Pex3-mCherry
Protein-mNG
Overlay
Eci1
PTS1-dependent
Aat2
Fox2
Mls1
PTS1-independent
Pox1

B

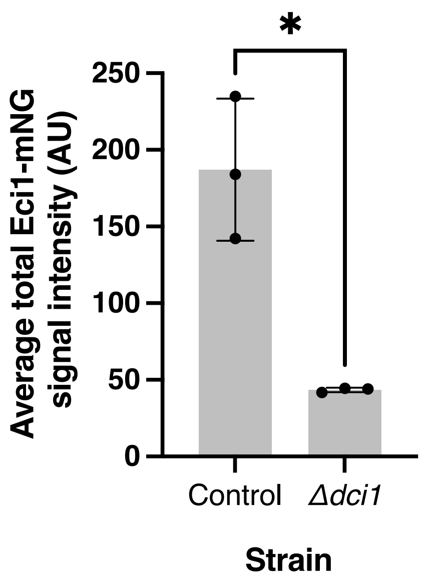
*
250
200
150
100
50
0
Average total Eci1-mNG signal intensity (AU)
Control
Δdci1
Strain

C

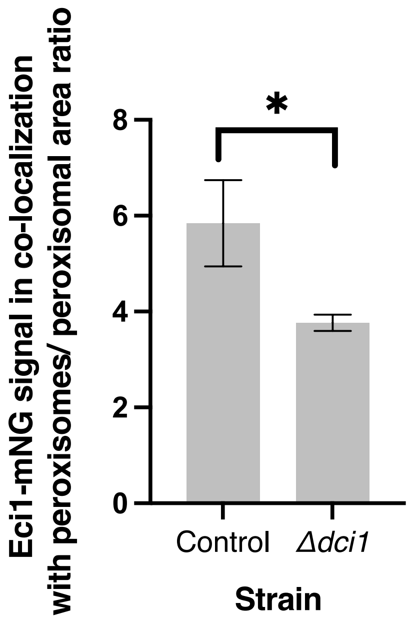
*
8
6
4
2
0
Eci1-mNG signal in co-localization with peroxisomes/ peroxisomal area ratio
Control
Δdci1
Strain

D

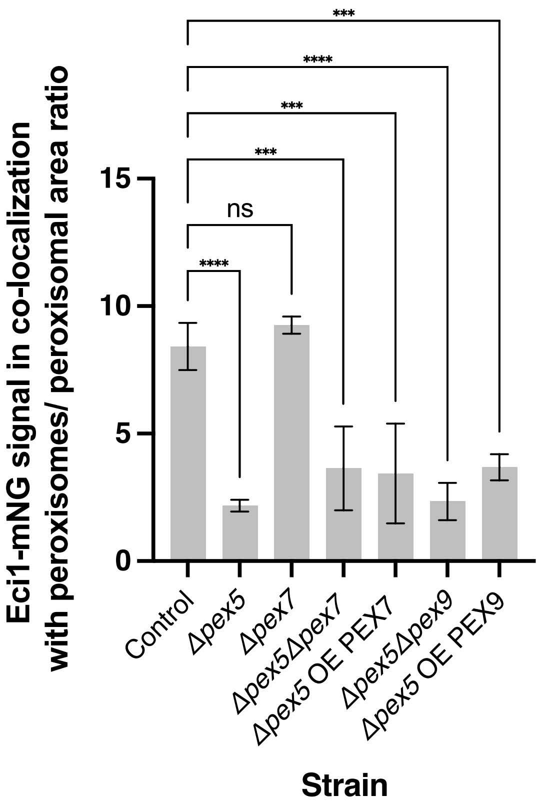
***
****
***
***
ns
****
15
10
5
0
Eci1-mNG signal in co-localization with peroxisomes/ peroxisomal area ratio
Control
Δpex5
Δpex7
Δpex5Δpex7
Δpex5 OE PEX7
Δpex5Δpex9
Δpex5 OE PEX9
Strain

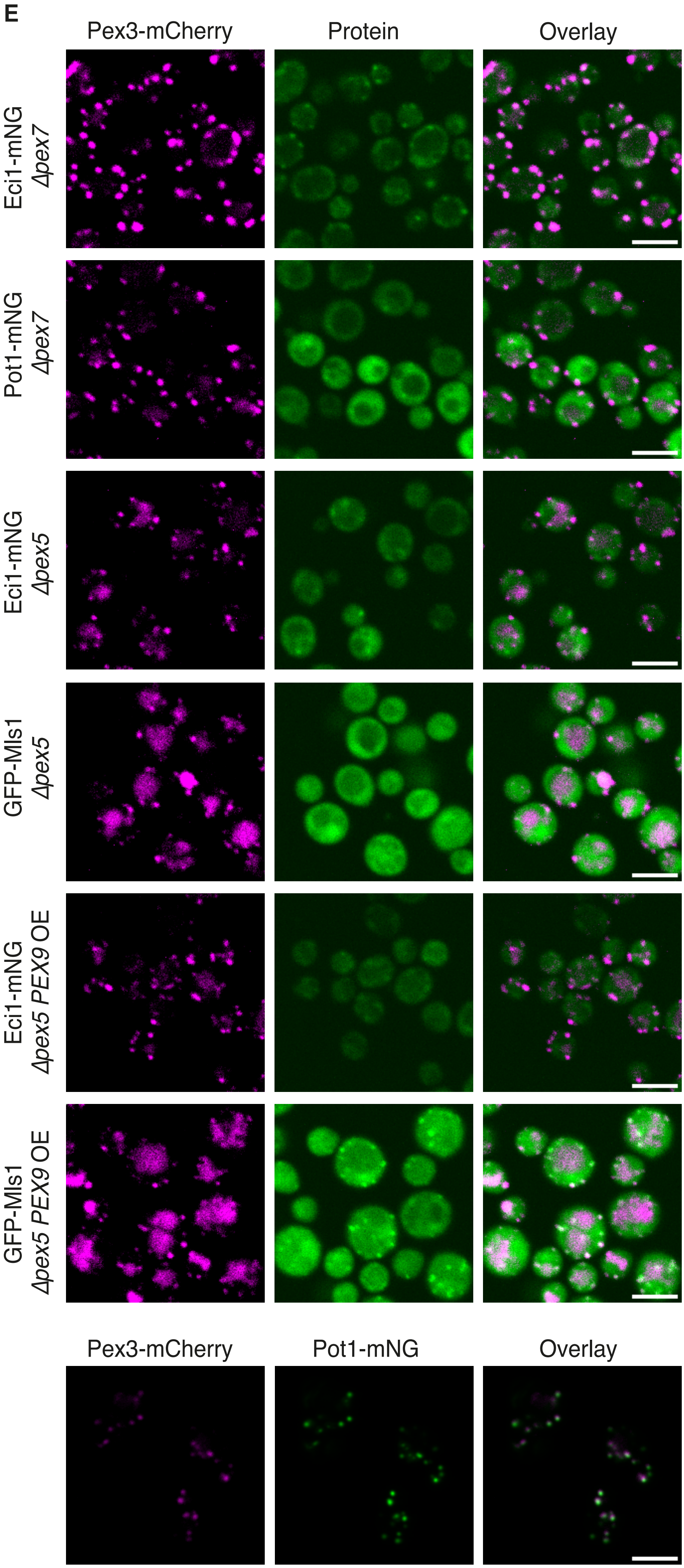

Journal of Cell Science • Supplementary information

Fig. S1. Pex5 is the sole cargo factor mediating Eci1-mNG targeting to peroxisomes.

A) PTS1-dependent peroxisomal cargo proteins lose their peroxisomal localization upon masking their C terminus. Fluorescent microscopy images of peroxisomal proteins C' tagged with mNG. Eci1-mNG maintains co-localization with the peroxisomal marker (Pex3-mCherry) suggesting an additional, PTS1-independent, mode of targeting. Proteins completely dependent on their PTS1 are no longer targeted to peroxisomes upon masking their PTS1 (i.e., Aat2, Fox2, and Mls1). Pox1, on the other hand, is targeted in a PTS1-independent manner, and hence, colocalizes with peroxisomes even when a fluorophore is masking its C terminus. Scale: 5 μm. B) Quantification of average total Eci1-mNG signal (AU- arbitrary units). C) and D) show the quantification of the Eci1-mNG signal in co-localization with peroxisomes divided by the peroxisomal area ratio (as an approximation for the total area of the peroxisomes in all cells combined). Data was analyzed using a Welch t-test (B) or an unpaired t-test (C) or ANOVA with Dunnett's correction for multiple comparisons (D). Each graph represents one experiment with three technical repeats (n=3) in each strain (**p-value<0.01, ***p-value<0.001, ****p-value<0.0001).

E) Fluorescent microscopy images of canonical PTS1 and PTS2 cargo proteins (Mls1 and Pot1, respectively). In the Δ*pex7* background, Eci1 co-localizes with peroxisomes (Pex3-mScarlet); Pot1, conversely, is dependent on Pex7 for its peroxisomal targeting. In Δ*pex5* background, Eci1 is mislocalized to the cytosol. Mls1- a cargo of Pex5 mostly targeted by Pex9-is cytosolic. When over-expressing *PEX9*, Mls1 is co-localized with peroxisomes, while Eci1 is not. Pot1, a canonical PTS2 cargo, is localized to peroxisomes when C' tagged with mNG.

A

anti-FLAG (Eci1) - full membrane, long exposure time

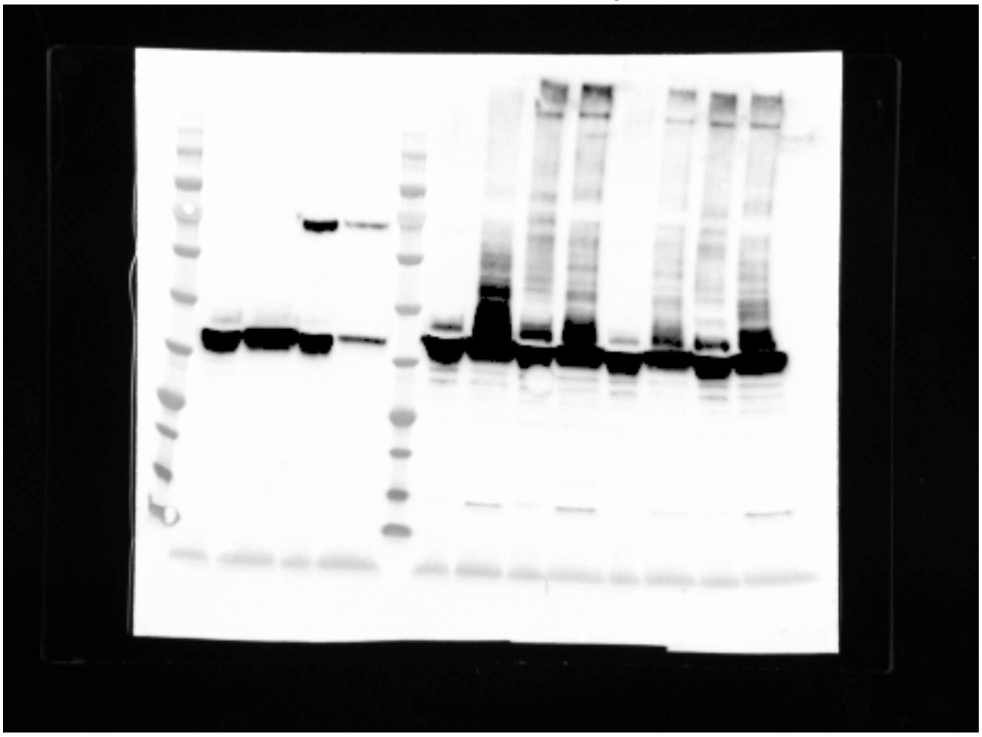

anti-FLAG (Eci1) - full memrabe, short exposure time

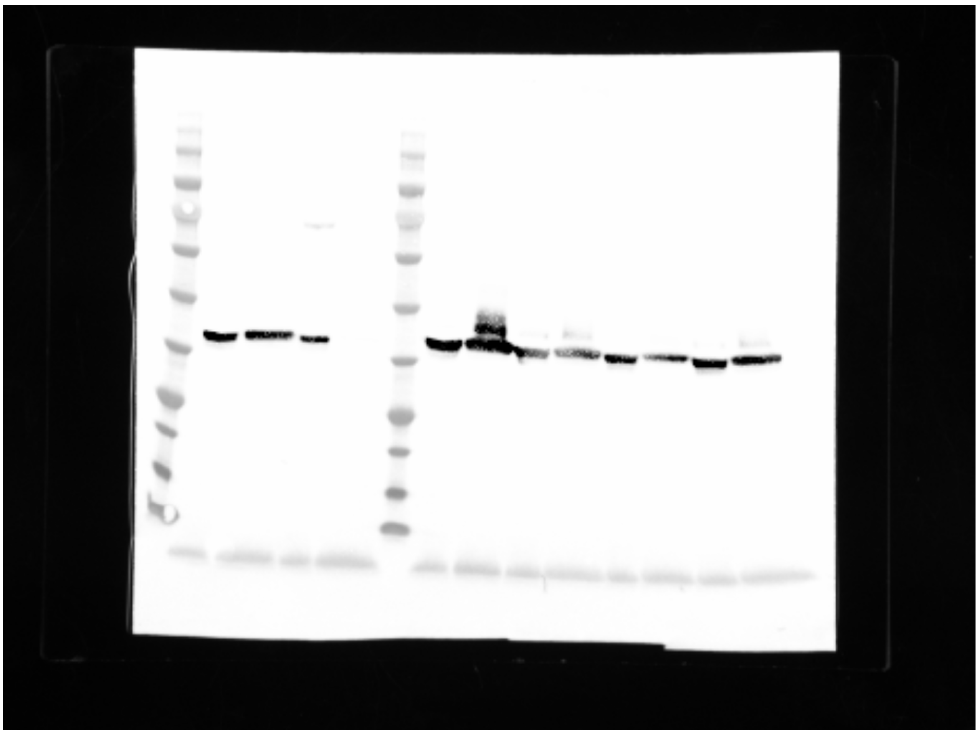

Journal of Cell Science • Supplementary information

anti-His (Pex5)- full membrane

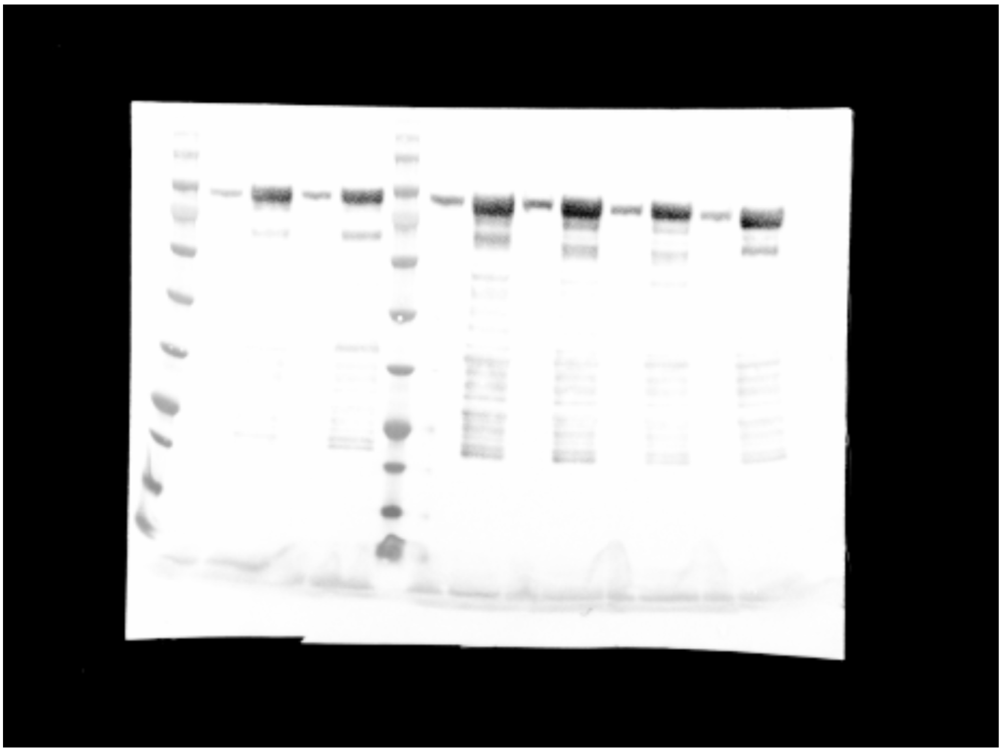

B

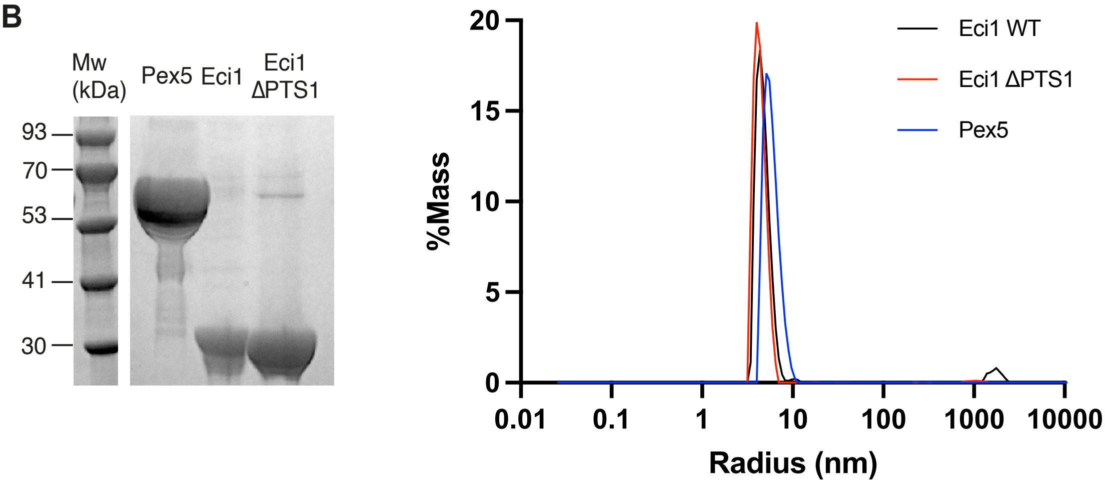

C

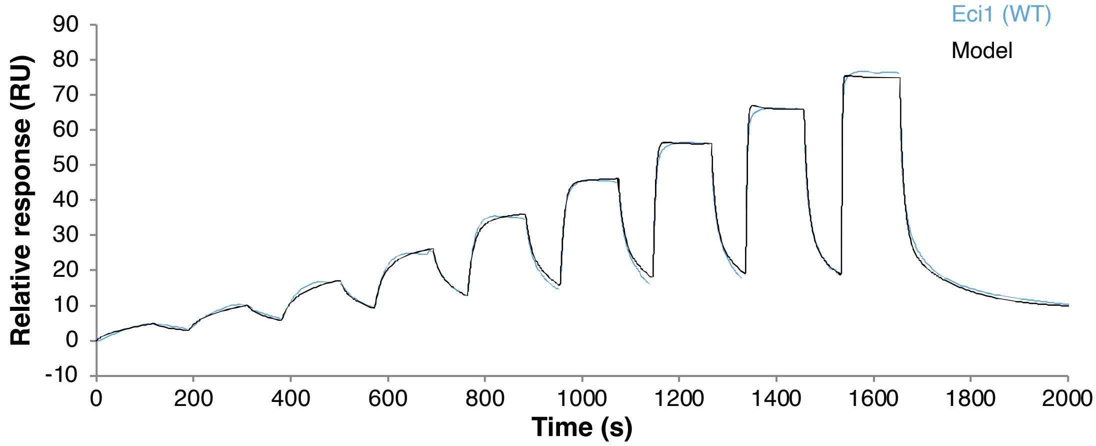

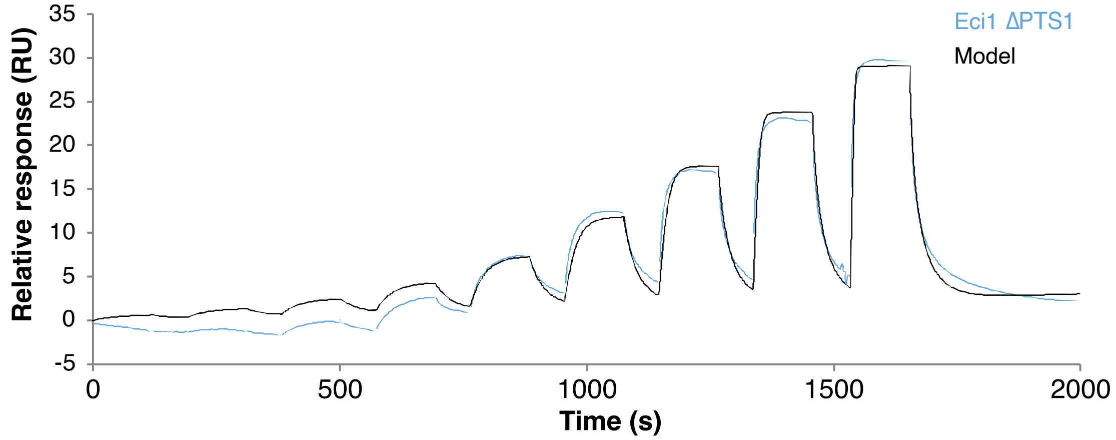

Fig. S2. Eci1 directly binds Pex5 *in vitro*. A) Raw images of the immunoblots in Figure 3A, where the left four lanes are shown. B) Sample preparation for Biacore analysis, comprised of an SDS-PAGE stained with Coomassie Blue depicting the individually expressed proteins Pex5, Eci1 WT, and Eci1 ΔPTS1 (left). On the right, a Dynamic Light Scattering (DLS) of the purified proteins, showing all three purified protein samples do not contain aggregates. C) Evaluation of Eci1-Pex5 interaction using SPR. Binding was assessed in single-cycle kinetics mode without dissociation of the bound proteins using growing concentrations of each of the analytes (1.95nM, 3.91nM, 7.81nM, 15.63nM, 31.25nM, 62.5nM, 125nM, 250nM, 500nM). Applying a bivalent mode of interaction, the Eci1 WT-Pex5 interaction (upper graph) shows a high degree of fit to the model. The Eci1ΔPTS1-Pex5 interaction (bottom graph) does not fit a 1:1 model of interaction.

A

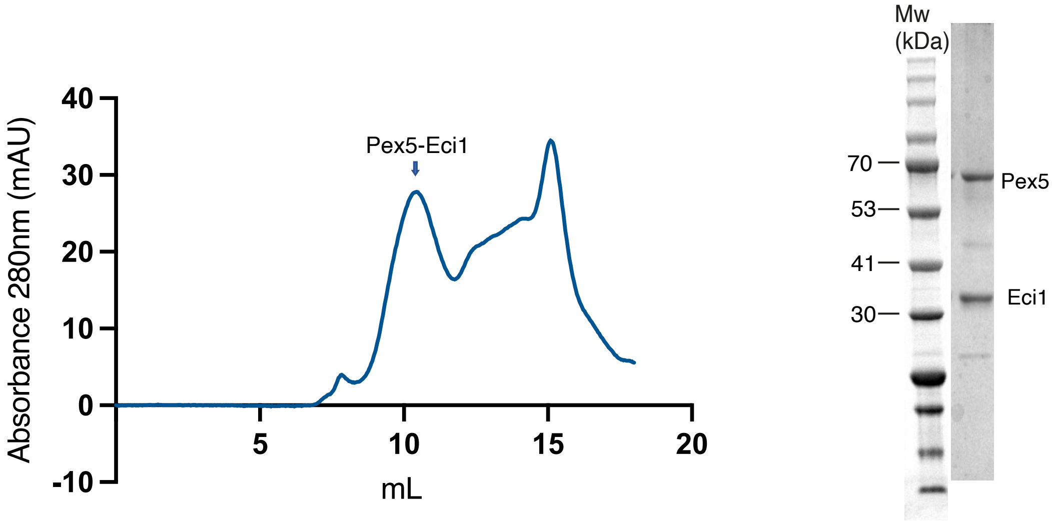

B

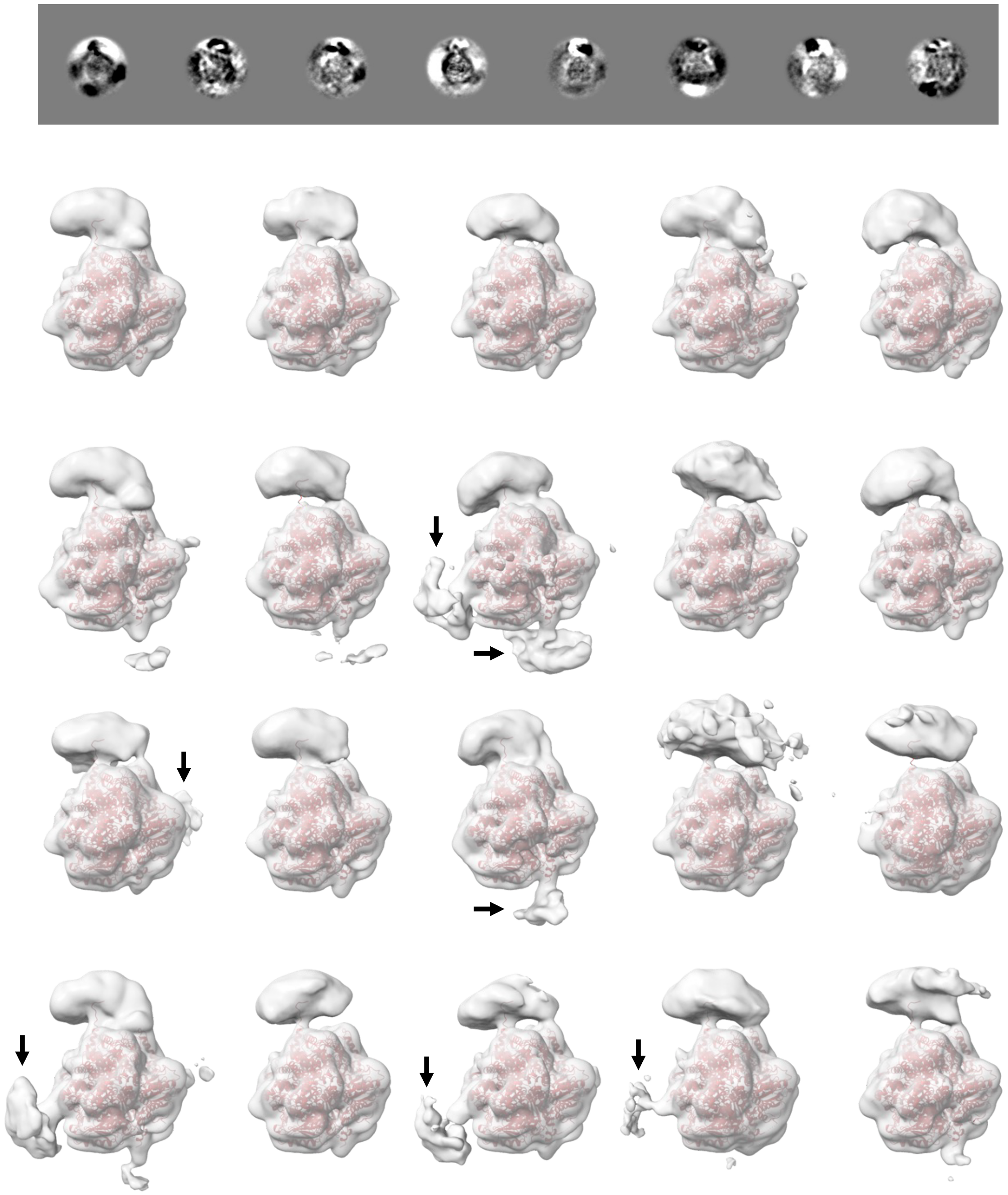

Journal of Cell Science • Supplementary information

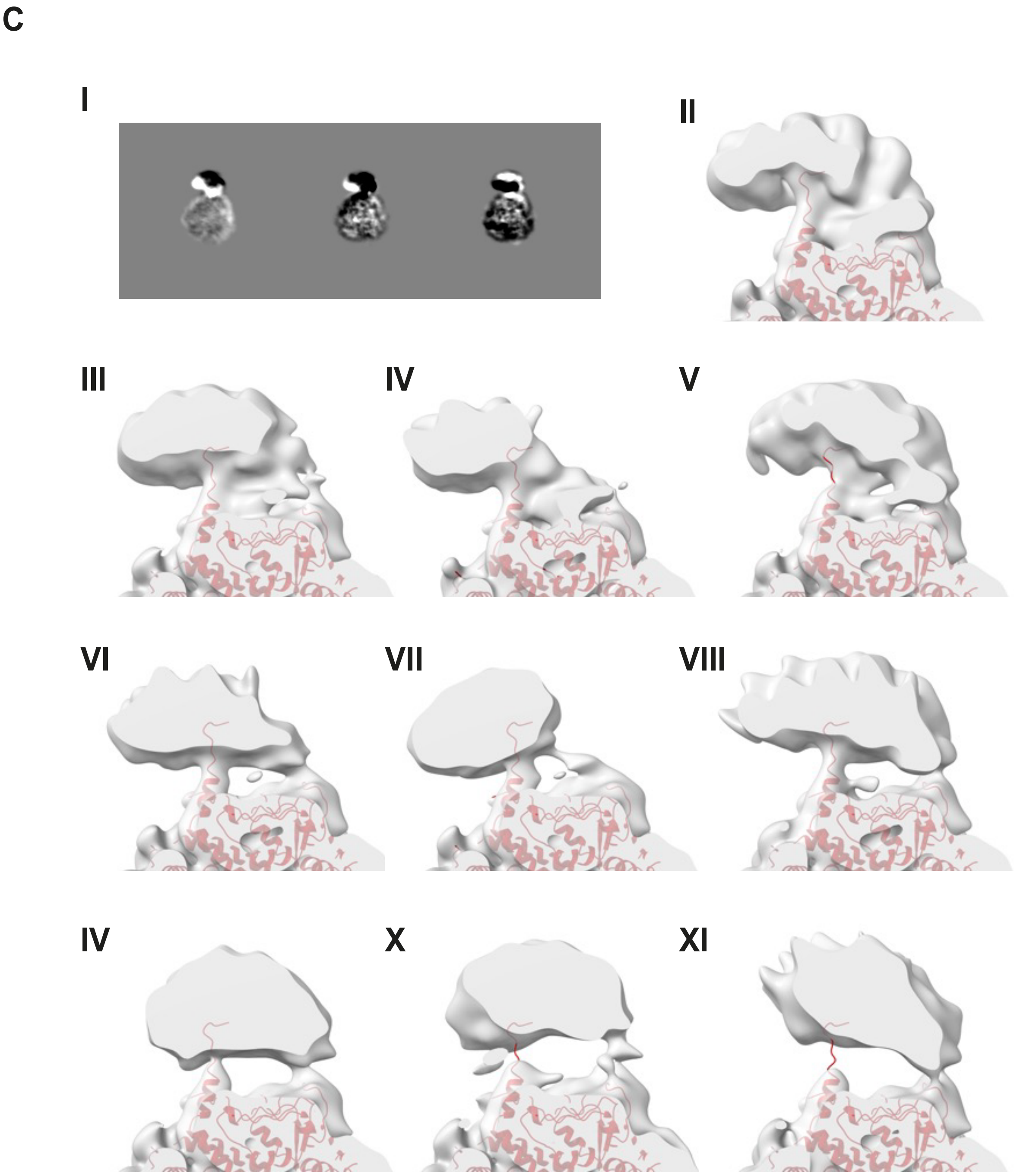

Journal of Cell Science • Supplementary information

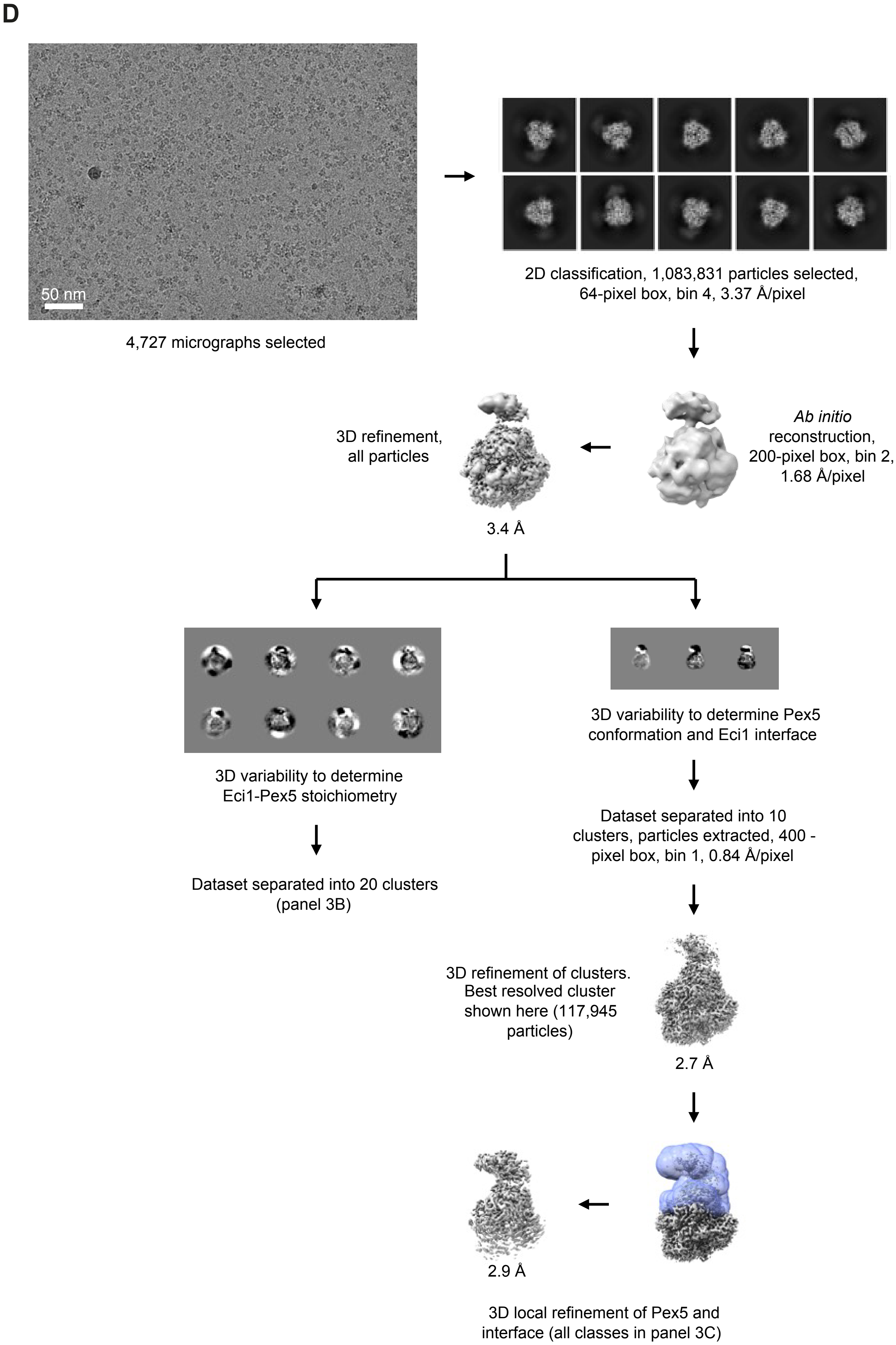

Journal of Cell Science • Supplementary information

E

I

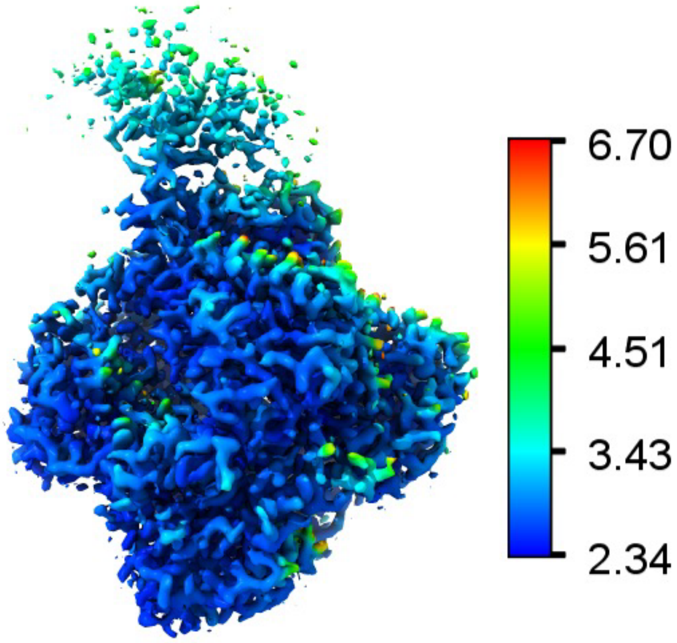
6.70
5.61
4.51
3.43
2.34

II

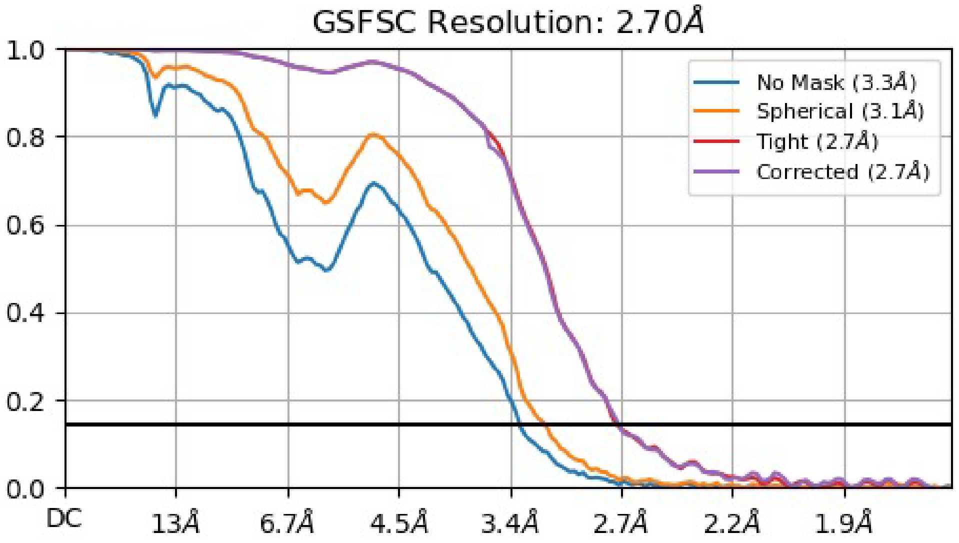
GSFSC Resolution: 2.70Å
1.0
0.8
0.6
0.4
0.2
0.0
DC
13Å
6.7Å
4.5Å
3.4Å
2.7Å
2.2Å
1.9Å
No Mask (3.3Å)
Spherical (3.1Å)
Tight (2.7Å)
Corrected (2.7Å)

III

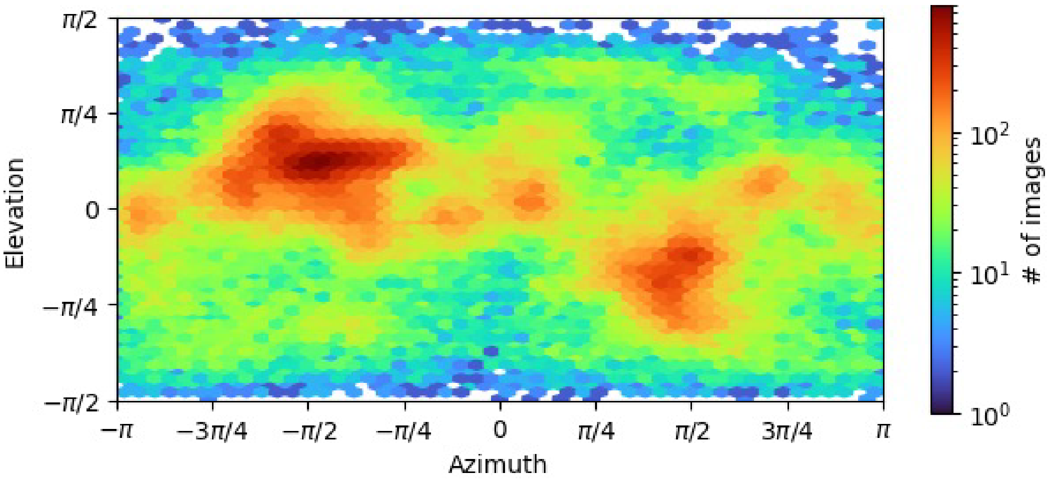
π/2
π/4
0
−π/4
−π/2
Elevation
−π
−3π/4
−π/2
−π/4
0
π/4
π/2
3π/4
π
Azimuth
10^2
10^1
10^0
# of images

IV

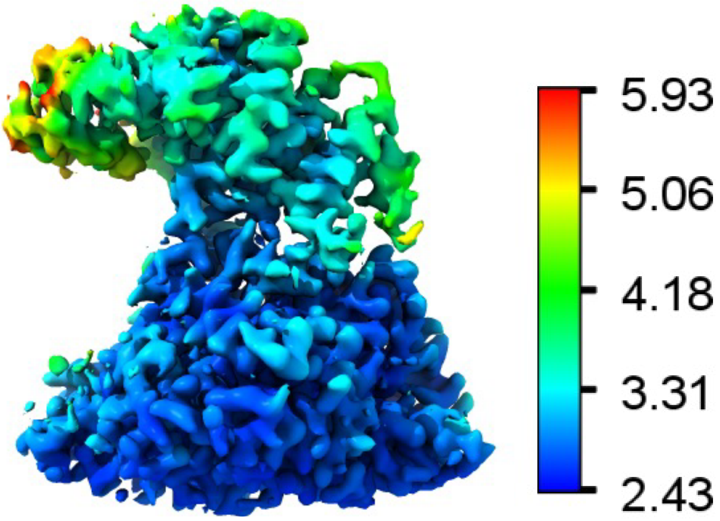

V

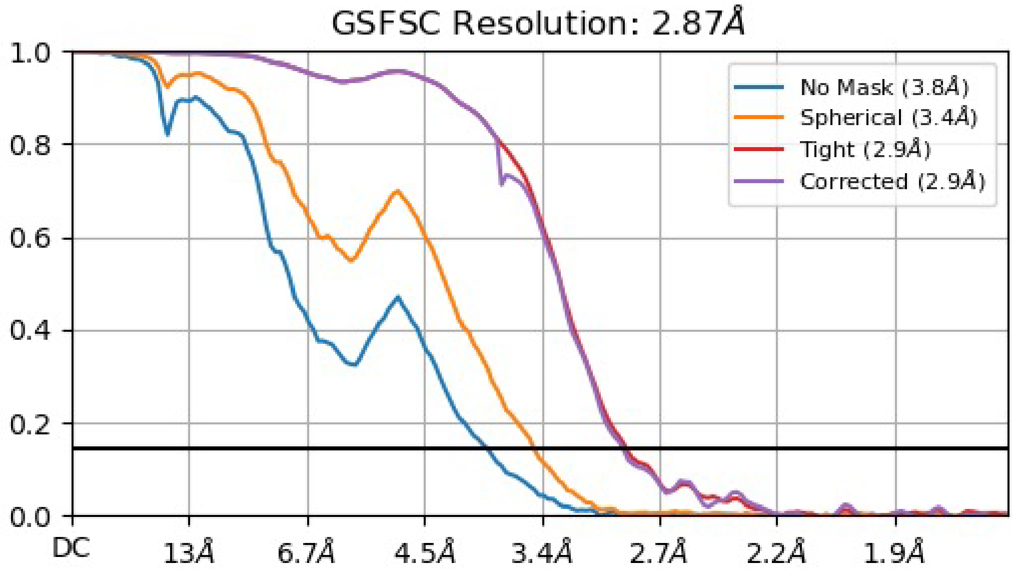

VI

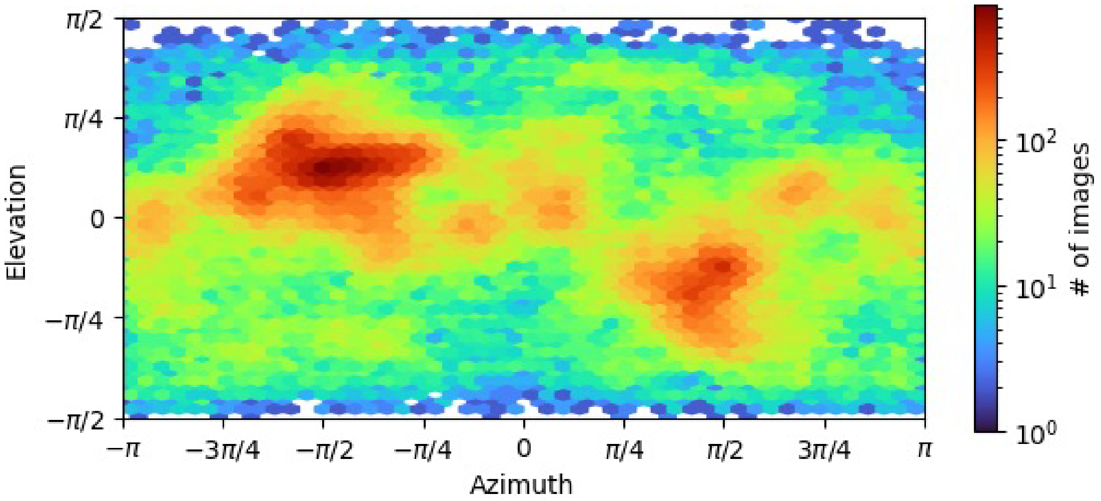

Journal of Cell Science • Supplementary information

Fig. S3. Isolation of the Pex5-Eci1 complex and Cryo-EM analysis. A) SEC profile of the co-purification of the Pex5-Eci1 complex, depicting the peak taken for structural analysis. On the right, an SDS-PAGE (stained with Coomassie Blue) of the peak isolated. B) Analysis of Pex5-Eci1 stoichiometry in the cryo-EM dataset. Eight eigenvectors (modes) were calculated using 3D viability analysis, applying a spherical mask to include the Eci1 hexamer and surrounding Pex5 subunits (I). Contrast at the periphery of the mask indicates variability in Pex5 stoichiometry and/or conformation. (II) The image dataset was separated into 20 clusters based on the above 8 eigenvectors, and a single 3D map was calculated for each cluster. Eci1 coordinates were docked in for reference (in red). The majority of Eci1 hexamers are bound by a single Pex5, while in some clusters, additional Pex5 molecules are observed (indicated by arrows), all adopting a similar orientation relative to the hexamer. C) Pex5 conformational variability. (I) Three eigenvectors (modes) were calculated using 3D viability analysis, applying a mask to include the Eci1 hexamer and a single Pex5. The Pex5 region (top part) contains strong contrast, indicating significant conformational variability. (II-XI) Image data set was separated based on the above three eigenvectors into 10 clusters, and each cluster was subjected to 3D refinement. Shown are slabs through the refined maps, which were low-pass filtered to 10 Å. Eci1 coordinates were docked in for reference (red). Pex5 and the interface are best resolved in the map in (II), where a continuous density appears between Eci1 and Pex5. This map was used for downstream processing. The interface and Pex5 are less well resolved in the other maps (III-XI), although Pex5 remains anchored to Eci1 at two positions, one of them formed by the PTS1 signal peptide (VI-XI). D) Single particle cryo-EM processing workflow. This graphical representation outlines the process as detailed in the methods section. E) Refined cryo-EM maps used for model building. Plots for the refinement map and local-refinement map are shown in panels I-III and IV-VI, respectively. (I, IV) 3D maps colored according to local resolution estimates. (II, V) Fourier shell correlation (FSC) curves. (III, VI) Angular distribution plots.

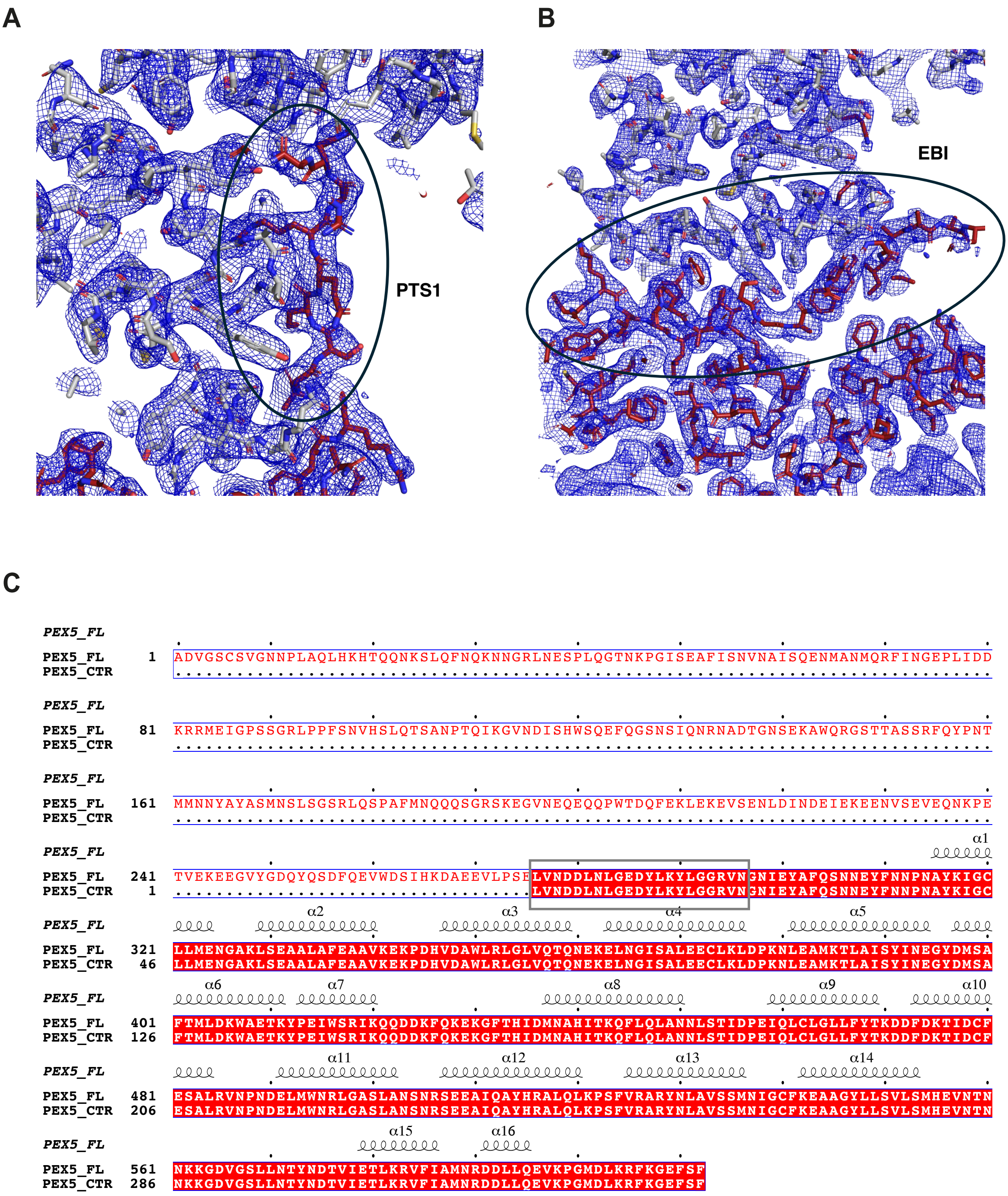

Fig. S4. The resolved Pex5-Eci1 complex structure reveals previously unseen residues of Pex5. Electron density of PTS1 and EBI is depicted in A) and B). Well-defined electron density map, showing the residues involved in the EBI and PTS1 binding interfaces. Amino acid residues from Pex5 are depicted in gray, while those from Eci1 are shown in red. C) The electron density-containing C-terminal domain of Pex5 (Pex5_CTR) was aligned with its full-length sequence (Pex5_FL), including the previously unobserved structure corresponding to the segment (grey box).

Journal of Cell Science • Supplementary information

A

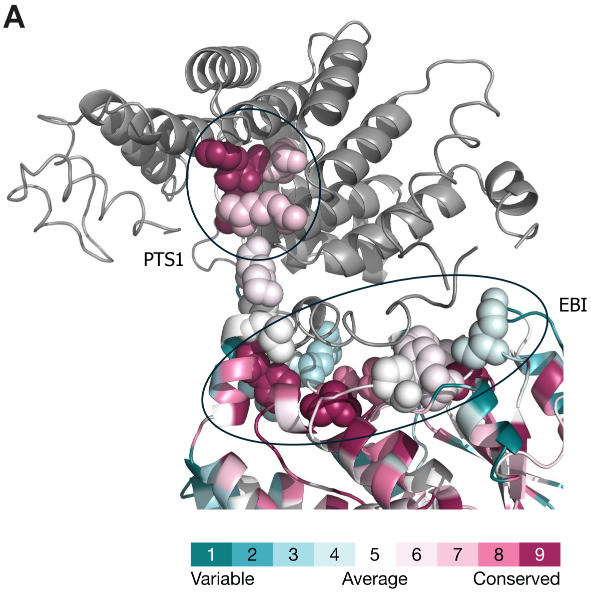

B

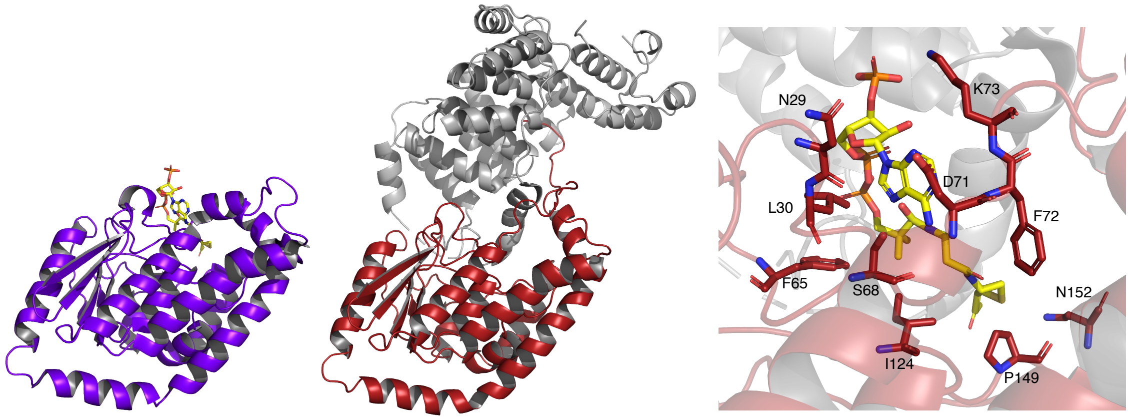

Journal of Cell Science • Supplementary information

C

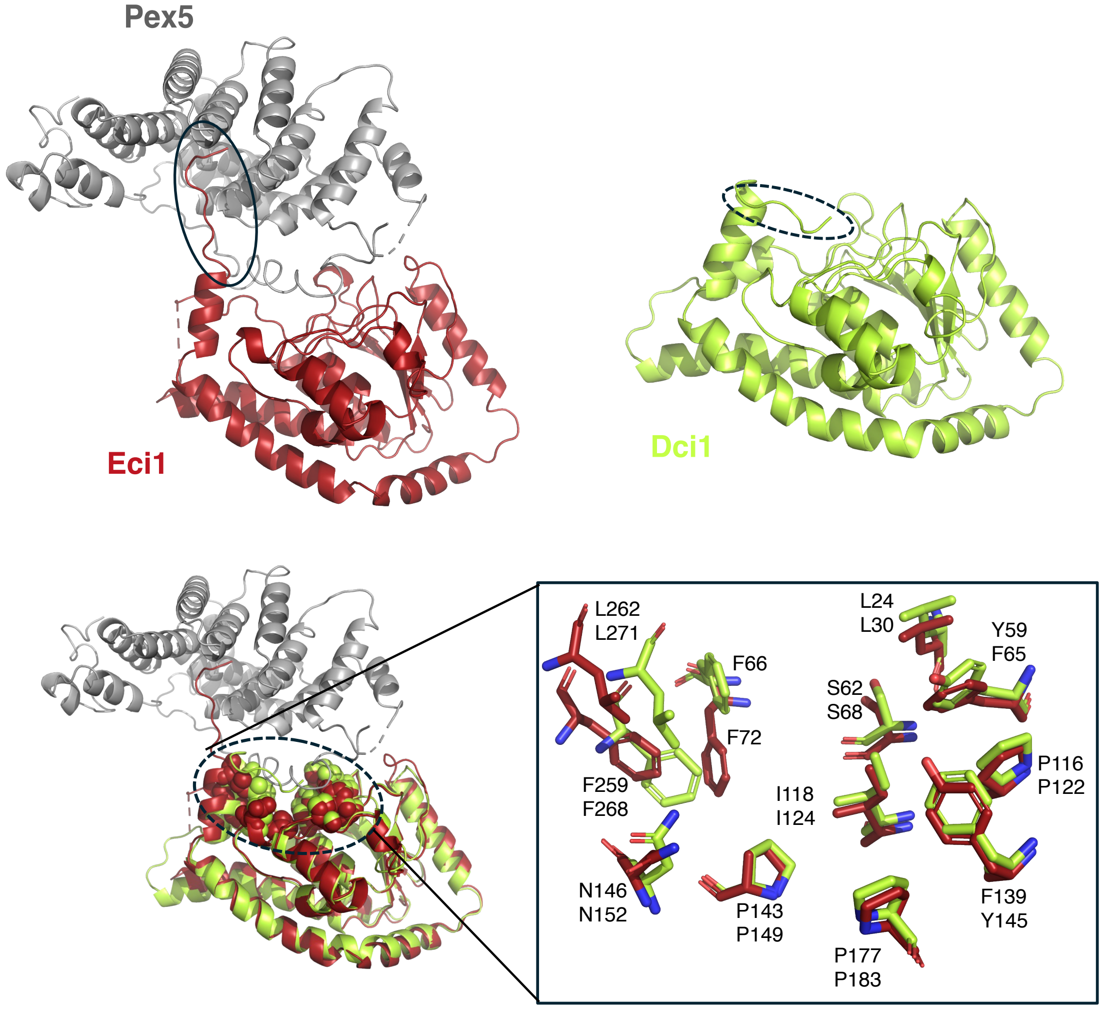
Pex5
Eci1
Dci1
L262
L271
F66
F72
F259
F268
N146
N152
P143
P149
L24
L30
Y59
F65
S62
S68
I118
I124
P116
P122
F139
Y145
P177
P183

Fig. S5. The residues of Eci1 involved in binding the EBI are conserved and functionaly versatile. A) Evolutionary conservation of the Cryo-EM resolved Eci1 structure is illustrated using the Consurf server (Ashkenazy et al., 2016), which estimates and visualizes evolutionary conservation in homologous proteins. Highly conserved amino acids are depicted in maroon, while the least conserved ones are shown in cyan. The evolutionary conservation of amino acids in Pex5 and related proteins shows that the C-terminal domain (CTD) of Pex5 is highly conserved, whereas the N-terminal domain (NTD) shows greater variability. Notably, the EBI and the anchoring point at the complex interface (residues ^{419}IKQDDKFQKEK^{430}) are among the least conserved regions within the otherwise conserved CTD, and these regions are indicated by black boxes. Notably, residues in Eci1 that interact with Pex5 in both the PTS1 and the EBI binding interfaces are highly conserved. These interacting residues are represented as spheres. B) Eci1 residues interacting with Pex5 are also shared for substrate binding. The structure of Eci1 (purple) bound to acetoacetyl-CoA (CAA) (yellow) (PDB entry 4ZDB) revealed that CAA interacts with Eci1 through hydrogen bonds and hydrophobic interactions. The cryo-EM structure of Eci1-Pex5 (middle) shows that the NTD segment of Pex5 occupies the same position as CAA. The binding of Eci1 to both CAA and the NTD segment of Pex5 involves many common residues (right), including N29, L30, F65, S68, D71, F72, K73, I124, P149, and N152. CAA is represented as stick figures with carbon atoms colored yellow, nitrogen atoms blue, oxygen atoms red, and phosphate atoms orange. The Eci1 residues interacting with CAA and the NTD of Pex5 are represented as sticks with carbon atoms colored red, nitrogen atoms blue, and oxygen atoms orange. C) The Dci1 model aligns with Eci1 in residues involved in interacting with Pex5 via the novel EBI. The distinct segment at the Eci1 (red) C-terminal end, including the PTS1 tripeptide, is pointed toward the TPR domain of Pex5 (highlighted in a black circle). In contrast, the equivalent segment of the predicted Dci1 model (green) in the absence of Pex5 points in a different direction (highlighted in the black dashed circle). Many residues of Eci1 within the novel EBI that interact with Pex5 are highly conserved in its paralog Dci1. The residue numbering refers to Dci1 at the top and Eci1 at the bottom.

Journal of Cell Science • Supplementary information

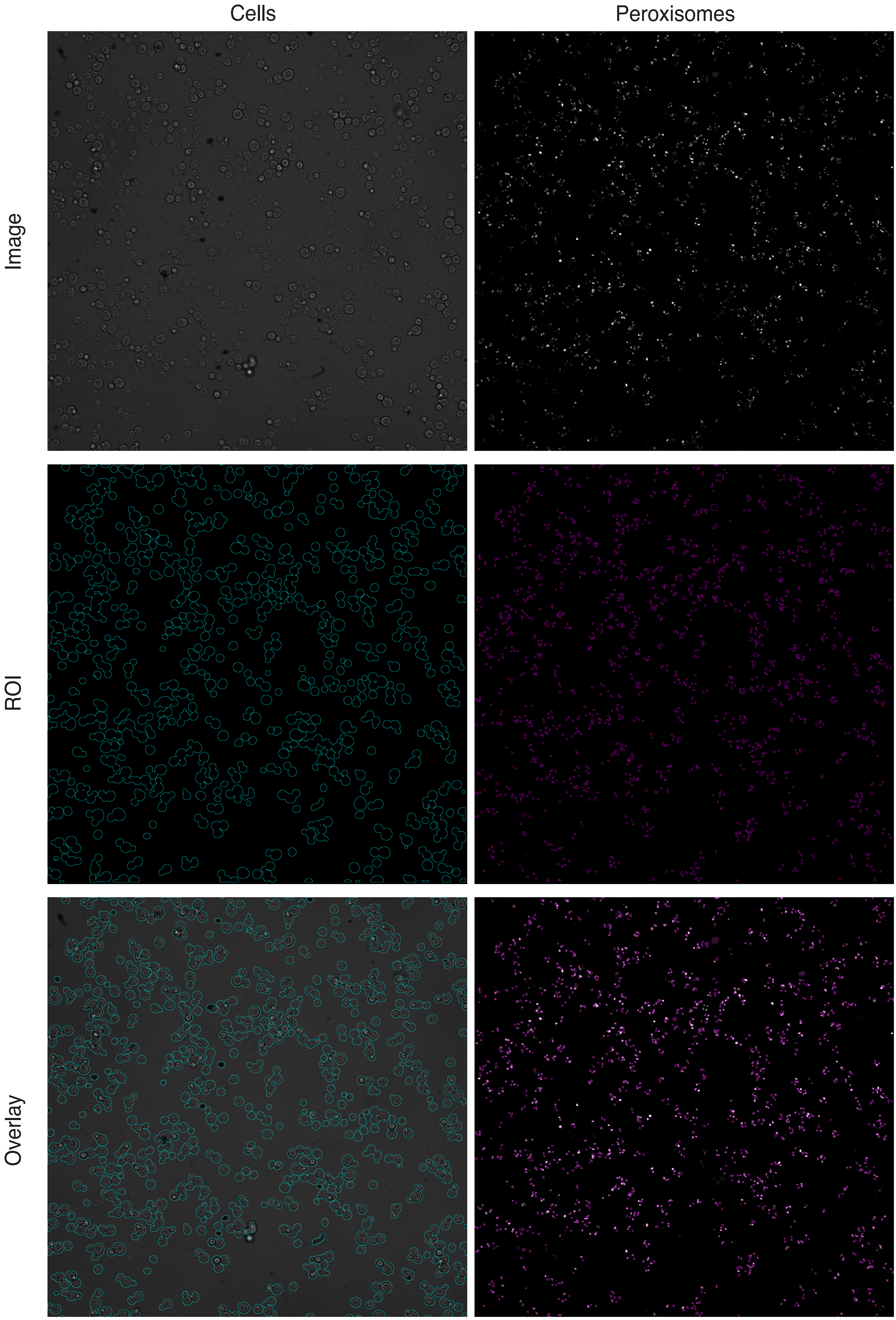

Fig. S6. Yeast cells and peroxisomes are successfully segmented using an in-house ImageJ script. Shown in this figure are cells from the data in Figure 1C – Eci1 WT. The ROIs depict the segmentation of the yeast cells (cyan) and of peroxisomes (magenta).

Table S1. S. cerevisiae and E. coli strains used in this study. A table summarizing all strains of S. cerevisiae and E. coli used in the experiments of this study.

Available for download at
https://journals.biologists.com/jcs/article-lookup/doi/10.1242/jcs.263890#supplementary-data

Table S2. Primers used in this study. A table summarizing all primers used in the experiments of this study. All primers up to 9824 (including) were designed using the online tool "Primers-4-Yeast" (Yofe and Schuldiner, 2014).

Available for download at
https://journals.biologists.com/jcs/article-lookup/doi/10.1242/jcs.263890#supplementary-data

Table S3. Plasmids used in this study. A table summarizing all plasmids used in the experiments of this study. Plasmids based on the backbones pFA6, pYM, pKL, and SWAT were used in S. cerevisiae; Plasmids based on the backbones pET and pACYDuet were used for expression in E. coli.

Available for download at
https://journals.biologists.com/jcs/article-lookup/doi/10.1242/jcs.263890#supplementary-data

Table S4. Cryo-EM data collection and refinement statistics of Pex5-Eci1. This table summarized the Cryo-EM data for determining the structure of the Pex5-Eci1 complex.

Available for download at
https://journals.biologists.com/jcs/article-lookup/doi/10.1242/jcs.263890#supplementary-data

Journal of Cell Science • Supplementary information
