# Supplementary material for: A cryo-electron microscopy structure of yeast Pex5 in complex with a cargo uncovers a novel binding interface
Source: J Cell Sci. 2025 Jun 26;138(12):jcs263890. doi: 10.1242/jcs.263890 (PMC12273641; doi:10.1242/jcs.263890)
Supplement: Supplementary file 1 [file joces-138-263890_review_history.pdf]

A cryo-electron microscopy structure of yeast Pex5 in complex with a cargo uncovers a novel binding interface

Lior Peer, Orly Dym, Nadav Elad, Asa Tirosh, Jossef Jacobovitch, Ehud Sivan, Mor Angel, Shira Albeck, Maya Schuldiner, Yoav Peleg and Einat Zalckvar
DOI: 10.1242/jcs.263890

Editor: Pedro Carvalho

Review timeline

Submission to Review Commons:	4 June 2024
Submission to Journal of Cell Science:	31 January 2025
Editorial decision:	12 February 2025
First revision received:	5 April 2025
Accepted:	13 April 2025

Reviewer 1

Evidence, reproducibility and clarity

Summary:

Proteins are imported into peroxisomes by mobile receptors such as PEX5. PEX5 recognizes cargo proteins in the cytosol by their peroxisome targeting signal (PTS) and then shuttles them across the peroxisomal membrane into the matrix. While most peroxisomal proteins contain well-characterized signals that bind to PEX5 either directly (PTS1) or through PEX7 (PTS2), some proteins interact with PEX5 independently of these canonical signals. The molecular basis of these unconventional interactions has been poorly understood.

The manuscript by Peer et al. deals with one such protein called Eci1 in yeast. Eci1 has a PTS1 signal at its C terminus and a putative PTS2 signal at its N terminus, yet the authors show that neither of these signals is required for import of Eci1 into peroxisomes. They also show that import of Eci1 cannot be entirely explained by piggy-backing on its paralog Dci1. Regardless, import of Eci1 depends entirely on PEX5, indicating that Eci1 can bind to PEX5 unconventionally. To identify this additional interface, the authors solve the cryo-EM structure of PEX5 bound to Eci1 (which is a hexamer). Surprisingly, the structure reveals that PEX5 binds to only one of the six Eci1 subunits, and that two distinct interfaces are apparent. One reflects the canonical interaction between the PTS1 signal of Eci1 and the receptor's cognate PTS1-binding TPR domain. The other interface is novel and of potential interest. It involves a region of Eci1 that engages a segment of PEX5 upstream of the TPR domain. This segment has not been previously implicated in binding protein cargo.

Major issues:

1. The major issue with the paper is that the novel interface between Eci1 and PEX5 has not been demonstrated to be important for import into peroxisomes. Specifically, mutagenesis of both sides of the interface is required to demonstrate that this interaction mediates import of Eci1 lacking the canonical PTS1 signal (and also in the absence of the paralog Dci1). Such data are indisputably a precondition for publication of this paper. Pull-down experiments should also be performed to demonstrate that the interface is sufficient for interacting with PEX5 in the absence of the PTS1 signal on Eci1.
2. The paper hinges on the demonstration of a residual interaction between PEX5 and Eci1 lacking its PTS1 signal. However, the pull-down experiment in Figure 3 that allegedly shows this result

© 2025. Published by The Company of Biologists under the terms of the Creative Commons Attribution License (https://creativecommons.org/licenses/by/4.0/).

lacks a critical control for non-specific binding of Eci1 to the nickel beads alone. Also, this experiment does not show a direct interaction between PEX5 and Eci1, since the two proteins are co-expressed in bacteria and then pulled down using an engineered His-tag in PEX5. This experiment should be repeated using PEX5 and Eci1 purified separately and then mixed in vitro. Please show a coomassie-stained SDS-PAGE gel to assess protein purity in addition to the immunoblot, and please show the pull-down in a more conventional way comparing the input and the bound fraction (it is unclear what is meant by soluble and elution fractions).

3. The presentation of the structure in Figure 4 should be improved. An overview of the complex should be shown first, and then each interface should be pointed out in a different view (and accordingly labeled). It is distracting and not necessary to show all six subunits of Eci1 in different colors. The non-conventional interface should be shown more clearly, with key amino acids numbered and labeled, and the configurations of their side chains highlighted. Please also highlight the salt bridges and hydrogen bonds at this interface that are mentioned in the text but never illustrated.
4. The data in Figs. S2 and S3 raise doubts about the reported resolution of PEX5 in the cryo-EM structure. Please provide examples of the density map and the fit to the model.
5. Please provide data for the purification of the complex between PEX5 and Eci1, including a gel-filtration chromatogram and an SDS-PAGE gel of the purified sample used for cryo-EM.
6. OPTIONAL: The observation that the non-conventional interface between PEX5 with Eci1 corresponds to the site of CoA binding is interesting. This interaction might keep the enzyme inactive while in the cytosol and bound to PEX5, until it would be correctly delivered into peroxisomes and released from the receptor. Alternatively, it could also reflect regulation of Eci1 import by CoA. This idea could easily be tested by pull-down experiments performed with or without CoA, or perhaps by an in vitro Eci1 activity assay in the presence or absence of PEX5. The significance of the paper would be considerably improved if this interaction reflected a mechanism to regulate Eci1 activity or import.

Minor issues:

1. The manuscript has many grammatical mistakes which should be addressed. The absence of line numbers precludes us from indicating specific issues.
2. In general, when referring to a single subunit from the Eci1 hexamer, please use the terms subunit or protomer, and avoid the use of the term monomer which is misleading.
3. In Fig. 1C, it is unclear whether the experiment was performed in the absence or presence of PEX11. Since the paper hinges on the demonstration of an unconventional interaction between Eci1 and PEX5, perhaps this experiment should be performed in pex11 knockout cells (to enlarge peroxisomes as in Fig. 1B) to show that the residual peroxisomal localization indeed corresponds to the matrix.
4. In Fig. 6, it would help to show each structure individually and then the overlay.
5. Fig. S4 should include a scale bar and box size.
6. Why are phosphorylation sites indicated in Fig. S6?
7. In Fig. S8, please show the structures of Eci1 bound to PEX5 and to CoA individually, and then the overlay. The figure is very diffucult to understand otherwise.
8. In Fig. S9, please label the homologous interface residues on Eci1 and Dci1 in individual views, and then show the overlay.

Significance

The main finding of the paper is a noncanonical interaction between Eci1 and the peroxisomal import receptor PEX5. This interaction could solve a longstanding mystery about how Eci1 can be targeted to peroxisomes in the absence of its canonical peroxisome targeting signal. Because the authors have not demonstrated that this interaction is sufficient for import of Eci1 in vivo, this key conclusion of the paper remains unconfirmed. If this omission were corrected, the paper would add another example to the growing list of proteins that are imported into peroxisomes by binding unconventionally to PEX5.

The authors employ an interesting strategy to confirm that Eci1 is correctly imported into the peroxisomal matrix in vivo (and not just recruited to the cytosolic surface of the peroxisomal membrane). This strategy involves enlarging peroxisomes (which normally are diffraction limited) by knocking out a factor required for peroxisome division, allowing the matrix to be resolved

© 2025. Published by The Company of Biologists under the terms of the Creative Commons Attribution License (https://creativecommons.org/licenses/by/4.0/).

from the limiting membrane by light microscopy. Failure to adequately demonstrate import into the matrix had plagued many earlier studies on protein targeting to peroxisomes. The strategy employed in this paper could therefore be useful to other researchers.

In its current form, the manuscript would be of some interest to the peroxisomal community and perhaps also to researchers studying protein targeting to membrane-bounded organelles. However, if the authors could show that the novel interface between PEX5 and Eci1 functions in part to regulate Eci1 enzymatic activity (or conversely, Eci1 import by CoA), then the paper would be of much broader interest to the fields of metabolism and metabolic regulation.

Reviewer 2

Evidence, reproducibility and clarity

Peroxisomes are single membrane organelles conserved in all eukaryotes and play important roles in various metabolic reactions, such as beta oxidation of fatty acids. In general, proteins localized in the peroxisomal matrix encode either a C-terminal PTS1 signal or an N-terminal PTS2 signal, and Pex5 acts as a cargo receptor in the PTS1 pathway and Pex7 in the PTS2 pathway, respectively. Previous studies have suggested that some matrix proteins (e.g., Eci1) are transported into the peroxisomal matrix in the PTS1-independent manner, but the mechanism is still unclear. In the present study, Peer et al. determined the Cryo-EM structure of the Pex5-Eci1 complex, which revealed a new interaction site that is distinct from the recognition site of the canonical PTS1 signal, providing important insight into the PTS1-independent, but the Pex5-dependent matrix protein transport. This study by Peer et al. will be of interest to a broad readership in basic cell biology other than peroxisomes. The reviewer feels that the manuscript needs to be revised in the following points.

Major comments

1. The authors showed that Pex5 binds to Eci1 in a PTS1 signal-independent manner from pull-down experiments in Figure 2, but this result is qualitative. If the authors add quantitative data on the interaction between Pex5 and Eci1 from isothermal titration calorimetry (ITC) or surface plasmon resonance (SPR), this would make this paper more convincing. This could be done in 2 months.
2. It is not clear to what extent the new interaction sites between Pex5 and Eci1 is important for transport to peroxisomes, as revealed in this study. I suggest, for example, expressing Eci1 with a mutation at a site involved in interaction with Pex5 in yeast and analyzing its effect on peroxisomal localization as additional experiments anew. I believes that this could be done in about 2 months.

Minor comments

1. The results of yeast cell imaging in Figures 1, 2 and S1 are all qualitative and not quantitative. Furthermore, there are no descriptions of the experimental reproducibility of the data. I suggest that these points need to be improved.
2. I feel that information of sample preparation for cryo-EM analysis of the Pex5-Eci1 complex is not enough since it is only described in the methods. I suggest the authors to add the results of gel-filtration chromatography and CBB-stained SDS-PAGE in the manuscript.
3. The authors discuss the interaction interface between Pex5 and Eci1 in Figures 4 and 5, but the manuscript presented at this stage is difficult at least for me to understand the interaction between them. I recommend the authors to add new figure(s) to show more detailed interaction. Also, I suggest that cryo-EM density map around the interaction region between Pex5 and Eci1 should be presented more detail.

Significance

My expertise is in yeast cell biology and structural biology. From this perspective, I think that the strengths of this study are, first, that Pex5-dependent peroxisomal transport of Eci1 in yeast cells occurs independently of PTS1 signal and its paralog Dci1, and that the cryo-EM structure of the

© 2025. Published by The Company of Biologists under the terms of the Creative Commons Attribution License (https://creativecommons.org/licenses/by/4.0/).

Pex5-Eci1 complex reveals a new interaction site other than PTS1 between Pex5 and Eci1. This work is of broad interest not only to peroxisomes, but also to many cell biologists specializing in organelles, and ultimately to structural biologists. On the other hand, the authors' cryo-EM data suggest that 2-3 molecules of Pex5 bind to the Eci1 hexamer. However, it is unclear how the binding of multiple Pex5 molecules to the Eci1 hexamer affects their transport to peroxisomes, and further analysis is needed to elucidate the transport mechanism in more detail.

Reviewer 3

Evidence, reproducibility and clarity

Summary:

It has been known for many years that some peroxisomal proteins are imported by the major peroxisomal protein import receptor Pex5, which recognises the C terminal targeting signal PTS1, despite either lacking a PTS1 or if the PTS1 is blocked.
Some proteins are also able to 'piggyback' into peroxisomes by binding to a partner which possesses a PTS. Eci1, the subject of this study is such a protein. This manuscript identified a PTS1-independent, non-canonical interaction interface between S. cerevisiae PEX5 and imported protein Eci1. Confocal imaging was used to observe the PTS1-independent import of Eci1 into peroxisomes and to establish dependence of Pex5 even in the absence of its piggyback partner Dci1. The authors purified the Pex5-Eci1 complex and used Cryo-EM to provide a structure of the purified PEX5-Eci1 complex. In general, this manuscript is well written and easy to read.

Major points

Most of the experiments presented are well-designed and accompanied with appropriate controls. However, please mention how many times the experiments have been repeated and how many biological samples were used in the analysis.The authors should also consider the following suggestions substantiate their conclusions:

Figure 1A: Include full-length Eci1 with an N-terminal fluorophore, Eci1 PTS1-deletion with N-terminal fluorophore, and the PTS1 deletion with a C-terminal fluorophore, to control for any disturbance of targeting by the C terminal NG tag.

Figure 1C: Confirm the Eci1 and Dci1 levels (if an antibody is available for the latter) by western blot. It is difficult to compare expression levels when comparing just a small number of cells in the microscope. Western blot would give a more robust evaluation of protein levels and help corroborate the claim that Eci1 expression is decreased in the absence of Dci1 if the authors wish to stand by this conclusion.

Figure 2: confirm the deletion and overexpression of PEX9, PEX5, and PEX7 by western blot of the relevant strains. The production of these strains is not described in the manuscript. If they have been previously described this should be referenced if not it should be included.

Figure 2: Validate these strains by checking import of a canonical PTS1 and canonical PTS2 and pex9 dependent protein to ensure they function as they should, unless these strains have been published elsewhere in which case their characterisation can be referenced.

Figure 3: The gel should include a standard of a known amount of the lysate used in the pull down to enable a semi-quantitative estimation of the amount of Eci1 protein captured by PEX5 with and without its PTS1. Also include Eci1 with a C-terminal fluorophore to be comparable with the in vivo data in Figs 1 and 2. A control with no pex5 for background would be useful. A full Coomassie-blue stained gel (not western blot) is required to demonstrate the direct interaction as with the western blot it cannot be excluded that other proteins bridge the interaction since this is a pull down from lysate not purified proteins.
OPTIONAL:Interestingly the surface on Eci1 which binds pex5 is where CoA binds in the active enzyme. Would CoA compete for binding to Pex5? (could add it into the pull down expt?)

© 2025. Published by The Company of Biologists under the terms of the Creative Commons Attribution License (https://creativecommons.org/licenses/by/4.0/).

Figure S2: The complex between pex5 and eci1 is solved by cryo EM. Eci1 is hexameric usually 1 but sometimes 2 or 3 pex5s are bound to the complex. The size-exclusion chromatography figure with calculated molecular weight is required to support the stoichiometry. A native gel to show the complex, as well as a denaturing gel (using the complex) to show the individual proteins will be beneficial.

Figure S9: Would Eci1 compete with Dci1 to bind to Pex5 since they share highly conserved interfaces? If so, why did the deletion of Dci1 impair Eci1 location? Or is this just reduced expression in the dci1 deletion background? (See point 2) This seems counterintuitive/contradictory so please comment.

OPTIONAL: As the authors acknowledge this work is in vitro. It would have been interesting to examine the role of this interface in vivo by mutating one or more of the residues in Eci 1 identified as being important for the interaction. Granted that mutation can affect the folding of the protein, but the binding region is on the surface so it may not, and this can be readily checked e.g by enzyme activity or limited proteolysis.

OPTIONAL: Similarly, it would have been interesting to see if mutating the residues of PEX5 involved in the interface affect the import of other cargoes than eci1 or if reciprocal mutations in pex5 and Eci1 e.g switching charges could restore an import defect.

OPTIONAL If 8 & 9 isn't possible could a co-evolutionary analysis of the interface residues provide further independent evidence for their functional importance? They have looked at conservation of residues in Eci1 but this could be extended to a co-evolution analysis.

Minor points

Figure 1C and throughout the manuscript state clearly whether the same confocal settings are used when comparing fluorescence intensity of different images/samples.

Figure S2B: Please use different colours for PEX5 and Eci1 for clarity.

Figure 4A: please indicate the PTS1 for the other 5 molecules of Eci1. Are they buried? Or not seen? Please add explanation.

Figure 4B, C, and D: please colour the circled helix in PEX5 so that it can be more easily seen.

Please indicate the EBI-mediated interaction in Figure 4C. The relationship between 4C and 4D could be explained better as they are not viewed from the same direction

Figure S3: As the authors indicated, Pex5 binds with multiple conformations and forms a variable interface with an Eci1 subunit. Does this mean different types of non-canonical interface are possible? Please discuss this.

Figure 5A and B: they should be labelled as PEX5 TPR domain

Figure S8 is very helpful in understanding the interface and could be included in Figure 5.

Significance

While cargo recognition by Pex5-PTS1 is well understood in molecular detail there are proteins which either lack a PTS1 or have a nonessential PTS1 that still require Pex5 for import into peroxisomes. This study provides a structural view of interaction between Pex5 and its cargo Eci1, a protein that does have a PTS1 but which is not essential for import. It's not the first example of a PEX5-cargo structure to show a non-canonical binding interface and the results are compared to the human pex5-AGT structure. It is an important addition to understanding how so-called PTS context dependent or non1 non2 proteins can be imported. Is this the first structure showing Pex5 bound to an oligomer cargo? Previous work is appropriately cited in the manuscript.

© 2025. Published by The Company of Biologists under the terms of the Creative Commons Attribution License (https://creativecommons.org/licenses/by/4.0/).

The study will be of interest to audiences interested in protein-protein interaction and in protein targeting to organelles. This manuscript presents additional knowledge on how an oligomeric PTS1-independent protein can be imported into peroxisomes. The potential of other proteins using the similar importing mechanism can be tested to understand how one receptor can use apparently multiple binding modes to import a wide range of different proteins.

Author response to reviewers' comments

Manuscript number: RC-2024-02541
Corresponding authors: Einat Zalckvar, Yoav Peleg, Maya Schuldiner.

1. General Statements

This section is optional. Insert here any general statements you wish to make about the goal of the study or about the reviews.

We want to thank the reviewers for their positive feedback and meaningful input, which dramatically improved the manuscript and helped us to clarify, process, and deepen core points in the manuscript. Please find below the point-by-point response to the reviewers' comments.

We believe that our work brings forward insights regarding the complex protein targeting mediated by Pex5 and highlights the intricate and complex nature of protein targeting within eukaryotic cells.

We look forward to working with you towards the publication of this work.

2. Point-by-point description of the revisions

Reviewer #1

(1) The major issue with the paper is that the novel interface between Eci1 and PEX5 has not been demonstrated to be important for import into peroxisomes. Specifically, mutagenesis of both sides of the interface is required to demonstrate that this interaction mediates import of Eci1 lacking the canonical PTS1 signal (and also in the absence of the paralog Dci1). Such data are indisputably a precondition for publication of this paper. Pull-down experiments should also be performed to demonstrate that the interface is sufficient for interacting with PEX5 in the absence of the PTS1 signal on Eci1.

We thank the reviewer for these important points. To answer them:

1. To further verify the direct interaction between Pex5 and Eci1 lacking its PTS1, we performed Biacore analysis to assess the binding of Pex5 to either WT-Eci1 or Eci1 lacking its PTS1 (Figures 3B and Supplementary Figure S4). The results demonstrate that Pex5 can directly bind to Eci1 even in the absence of its PTS1 and independently of Dci1.

2. To further study whether the amino acids mediating the newly identified binding interface with Pex5 are sufficient for peroxisomal targeting, we fused GFP to either the last 10 amino acids of Eci1 (including the PTS1) or to residues F65-L153 of Eci1 (the newly identified binding interface). As expected, the PTS1 of Eci1 was sufficient to mediate peroxisomal targeting in a Pex5- dependent manner. However, the GFP-F65-L153 expression was extremely low, with only a weak GFP signal observed in the cytosol (see figure below).
We hypothesized that the GFP-F65-L153 fusion protein might be misfolded and degraded by the proteasome. To test this, we repeated the experiment in the presence of the proteasome inhibitor MG132. However, the GFP signal remained very weak, suggesting that proteasomal degradation is

© 2025. Published by The Company of Biologists under the terms of the Creative Commons Attribution License (https://creativecommons.org/licenses/by/4.0/).

unlikely to be the primary cause of the low expression.

In conclusion, although Eci1 can directly bind to Pex5 in the absence of its PTS1 and the structural data reveal that the two proteins interact through multiple binding interfaces, further investigation is required to determine the functional significance of the newly identified interface. It is plausible that this additional binding site plays a role in facilitating dynamic targeting under different metabolic conditions. We now elaborate on this point further in the manuscript.

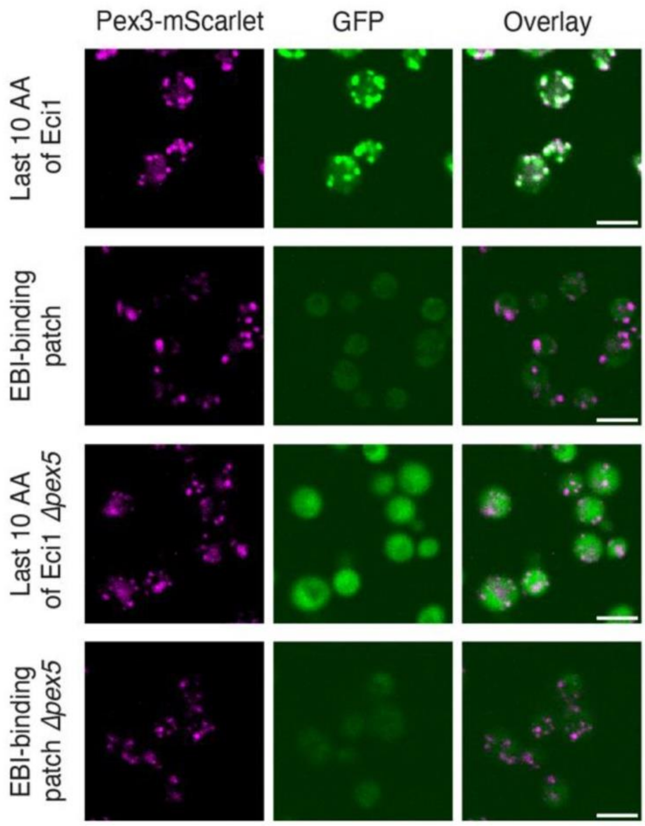

The EBI-binding patch of Eci1 is not sufficient to mediate targeting to peroxisomes. Fluorescence microscopy images depicting the N-terminal GFP fusions to either the last 10 amino acids of Eci1 (L271-L280, “Last 10 AA of Eci1”) or the residues comprising the newly identified binding interface (F65-L153, “EBI-biding patch”). As expected, GFP-Eci1 PTS1 co-localizes to peroxisomes in a Pex5-dependent manner. However, the newly identified Eci1-binding patch is weakly expressed in the cytosol.

(2) The paper hinges on the demonstration of a residual interaction between PEX5 and Eci1 lacking its PTS1 signal. However, the pull-down experiment in Figure 3 that allegedly shows this result lacks a critical control for non-specific binding of Eci1 to the nickel beads alone. Also, this experiment does not show a direct interaction between PEX5 and Eci1, since the two proteins are co-expressed in bacteria and then pulled down using an engineered His-tag in PEX5. This experiment should be repeated using PEX5 and Eci1 purified separately and then mixed in vitro.

Please show a coomassie-stained SDS-PAGE gel to assess protein purity in addition to the immunoblot, and please show the pull-down in a more conventional way comparing the input and the bound fraction (it is unclear what is meant by soluble and elution fractions).

To address the above concerns, we expressed Pex5 and Eci1 (WT or ΔPTS1) separately. We then mixed Pex5 and Eci1 and performed Biacore analysis to measure their direct interactions (see Figure 3B and Supplementary Figures S4 and S5). The results demonstrate that Eci1 lacking its PTS1 can bind directly to Pex5, however with a weaker interaction compared to full- length Eci1.

© 2025. Published by The Company of Biologists under the terms of the Creative Commons Attribution License (https://creativecommons.org/licenses/by/4.0/).

(3) The presentation of the structure in Figure 4 should be improved. An overview of the complex should be shown first, and then each interface should be pointed out in a different view (and accordingly labeled). It is distracting and not necessary to show all six subunits of Eci1 in different colors. The non-conventional interface should be shown more clearly, with key amino acids numbered and labeled, and the configurations of their side chains highlighted. Please also highlight the salt bridges and hydrogen bonds at this interface that are mentioned in the text but never illustrated.

Done.

(4) The data in Figs. S2 and S3 raise doubts about the reported resolution of PEX5 in the cryo-EM structure. Please provide examples of the density map and the fit to the model.

Done. We now show density maps in Supplementary Figure S11.

(5) Please provide data for the purification of the complex between PEX5 and Eci1, including a gel-filtration chromatogram and an SDS-PAGE gel of the purified sample used for cryo-EM.

Done. We now added the data in Supplementary Figure S6.

(6) OPTIONAL: The observation that the non-conventional interface between PEX5 with Eci1 corresponds to the site of CoA binding is interesting. This interaction might keep the enzyme inactive while in the cytosol and bound to PEX5, until it would be correctly delivered into peroxisomes and released from the receptor. Alternatively, it could also reflect regulation of Eci1 import by CoA. This idea could easily be tested by pull-down experiments performed with or without CoA, or perhaps by an in vitro Eci1 activity assay in the presence or absence of PEX5. The significance of the paper would be considerably improved if this interaction reflected a mechanism to regulate Eci1 activity or import.

We thank the reviewer for this insightful comment. We find the observation that Pex5 and CoA bind to the same surface extremely interesting. In most of the structures that we identified, only one subunit of the Eci1 hexamer was bound to Pex5 while the remaining five subunits were unbound and potentially available to bind CoA. Under this scenario, Pex5 binding will not completely abolish the activation of Eci1. Additionally, we now discuss an alternative hypothesis suggesting that this mode of interaction facilitates the targeting of CoA to peroxisomes.

Minor issues:

(1) The manuscript has many grammatical mistakes which should be addressed. The absence of line numbers precludes us from indicating specific issues.

We addressed the mistakes.

(2) In general, when referring to a single subunit from the Eci1 hexamer, please use the terms subunit or protomer, and avoid the use of the term monomer which is misleading.

Thank you. We now use the term subunit.

(3) In Fig. 1C, it is unclear whether the experiment was performed in the absence or presence of PEX11. Since the paper hinges on the demonstration of an unconventional interaction between Eci1 and PEX5, perhaps this experiment should be performed in pex11 knockout cells (to enlarge peroxisomes as in Fig. 1B) to show that the residual peroxisomal localization indeed corresponds to the matrix.

The original experiment in which we deleted DCI1 was performed without enlarging peroxisomes. Encouraged by the reviewer's comment, we now repeated the experiment under conditions that promote peroxisome enlargement (see the figure below). Our results confirm that Eci1-mNG is targeted to peroxisomes in the absence of Dci1. Interestingly, we observed that in the absence of Dci1, Eci1-mNG is localized to the peroxisomal membrane and seems to be enriched in specific domains. Using our microscopy setup, we cannot determine whether Eci1- mNG is attached to the

© 2025. Published by The Company of Biologists under the terms of the Creative Commons Attribution License (https://creativecommons.org/licenses/by/4.0/).

membrane from the cytosolic side, embedded within the membrane, or localized in the peroxisomal matrix while interacting with the membrane. Future study beyond the scope of this work is required to determine this point.

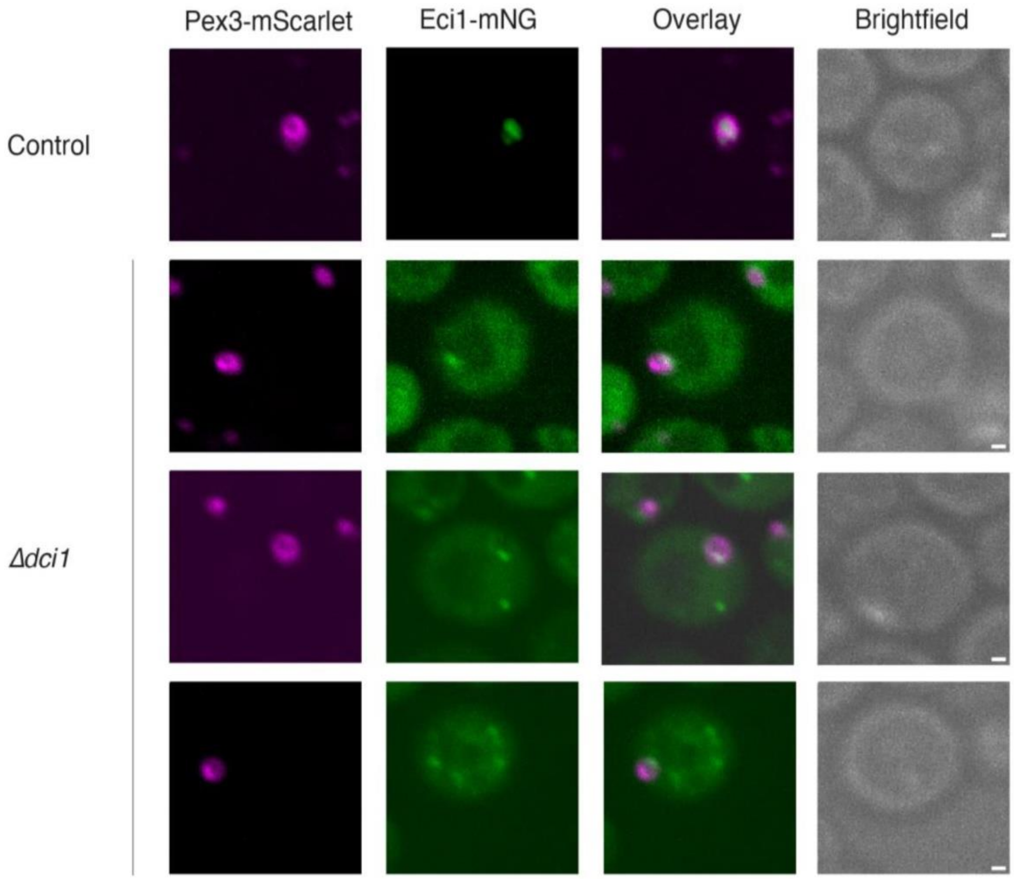

Eci1-mNG co-localizes to peroxisomes in the absence of Dci1. High-resolution (SORA) fluorescence microscopy images examining the sub-peroxisomal localization of Eci1-mNG on the background of *Δpex11 Δdci1*. Scale: 500 nm

(4) In Fig. 6, it would help to show each structure individually and then the overlay.

Done.

(5) Fig. S4 should include a scale bar and box size.

Fixed (now Supplementary Figure S9).

(6) Why are phosphorylation sites indicated in Fig. S6?

We agree with the reviewer that there is no added value for these indications. We now removed the phosphorylation sites (now Supplementary Figure S12).

(7) In Fig. S8, please show the structures of Eci1 bound to PEX5 and to CoA individually, and then the overlay. The figure is very difficult to understand otherwise.

Done (now Supplementary Figure S14).

(8) In Fig. S9, please label the homologous interface residues on Eci1 and Dci1 in individual views, and then show the overlay.

Fixed (now Supplementary Figure S15).

© 2025. Published by The Company of Biologists under the terms of the Creative Commons Attribution License (https://creativecommons.org/licenses/by/4.0/).

Reviewer #2

1. The authors showed that Pex5 binds to Eci1 in a PTS1 signal-independent manner from pull-down experiments in Figure 2, but this result is qualitative. If the authors add quantitative data on the interaction between Pex5 and Eci1 from isothermal titration calorimetry (ITC) or surface plasmon resonance (SPR), this would make this paper more convincing. This could be done in 2 months.

We thank the reviewer for encouraging us to perform more experiments that will strengthen this point. We now used Biacore analysis to examine and measure the interaction between Pex5 and WT Eci1, and Pex5-Eci1□*PTS1 (Figure 3 and supplementary Figures S4 and S5).*

2. It is not clear to what extent the new interaction sites between Pex5 and Eci1 is important for transport to peroxisomes, as revealed in this study. I suggest, for example, expressing Eci1 with a mutation at a site involved in interaction with Pex5 in yeast and analyzing its effect on peroxisomal localization as additional experiments anew. I believe that this could be done in about 2 months.

To further study whether the amino acids mediating the newly identified binding interface with Pex5 are sufficient for peroxisomal targeting, we fused GFP to either the last 10 amino acids of Eci1 (including the PTS1) or to residues F65-L153 of Eci1 (the newly identified binding interface). As expected, the PTS1 of Eci1 was sufficient to mediate targeting to peroxisomes in a Pex5-dependent manner. However, the GFP-F65-L153 expression was extremely low, with only a weak GFP signal observed in the cytosol (See the figure in comment 1 to reviewer 1). We thought that the GFP-F65-L153 fusion protein might be misfolded and degraded by the proteasome. Hence, we repeated the experiment using the proteasome inhibitor MG132.
Unfortunately, the signal was still very weak, suggesting that proteasomal degradation is unlikely to be the primary cause of the low expression.
In conclusion, although Eci1 can directly bind to Pex5 in the absence of its PTS1, further work is required to identify the functional role of the additional binding interface we identified. It is plausible that this newly identified binding interface plays a role in dynamic targeting under different metabolic conditions. We now further discuss this point in the manuscript.

Minor comments

1. The results of yeast cell imaging in Figures 1, 2 and S1 are all qualitative and not quantitative. Furthermore, there are no descriptions of the experimental reproducibility of the data. I suggest that these points need to be improved.

We thank the reviewer for this important comment. We performed quantifications for the Eci1- mNG signal (Figures 1, 2, and Supplementary Figure S2).
In each figure, one experiment with three technical repeats is presented; each technical contains at least 100 cells.
We added all the information to the manuscript.

2. I feel that information of sample preparation for cryo-EM analysis of the Pex5-Eci1 complex is not enough since it is only described in the methods. I suggest the authors to add the results of gel-filtration chromatography and CBB-stained SDS-PAGE in the manuscript.

Done (Supplementary Figure S6).

3. The authors discuss the interaction interface between Pex5 and Eci1 in Figures 4 and 5, but the manuscript presented at this stage is difficult at least for me to understand the interaction between them. I recommend the authors to add new figure(s) to show more detailed interaction. Also, I suggest that cryo-EM density map around the interaction region between Pex5 and Eci1 should be presented more detail.

Figure 4 was modified and now presents more details of the Pex5-Eci1 interaction. In addition,

© 2025. Published by The Company of Biologists under the terms of the Creative Commons Attribution License (https://creativecommons.org/licenses/by/4.0/).

we added a Cryo-EM density map (Supplementary Figure S11).

Reviewer #3

Most of the experiments presented are well-designed and accompanied with appropriate controls. However, please mention how many times the experiments have been repeated and how many biological samples were used in the analysis.

We thank the reviewer for this important comment.

In each figure, one experiment with three technical repeats is presented; each technical repeat contains at least 100 cells.

We now added all the information to the manuscript.

The authors should also consider the following suggestions substantiate their conclusions:

Figure 1A: Include full-length Eci1 with an N-terminal fluorophore, Eci1 PTS1-deletion with N-terminal fluorophore, and the PTS1 deletion with a C-terminal fluorophore, to control for any disturbance of targeting by the C terminal NG tag.

We examined the cellular localization of Eci1 in strains in which Eci1 was either N' or C' tagged with a fluorophore with or without its PTS1 (see figure below). Eci1 co-localized to peroxisomes when it was either tagged on its N' and expressed under an NOP1 promoter or when it was tagged on its C' while expressed under its native promoter. Interestingly, Eci1 was still localized to peroxisomes when its PTS1 was deleted. Notably, the signal of NOP1pr-GFP-Eci1ΔPTS1 was much weaker than the NOP1pr-GFP-Eci1. Nevertheless, the weak signal co-localized to peroxisomes.

Interestingly, when we masked the PTS1 of Eci1 by a fluorophore, we could observe colocalization with peroxisomes even in the absence of Dci1. Nevertheless, we could not detect co-localization with peroxisomes when we deleted the PTS1 in the absence of Dci1.

We decided not to include these results in the manuscript because our microscopy-based setup cannot detect co-localization with peroxisomes when the signal is weak or when it is spread in the cytosol. Hence, it could be that there is a low peroxisomal localization of Eci1 that we cannot detect and hence we cannot interpret the Dci1-dependency without further biochemical work.

© 2025. Published by The Company of Biologists under the terms of the Creative Commons Attribution License (https://creativecommons.org/licenses/by/4.0/).

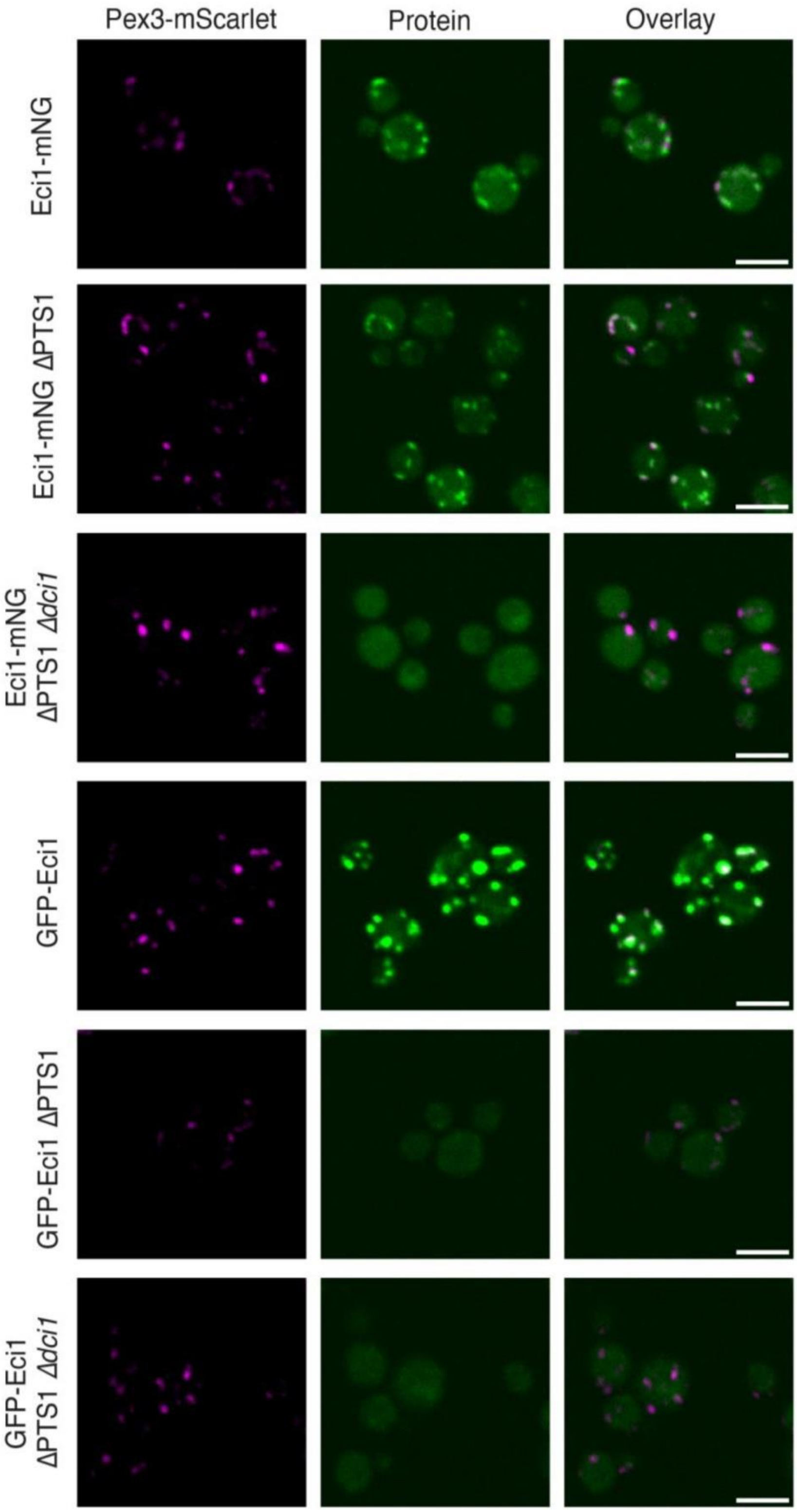

Eci1 co-localizes to peroxisomes when N' or C' tagged with mNG.

Figure 1C: Confirm the Eci1 and Dci1 levels (if an antibody is available for the latter) by western blot. It is difficult to compare expression levels when comparing just a small number of cells in the microscope. Western blot would give a more robust evaluation of protein levels and help corroborate the claim that Eci1 expression is decreased in the absence of Dci1 if the authors wish to stand by this conclusion.

© 2025. Published by The Company of Biologists under the terms of the Creative Commons Attribution License (https://creativecommons.org/licenses/by/4.0/).

We apologize for not clearly discussing the results shown in Figure 1C. Our main point is that Eci1-mNG remains co-localized to peroxisomes even in the absence of Dci1. We see that the mNG signal is weaker in the absence of Dci1, and we now quantified this signal from many cells (Supplementary Figure S2A). However, our main focus was not on expression level but rather on the ability of Eci1-mNG to localize to peroxisomes when its PTS1 is masked by mNG and in the absence of Dci1 (which we quantified as well in Figure 1C). We have now modified the text to clarify this point.

Figure 2: confirm the deletion and overexpression of PEX9, PEX5, and PEX7 by western blot of the relevant strains. The production of these strains is not described in the manuscript. If they have been previously described this should be referenced if not it should be included.

We have now added a more detailed explanation regarding creating the KO and overexpression strains (please see “Materials and methods”).

Figure 2: Validate these strains by checking import of a canonical PTS1 and canonical PTS2 and pex9 dependent protein to ensure they function as they should, unless these strains have been published elsewhere in which case their characterisation can be referenced.

Done. See Supplementary Figure S3.

Figure 3: The gel should include a standard of a known amount of the lysate used in the pull down to enable a semi-quantitative estimation of the amount of Eci1 protein captured by PEX5 with and without its PTS1. Also include Eci1 with a C-terminal fluorophore to be comparable with the in vivo data in Figs 1 and 2. A control with no pex5 for background would be useful. A full Coomassie-blue stained gel (not western blot) is required to demonstrate the direct interaction as with the western blot it cannot be excluded that other proteins bridge the interaction since this is a pull down from lysate not purified proteins. OPTIONAL:Interestingly the surface on Eci1 which binds pex5 is where CoA binds in the active enzyme. Would CoA compete for binding to Pex5? (could add it into the pull down expt?)

We thank the reviewer for the insightful comments.

We now used Biacore analysis to examine and measure the interaction between Pex5 and WT Eci1, and Pex5-Eci1□*PTS1 (Figure 3B and Supplementary Figures S4 and S5).*

We find the observation that Pex5 and CoA bind to the same surface extremely interesting. In most of the structures that we identified, only one Eci1 subunit was bound to Pex5, while the other five subunits were unbound to Pex5 and potentially available to bind CoA. We expect that under this scenario, Pex5 binding will not completely abolish the activation of Eci1. Additionally, we have now discussed another possibility that this mode of interaction facilitates the targeting of CoA to peroxisomes.

Figure S2: The complex between pex5 and eci1 is solved by cryo EM. Eci1 is hexameric usually 1 but sometimes 2 or 3 pex5s are bound to the complex. The size-exclusion chromatography figure with calculated molecular weight is required to support the stoichiometry. A native gel to show the complex, as well as a denaturing gel (using the complex) to show the individual proteins will be beneficial.

Done. Please see Supplementary Figure S6.

Figure S9: Would Eci1 compete with Dci1 to bind to Pex5 since they share highly conserved interfaces? If so, why did the deletion of Dci1 impair Eci1 location? Or is this just reduced expression in the dci1 deletion background? (See point 2) This seems counterintuitive/contradictory so please comment.

We apologize for not being clear. We show, in line with previous works, that in the absence of Dci1, Eci1-mNG is still localized to peroxisomes although it seems like its expression is lower.

OPTIONAL: As the authors acknowledge this work is in vitro. It would have been interesting to

© 2025. Published by The Company of Biologists under the terms of the Creative Commons Attribution License (https://creativecommons.org/licenses/by/4.0/).

examine the role of this interface in vivo by mutating one or more of the residues in Eci 1 identified as being important for the interaction. Granted that mutation can affect the folding of the protein, but the binding region is on the surface so it may not, and this can be readily checked e.g by enzyme activity or limited proteolysis.

To further study whether the amino acids mediating the newly identified binding interface with Pex5 are sufficient for peroxisomal targeting, we fused GFP to either the last 10 amino acids of Eci1 (including the PTS1) or to residues F65-L153 of Eci1 (the newly identified binding interface). As expected, the PTS1 of Eci1 was sufficient to mediate targeting to peroxisomes in a Pex5-dependent manner. However, the GFP-F65-L153 expression was extremely low, with only a weak GFP signal observed in the cytosol (See the figure in comment 1 to reviewer 1). We thought that the GFP-F65-L153 fusion protein might be misfolded and degraded by the proteasome. Hence, we repeated the experiment using the proteasome inhibitor MG132.

Unfortunately, the signal was still very weak, suggesting that proteasomal degradation is unlikely to be the primary cause of the low expression.

In conclusion, although Eci1 can directly bind to Pex5 in the absence of its PTS1, further work is required to identify the functional role of the additional binding interface we identified. It is plausible that this newly identified binding interface plays a role in dynamic targeting under different metabolic conditions. We now further discuss this point in the manuscript.

OPTIONAL: Similarly, it would have been interesting to see if mutating the residues of PEX5 involved in the interface affect the import of other cargoes than eci1 or if reciprocal mutations in pex5 and Eci1 e.g switching charges could restore an import defect.

We agree that this point is extremely interesting. Future work will be required to address this.

OPTIONAL If 8 & 9 isn't possible could a co-evolutionary analysis of the interface residues provide further independent evidence for their functional importance? They have looked at conservation of residues in Eci1 but this could be extended to a co-evolution analysis.

We agree that this is an interesting point. Future work will be required to shed light on this.

Minor points

Figure 1C and throughout the manuscript state clearly whether the same confocal settings are used when comparing fluorescence intensity of different images/samples.

Done. See the Materials and Methods section.

Figure S2B: Please use different colours for PEX5 and Eci1 for clarity.

In this figure (now Supplementary Figure S7), we present low-resolution density maps for which precise coordinate fitting was not performed. As a result, we cannot determine the exact boundary between Eci1 and Pex5 within the density. Using different colors for Pex5 and Eci1 would imply that we have precisely identified the interface, which is not the case. Therefore, we chose a less restrictive approach, where the Eci1 coordinates from the high-resolution structure are visible through the transparent density. Additionally, we have included Supplementary Figure S11, which highlights the clear electron density maps at the two binding interfaces (PTS1 and EBI).

Figure 4A: please indicate the PTS1 for the other 5 molecules of Eci1. Are they buried? Or not seen? Please add explanation.

Thank you. We now added a clearer explanation in the text as the following: "…There is no supporting electron density for this C-terminal segment, including the PTS1, in the remaining five Eci1 subunits that do not bind Pex5."

Figure 4B, C, and D: please colour the circled helix in PEX5 so that it can be more easily seen.

Done.

Please indicate the EBI-mediated interaction in Figure 4C. The relationship between 4C and 4D could be explained better as they are not viewed from the same direction.

© 2025. Published by The Company of Biologists under the terms of the Creative Commons Attribution License (https://creativecommons.org/licenses/by/4.0/).

We have now modified Figure 4.

Figure S3: As the authors indicated, Pex5 binds with multiple conformations and forms a variable interface with an Eci1 subunit. Does this mean different types of non-canonical interface are possible? Please discuss this.

We have now discussed this further in the text (now Supplementary Figure S8).

Figure 5A and B: they should be labelled as PEX5 TPR domain

The PTS1 binding site is labeled and depicted in detail in Figure 4. In this figure, we chose to focus on displaying the complimentary shape fit of the proteins in the Pex5-Eci1 complex.

Figure S8 is very helpful in understanding the interface and could be included in Figure 5.

We have now improved the visualization of the interface in Figure 4. We have decided to retain this figure as a Supplementary figure S14.

Original submission

First decision letter

MS ID#: jcs.263890

MS TITLE: A Cryo-Electron Microscopy structure of yeast Pex5 in complex with a cargo uncovers a novel binding interface

AUTHORS: Lior Peer; Orly Dym; Nadav Elad; Asa Tirosh; Yossi Jacobovitch; Ehud Sivan; Mor Angel; Shira Albeck; Maya Schuldiner; Yoav Peleg; Einat Zalckvar
ARTICLE TYPE: Review Commons Transfer

Dear Dr Zalckvar,

Many thanks for transferring your manuscript to Journal of Cell Science from Review Commons. I have now had the chance to review your documents, and we would like to invite you to revise your manuscript according to your revision plan. Once we receive the revised version, we may seek further input from the Review Commons referees. If you have any questions about this process, please do get in touch.

While most of the reviewer's comments were carefully addressed, there are two points that still require attention.
The first point has to do with the mutagenesis of the newly identified interface between Pex5 and Eci1. This point was mentioned by all three reviewers, with Reviewers #1 and #2 emphasizing the need for mutagenesis data to confirm that the Pex5-Eci1 interaction within the reported interface is relevant to Eci1 peroxisomal import. While your response clarified that the Eci1 region (F65-L153) is not sufficient for Pex5-dependent import, this does not directly address the reviewers' request, which appears to be a reasonable one.

The second point concerns the SPR data in Figures 3 and S5. It would be important that the data is presented/explained in a manner accessible to a broad cell biology readership. As it stands, it is unclear how relative response over time translates to binding affinities.

© 2025. Published by The Company of Biologists under the terms of the Creative Commons Attribution License (https://creativecommons.org/licenses/by/4.0/).

First revision

Author response to reviewers' comments

1. General Statements

This section is optional. Insert here any general statements you wish to make about the goal of the study or about the reviews.
We want to thank the reviewers for their positive feedback and meaningful input, which dramatically improved the manuscript and helped us to clarify, process, and deepen core points in the manuscript. Please find below the point-by-point response to the reviewers' comments. We believe that our work brings forward insights regarding the complex protein targeting mediated by Pex5 and highlights the intricate and complex nature of protein targeting within eukaryotic cells.
We look forward to working with you towards the publication of this work.

2. Point-by-point description of the revisions

Reviewer #1

(1) The major issue with the paper is that the novel interface between Eci1 and PEX5 has not been demonstrated to be important for import into peroxisomes. Specifically, mutagenesis of both sides of the interface is required to demonstrate that this interaction mediates import of Eci1 lacking the canonical PTS1 signal (and also in the absence of the paralog Dci1). Such data are indisputably a precondition for publication of this paper. Pull-down experiments should also be performed to demonstrate that the interface is sufficient for interacting with PEX5 in the absence of the PTS1 signal on Eci1.

We thank the reviewer for these important points. To answer them:

1.To further verify the direct interaction between Pex5 and Eci1 lacking its PTS1, we performed Biacore analysis to assess the binding of Pex5 to either WT-Eci1 or Eci1 lacking its PTS1 (Figures 3B and Supplementary Figure S4). The results demonstrate that Pex5 can directly bind to Eci1 even in the absence of its PTS1 and independently of Dci1.

2. To further study whether the amino acids mediating the newly identified binding interface with Pex5 are sufficient for peroxisomal targeting, we fused GFP to either the last 10 amino acids of Eci1 (including the PTS1) or to residues F65-L153 of Eci1 (the newly identified binding interface). As expected, the PTS1 of Eci1 was sufficient to mediate peroxisomal targeting in a Pex5- dependent manner. However, the GFP-F65-L153 expression was extremely low, with only a weak GFP signal observed in the cytosol (see figure below).

We hypothesized that the GFP-F65-L153 fusion protein might be misfolded and degraded by the proteasome. To test this, we repeated the experiment in the presence of the proteasome inhibitor MG132. However, the GFP signal remained very weak, suggesting that proteasomal degradation is unlikely to be the primary cause of the low expression.

In conclusion, although Eci1 can directly bind to Pex5 in the absence of its PTS1 and the structural data reveal that the two proteins interact through multiple binding interfaces, further investigation is required to determine the functional significance of the newly identified interface. It is plausible that this additional binding site plays a role in facilitating dynamic targeting under different metabolic conditions. We now elaborate on this point further in the manuscript.

NOTE: We have removed unpublished data that had been provided for the referees in confidence.

The EBI-binding patch of Eci1 is not sufficient to mediate targeting to peroxisomes. Fluorescence microscopy images depicting the N-terminal GFP fusions to either the last

© 2025. Published by The Company of Biologists under the terms of the Creative Commons Attribution License (https://creativecommons.org/licenses/by/4.0/).

10 amino acids of Eci1 (L271-L280, “Last 10 AA of Eci1”) or the residues comprising the newly identified binding interface (F65-L153, “EBI-biding patch”). As expected, GFP- Eci1 PTS1 co-localizes to peroxisomes in a Pex5-dependent manner. However, the newly identified Eci1-binding patch is weakly expressed in the cytosol.

(2) The paper hinges on the demonstration of a residual interaction between PEX5 and Eci1 lacking its PTS1 signal. However, the pull-down experiment in Figure 3 that allegedly shows this result lacks a critical control for non-specific binding of Eci1 to the nickel beads alone. Also, this experiment does not show a direct interaction between PEX5 and Eci1, since the two proteins are co-expressed in bacteria and then pulled down using an engineered His-tag in PEX5. This experiment should be repeated using PEX5 and Eci1 purified separately and then mixed in vitro.

Please show a coomassie-stained SDS-PAGE gel to assess protein purity in addition to the immunoblot, and please show the pull-down in a more conventional way comparing the input and the bound fraction (it is unclear what is meant by soluble and elution fractions).

To address the above concerns, we expressed Pex5 and Eci1 (WT or ΔPTS1) separately. We then mixed Pex5 and Eci1 and performed Biacore analysis to measure their direct interactions (see Figure 3B and Supplementary Figures S4 and S5). The results demonstrate that Eci1 lacking its PTS1 can bind directly to Pex5, however with a weaker interaction compared to full- length Eci1.

(3) The presentation of the structure in Figure 4 should be improved. An overview of the complex should be shown first, and then each interface should be pointed out in a different view (and accordingly labeled). It is distracting and not necessary to show all six subunits of Eci1 in different colors. The non-conventional interface should be shown more clearly, with key amino acids numbered and labeled, and the configurations of their side chains highlighted. Please also highlight the salt bridges and hydrogen bonds at this interface that are mentioned in the text but never illustrated.

Done.

(4) The data in Figs. S2 and S3 raise doubts about the reported resolution of PEX5 in the cryo- EM structure. Please provide examples of the density map and the fit to the model.

Done. We now show density maps in Supplementary Figure S11.

(5) Please provide data for the purification of the complex between PEX5 and Eci1, including a gel-filtration chromatogram and an SDS-PAGE gel of the purified sample used for cryo-EM.

Done. We now added the data in Supplementary Figure S6.

(6) OPTIONAL: The observation that the non-conventional interface between PEX5 with Eci1 corresponds to the site of CoA binding is interesting. This interaction might keep the enzyme inactive while in the cytosol and bound to PEX5, until it would be correctly delivered into peroxisomes and released from the receptor. Alternatively, it could also reflect regulation of Eci1 import by CoA. This idea could easily be tested by pull-down experiments performed with or without CoA, or perhaps by an in vitro Eci1 activity assay in the presence or absence of PEX5. The significance of the paper would be considerably improved if this interaction reflected a mechanism to regulate Eci1 activity or import.

We thank the reviewer for this insightful comment. We find the observation that Pex5 and CoA bind to the same surface extremely interesting. In most of the structures that we identified, only one subunit of the Eci1 hexamer was bound to Pex5 while the remaining five subunits were

© 2025. Published by The Company of Biologists under the terms of the Creative Commons Attribution License (https://creativecommons.org/licenses/by/4.0/).

unbound and potentially available to bind CoA. Under this scenario, Pex5 binding will not completely abolish the activation of Eci1. Additionally, we now discuss an alternative hypothesis suggesting that this mode of interaction facilitates the targeting of CoA to peroxisomes.

Minor issues:

(1) The manuscript has many grammatical mistakes which should be addressed. The absence of line numbers precludes us from indicating specific issues.

We addressed the mistakes.

(2) In general, when referring to a single subunit from the Eci1 hexamer, please use the terms subunit or protomer, and avoid the use of the term monomer which is misleading.

Thank you. We now use the term subunit.

(3) In Fig. 1C, it is unclear whether the experiment was performed in the absence or presence of PEX11. Since the paper hinges on the demonstration of an unconventional interaction between Eci1 and PEX5, perhaps this experiment should be performed in pex11 knockout cells (to enlarge peroxisomes as in Fig. 1B) to show that the residual peroxisomal localization indeed corresponds to the matrix.

The original experiment in which we deleted DCI1 was performed without enlarging peroxisomes. Encouraged by the reviewer's comment, we now repeated the experiment under conditions that promote peroxisome enlargement (see the figure below). Our results confirm that Eci1-mNG is targeted to peroxisomes in the absence of Dci1. Interestingly, we observed that in the absence of Dci1, Eci1-mNG is localized to the peroxisomal membrane and seems to be enriched in specific domains. Using our microscopy setup, we cannot determine whether Eci1- mNG is attached to the membrane from the cytosolic side, embedded within the membrane, or localized in the peroxisomal matrix while interacting with the membrane. Future study beyond the scope of this work is required to determine this point.

NOTE: We have removed unpublished data that had been provided for the referees in confidence.

Eci1-mNG co-localizes to peroxisomes in the absence of Dci1. High-resolution (SORA) fluorescence microscopy images examining the sub-peroxisomal localization of Eci1-mNG on the background of *Δpex11 Δdci1*. Scale: 500 nm

(4) In Fig. 6, it would help to show each structure individually and then the overlay.

Done.

(5) Fig. S4 should include a scale bar and box size.

Fixed (now Supplementary Figure S9).

(6) Why are phosphorylation sites indicated in Fig. S6?

We agree with the reviewer that there is no added value for these indications. We now removed the phosphorylation sites (now Supplementary Figure S12).

(7) In Fig. S8, please show the structures of Eci1 bound to PEX5 and to CoA individually, and then the overlay. The figure is very difficult to understand otherwise.

© 2025. Published by The Company of Biologists under the terms of the Creative Commons Attribution License (https://creativecommons.org/licenses/by/4.0/).

Done (now Supplementary Figure S14).

(8) In Fig. S9, please label the homologous interface residues on Eci1 and Dci1 in individual views, and then show the overlay.

Fixed (now Supplementary Figure S15).

Reviewer #2

1. The authors showed that Pex5 binds to Eci1 in a PTS1 signal-independent manner from pull-down experiments in Figure 2, but this result is qualitative. If the authors add quantitative data on the interaction between Pex5 and Eci1 from isothermal titration calorimetry (ITC) or surface plasmon resonance (SPR), this would make this paper more convincing. This could be done in 2 months.

We thank the reviewer for encouraging us to perform more experiments that will strengthen this point. We now used Biacore analysis to examine and measure the interaction between Pex5 and WT Eci1, and Pex5-Eci1¤PTS1 (Figure 3 and supplementary Figures S4 and S5).

2. It is not clear to what extent the new interaction sites between Pex5 and Eci1 is important for transport to peroxisomes, as revealed in this study. I suggest, for example, expressing Eci1 with a mutation at a site involved in interaction with Pex5 in yeast and analyzing its effect on peroxisomal localization as additional experiments anew. I believe that this could be done in about 2 months.

To further study whether the amino acids mediating the newly identified binding interface with Pex5 are sufficient for peroxisomal targeting, we fused GFP to either the last 10 amino acids of Eci1 (including the PTS1) or to residues F65-L153 of Eci1 (the newly identified binding interface). As expected, the PTS1 of Eci1 was sufficient to mediate targeting to peroxisomes in a Pex5-dependent manner. However, the GFP-F65-L153 expression was extremely low, with only a weak GFP signal observed in the cytosol (See the figure in comment 1 to reviewer 1). We thought that the GFP-F65-L153 fusion protein might be misfolded and degraded by the proteasome. Hence, we repeated the experiment using the proteasome inhibitor MG132. Unfortunately, the signal was still very weak, suggesting that proteasomal degradation is unlikely to be the primary cause of the low expression.
In conclusion, although Eci1 can directly bind to Pex5 in the absence of its PTS1, further work is required to identify the functional role of the additional binding interface we identified. It is plausible that this newly identified binding interface plays a role in dynamic targeting under different metabolic conditions. We now further discuss this point in the manuscript.

Minor comments
1. The results of yeast cell imaging in Figures 1, 2 and S1 are all qualitative and not quantitative. Furthermore, there are no descriptions of the experimental reproducibility of the data. I suggest that these points need to be improved.

We thank the reviewer for this important comment. We performed quantifications for the Eci1- mNG signal (Figures 1, 2, and Supplementary Figure S2).
In each figure, one experiment with three technical repeats is presented; each technical contains at least 100 cells.
We added all the information to the manuscript.

2. I feel that information of sample preparation for cryo-EM analysis of the Pex5-Eci1 complex is not enough since it is only described in the methods. I suggest the authors to add the results of gel-filtration chromatography and CBB-stained SDS-PAGE in the manuscript.

Done (Supplementary Figure S6).

© 2025. Published by The Company of Biologists under the terms of the Creative Commons Attribution License (https://creativecommons.org/licenses/by/4.0/).

3. The authors discuss the interaction interface between Pex5 and Eci1 in Figures 4 and 5, but the manuscript presented at this stage is difficult at least for me to understand the interaction between them. I recommend the authors to add new figure(s) to show more detailed interaction. Also, I suggest that cryo-EM density map around the interaction region between Pex5 and Eci1 should be presented more detail.

Figure 4 was modified and now presents more details of the Pex5-Eci1 interaction. In addition, we added a Cryo-EM density map (Supplementary Figure S11).

Reviewer #3

Most of the experiments presented are well-designed and accompanied with appropriate controls. However, please mention how many times the experiments have been repeated and how many biological samples were used in the analysis.

We thank the reviewer for this important comment.

In each figure, one experiment with three technical repeats is presented; each technical repeat contains at least 100 cells.

We now added all the information to the manuscript.

The authors should also consider the following suggestions substantiate their conclusions: Figure 1A: Include full-length Eci1 with an N-terminal fluorophore, Eci1 PTS1-deletion with N- terminal fluorophore, and the PTS1 deletion with a C-terminal fluorophore, to control for any disturbance of targeting by the C terminal NG tag.

We examined the cellular localization of Eci1 in strains in which Eci1 was either N' or C' tagged with a fluorophore with or without its PTS1 (see figure below). Eci1 co-localized to peroxisomes when it was either tagged on its N' and expressed under an NOP1 promoter or when it was tagged on its C' while expressed under its native promoter. Interestingly, Eci1 was still localized to peroxisomes when its PTS1 was deleted. Notably, the signal of NOP1pr-GFP-Eci1ΔPTS1 was much weaker than the NOP1pr-GFP-Eci1. Nevertheless, the weak signal co-localized to peroxisomes.

Interestingly, when we masked the PTS1 of Eci1 by a fluorophore, we could observe colocalization with peroxisomes even in the absence of Dci1. Nevertheless, we could not detect co-localization with peroxisomes when we deleted the PTS1 in the absence of Dci1.

We decided not to include these results in the manuscript because our microscopy-based setup cannot detect co-localization with peroxisomes when the signal is weak or when it is spread in the cytosol. Hence, it could be that there is a low peroxisomal localization of Eci1 that we cannot detect and hence we cannot interpret the Dci1-dependency without further biochemical work.

NOTE: We have removed unpublished data that had been provided for the referees in confidence.

Eci1 co-localizes to peroxisomes when N' or C' tagged with mNG.

Figure 1C: Confirm the Eci1 and Dci1 levels (if an antibody is available for the latter) by western blot. It is difficult to compare expression levels when comparing just a small number of cells in the microscope. Western blot would give a more robust evaluation of protein levels and help corroborate the claim that Eci1 expression is decreased in the absence of Dci1 if the authors wish to stand by this conclusion.

We apologize for not clearly discussing the results shown in Figure 1C. Our main point is that Eci1-

© 2025. Published by The Company of Biologists under the terms of the Creative Commons Attribution License (https://creativecommons.org/licenses/by/4.0/).

mNG remains co-localized to peroxisomes even in the absence of Dci1. We see that the mNG signal is weaker in the absence of Dci1, and we now quantified this signal from many cells (Supplementary Figure S2A). However, our main focus was not on expression level but rather on the ability of Eci1-mNG to localize to peroxisomes when its PTS1 is masked by mNG and in the absence of Dci1 (which we quantified as well in Figure 1C). We have now modified the text to clarify this point.

Figure 2: confirm the deletion and overexpression of PEX9, PEX5, and PEX7 by western blot of the relevant strains. The production of these strains is not described in the manuscript. If they have been previously described this should be referenced if not it should be included.

We have now added a more detailed explanation regarding creating the KO and overexpression strains (please see "Materials and methods").

Figure 2: Validate these strains by checking import of a canonical PTS1 and canonical PTS2 and pex9 dependent protein to ensure they function as they should, unless these strains have been published elsewhere in which case their characterisation can be referenced.

Done. See Supplementary Figure S3.

Figure 3: The gel should include a standard of a known amount of the lysate used in the pull down to enable a semi-quantitative estimation of the amount of Eci1 protein captured by PEX5 with and without its PTS1. Also include Eci1 with a C-terminal fluorophore to be comparable with the in vivo data in Figs 1 and 2. A control with no pex5 for background would be useful. A full Coomassie-blue stained gel (not western blot) is required to demonstrate the direct interaction as with the western blot it cannot be excluded that other proteins bridge the interaction since this is a pull down from lysate not purified proteins. OPTIONAL:Interestingly the surface on Eci1 which binds pex5 is where CoA binds in the active enzyme. Would CoA compete for binding to Pex5? (could add it into the pull down expt?)

We thank the reviewer for the insightful comments.

We now used Biacore analysis to examine and measure the interaction between Pex5 and WT Eci1, and Pex5-Eci1¤PTS1 (Figure 3B and Supplementary Figures S4 and S5).

We find the observation that Pex5 and CoA bind to the same surface extremely interesting. In most of the structures that we identified, only one Eci1 subunit was bound to Pex5, while the other five subunits were unbound to Pex5 and potentially available to bind CoA. We expect that under this scenario, Pex5 binding will not completely abolish the activation of Eci1. Additionally, we have now discussed another possibility that this mode of interaction facilitates the targeting of CoA to peroxisomes.

Figure S2: The complex between pex5 and eci1 is solved by cryo EM. Eci1 is hexameric usually 1 but sometimes 2 or 3 pex5s are bound to the complex. The size-exclusion chromatography figure with calculated molecular weight is required to support the stoichiometry. A native gel to show the complex, as well as a denaturing gel (using the complex) to show the individual proteins will be beneficial.

Done. Please see Supplementary Figure S6.

Figure S9: Would Eci1 compete with Dci1 to bind to Pex5 since they share highly conserved interfaces? If so, why did the deletion of Dci1 impair Eci1 location? Or is this just reduced expression in the dci1 deletion background? (See point 2) This seems counterintuitive/contradictory so please comment.

© 2025. Published by The Company of Biologists under the terms of the Creative Commons Attribution License (https://creativecommons.org/licenses/by/4.0/).

We apologize for not being clear. We show, in line with previous works, that in the absence of Dci1, Eci1-mNG is still localized to peroxisomes although it seems like its expression is lower.

OPTIONAL: As the authors acknowledge this work is in vitro. It would have been interesting to examine the role of this interface in vivo by mutating one or more of the residues in Eci 1 identified as being important for the interaction. Granted that mutation can affect the folding of the protein, but the binding region is on the surface so it may not, and this can be readily checked e.g by enzyme activity or limited proteolysis.

To further study whether the amino acids mediating the newly identified binding interface with Pex5 are sufficient for peroxisomal targeting, we fused GFP to either the last 10 amino acids of Eci1 (including the PTS1) or to residues F65-L153 of Eci1 (the newly identified binding interface). As expected, the PTS1 of Eci1 was sufficient to mediate targeting to peroxisomes in a Pex5-dependent manner. However, the GFP-F65-L153 expression was extremely low, with only a weak GFP signal observed in the cytosol (See the figure in comment 1 to reviewer 1). We thought that the GFP-F65-L153 fusion protein might be misfolded and degraded by the proteasome. Hence, we repeated the experiment using the proteasome inhibitor MG132. Unfortunately, the signal was still very weak, suggesting that proteasomal degradation is unlikely to be the primary cause of the low expression.
In conclusion, although Eci1 can directly bind to Pex5 in the absence of its PTS1, further work is required to identify the functional role of the additional binding interface we identified. It is plausible that this newly identified binding interface plays a role in dynamic targeting under different metabolic conditions. We now further discuss this point in the manuscript.

OPTIONAL: Similarly, it would have been interesting to see if mutating the residues of PEX5 involved in the interface affect the import of other cargoes than eci1 or if reciprocal mutations in pex5 and Eci1 e.g switching charges could restore an import defect.

We agree that this point is extremely interesting. Future work will be required to address this.

OPTIONAL If 8 & 9 isn't possible could a co-evolutionary analysis of the interface residues provide further independent evidence for their functional importance? They have looked at conservation of residues in Eci1 but this could be extended to a co-evolution analysis.

We agree that this is an interesting point. Future work will be required to shed light on this.

Minor points

Figure 1C and throughout the manuscript state clearly whether the same confocal settings are used when comparing fluorescence intensity of different images/samples.

Done. See the Materials and Methods section.

Figure S2B: Please use different colours for PEX5 and Eci1 for clarity.

In this figure (now Supplementary Figure S7), we present low-resolution density maps for which precise coordinate fitting was not performed. As a result, we cannot determine the exact boundary between Eci1 and Pex5 within the density. Using different colors for Pex5 and Eci1 would imply that we have precisely identified the interface, which is not the case. Therefore, we chose a less restrictive approach, where the Eci1 coordinates from the high-resolution structure are visible through the transparent density. Additionally, we have included Supplementary Figure S11, which highlights the clear electron density maps at the two binding interfaces (PTS1 and EBI).

Figure 4A: please indicate the PTS1 for the other 5 molecules of Eci1. Are they buried? Or not seen? Please add explanation.

Thank you. We now added a clearer explanation in the text as the following: "…There is no

© 2025. Published by The Company of Biologists under the terms of the Creative Commons Attribution License (https://creativecommons.org/licenses/by/4.0/).

supporting electron density for this C-terminal segment, including the PTS1, in the remaining five Eci1 subunits that do not bind Pex5.”

Figure 4B, C, and D: please colour the circled helix in PEX5 so that it can be more easily seen.

Done.

Please indicate the EBI-mediated interaction in Figure 4C. The relationship between 4C and 4D could be explained better as they are not viewed from the same direction.

We have now modified Figure 4.

Figure S3: As the authors indicated, Pex5 binds with multiple conformations and forms a variable interface with an Eci1 subunit. Does this mean different types of non-canonical interface are possible? Please discuss this.

We have now discussed this further in the text (now Supplementary Figure S8).

Figure 5A and B: they should be labelled as PEX5 TPR domain

The PTS1 binding site is labeled and depicted in detail in Figure 4. In this figure, we chose to focus on displaying the complimentary shape fit of the proteins in the Pex5-Eci1 complex.

Figure S8 is very helpful in understanding the interface and could be included in Figure 5.

We have now improved the visualization of the interface in Figure 4. We have decided to retain this figure as a Supplementary figure S14.

Second decision letter

MS ID#: jcs263890R1

MS TITLE: A Cryo-Electron Microscopy structure of yeast Pex5 in complex with a cargo uncovers a novel binding interface

AUTHORS: Lior Peer; Orly Dym; Nadav Elad; Asa Tirosh; Yossi Jacobovitch; Ehud Sivan; Mor Angel; Shira Albeck; Maya Schuldiner; Yoav Peleg; Einat Zalckvar
ARTICLE TYPE: Review Commons Transfer

Dear Dr Zalckvar,

I am happy to tell you that your manuscript has been accepted for publication in Journal of Cell Science, pending standard publication integrity checks.

Thank you for sending your manuscript to Journal of Cell Science through Review Commons.

© 2025. Published by The Company of Biologists under the terms of the Creative Commons Attribution License (https://creativecommons.org/licenses/by/4.0/).
